# Olfaction-Related Gene Expression in the Antennae of Female Mosquitoes From Common *Aedes aegypti* Laboratory Strains

**DOI:** 10.3389/fphys.2021.668236

**Published:** 2021-08-23

**Authors:** Soumi Mitra, Matthew Pinch, Yashoda Kandel, Yiyi Li, Stacy D. Rodriguez, Immo A. Hansen

**Affiliations:** ^1^Department of Biology, New Mexico State University, Las Cruces, NM, United States; ^2^Department of Computer Science, New Mexico State University, Las Cruces, NM, United States

**Keywords:** olfaction, repellent, strains, antenna, transcriptome, *Aedes aegypti*

## Abstract

Adult female mosquitoes rely on olfactory cues like carbon dioxide and other small molecules to find vertebrate hosts to acquire blood. The molecular physiology of the mosquito olfactory system is critical for their host preferences. Many laboratory strains of the yellow fever mosquito *Aedes aegypti* have been established since the late 19th century. These strains have been used for most molecular studies in this species. Some earlier comparative studies have identified significant physiological differences between different laboratory strains. In this study, we used a Y-tube olfactometer to determine the attraction of females of seven different strains of *Ae. aegypti* to a human host: UGAL, Rockefeller, Liverpool, Costa Rica, Puerto Rico, and two odorant receptor co-receptor (Orco) mutants Orco2 and Orco16. We performed RNA-seq using antennae of Rockefeller, Liverpool, Costa Rica, and Puerto Rico females. Our results showed that female *Aedes aegypti* from the Puerto Rico strain had significantly reduced attraction rates toward human hosts compared to all other strains. RNA-seq analyses of the antenna transcriptomes of Rockefeller, Liverpool, Costa Rica, and Puerto Rico strains revealed distinct differences in gene expression between the four strains, but conservation in gene expression patterns of known human-sensing genes. However, we identified several olfaction-related genes that significantly vary between strains, including receptors with significantly different expression in mosquitoes from the Puerto Rico strain and the other strains.

## Introduction

The chemical sense of olfaction plays an important role in the life of all insects, not only in locating food sources, but also in other sensory-mediated behaviors like mating and egg deposition (Fleischer and Krieger, 2018). Olfaction in insects occurs primarily in their antennae, but other structures including the mouth parts, wing fringes, and tarsal segments also house chemosensory sensor systems (Dunipace et al., 2001; Scott et al., 2001; Thorne et al., 2004; Wang et al., 2004; Dahanukar et al., 2007; Vosshall and Stocker, 2007; Carey and Carlson, 2011; Sparks et al., 2013, 2014; Bohbot et al., 2014). These olfactory regions are covered with sensilla, small hair-like structures containing odor-sensing neurons called olfactory receptor neurons (ORNs) (Carey and Carlson, 2011). ORNs within the sensilla express chemosensory receptor proteins from three protein families that detect specific types of odorants. Ionotropic receptors (IRs) are ion channels (Leal, 2013; Fleischer et al., 2018), while odorant receptors (ORs) and gustatory receptors (GRs) are structurally similar to G-protein coupled receptors (Wicher, 2018). ORs form heteromers with an odorant receptor co-receptor protein (ORCO) (Fleischer et al., 2018). This OR-ORCO complex functions as a ligand gated ion channel for odor sensation (Vosshall and Hansson, 2011). Odorant-binding proteins (OBPs) are a family of globular proteins found in the sensillar lymph and elsewhere that are thought to play an important role in chemoreception by solubilizing hydrophobic odorants (Biessmann et al., 2010). Previous research has shown that members of these aforementioned protein families are expressed in the antennae of insects (Brito et al., 2016; Pelosi et al., 2018; Venthur and Zhou, 2018) including *Aedes aegypti* (Matthews et al., 2016).

During host-seeking, female mosquitoes follow olfactory cues that they detect with sensilla located on their antennae and maxillary palps (Roth, 1951; Bohbot et al., 2014). Laboratory studies of *Ae. aegypti* olfaction demonstrated that lactic acid, carbon dioxide, and a variety of carboxylic acids attract female mosquitoes (Acree et al., 1968; Carlson et al., 1973; Takken and Knols, 1999; Geier and Boeckh, 2003). While lactic acid is known to be a prominent attractive odor, there are conflicting reports on the necessity of CO_2_ as a co-odorant with lactic acid to stimulate attraction (Acree et al., 1968; Geier and Boeckh, 2003). While it is possible that observed differences in odorant-stimulated attraction may be due to differences in experimental technique, it cannot be ruled out that the difference in observed responses to the odorant stimuli may be due to use of different laboratory strains of *Ae. aegypti* in each study.

Many behavioral and physiological experiments are conducted on animal strains that have been bred under laboratory conditions for generations. In the case of the insect model organism, *Drosophila melanogaster*, 308 wildtype strains alone are maintained in the Bloomington *Drosophila* Stock Center. Even though these strains are members of the same species, differences in important phenotypic characteristics such as locomotion, and differences in gene expression patterns in such important functional pathways such as metabolism and synaptic transmission exist between strains (Colomb and Brembs, 2014; Zarubin et al., 2020). *Ae. aegypti* is a favored mosquito species for laboratory studies around the world as field-collected specimens are relatively easy to introduce and propagate in laboratory culture compared to other mosquito species. A large number of different laboratory strains of *Ae. aegypti* have been established from different geographical origins in the last century (Kuno, 2014). As with *Drosophila*, diversity between different laboratory strains has been addressed in experimental studies, many with focus on insecticide-resistance, susceptibility to pathogens, or other traits (Gómez et al., 2011; Costa-da-Silva et al., 2017; Gloria-Soria et al., 2019).

*Aedes aegypti* can transmit a variety of medically important diseases when they take a blood meal from a human host. Therefore, it is important to deepen our understanding of how olfactory gene expression affects host seeking behavior as an avenue for controlling mosquito attraction to humans. As a variety of *Ae. aegypti* strains are used for physiology and behavior studies, an understanding of differences in behavior and gene expression is necessary for ensuring that results from studies using different strains produce results that can be applied to the species as a whole. In this study, we demonstrate differences in attraction to human odor between different laboratory strains of *Ae. aegypti* mosquitoes using a Y-tube olfactometer bioassay. Furthermore, we use RNA-seq and quantitative RT-PCR to compare olfactory gene expression in antennae of four of these strains.

## Materials and Methods

### Mosquito Strains

Table 1 shows the origin of the seven laboratory strains of *Ae. aegypti* used in this study. Six strains were obtained from BEI Resources (Molestina, 2010), and UGAL mosquitoes were a generous gift from Alexander Raikhel at UC Riverside. All strains were maintained for no less than four generations after arrival in our insectary before being used for experiments.

**TABLE 1:** Strains used in this study.

**STRAIN**	**SOURCE**	**BEI-ORDER#**	**Presumptive region of origin**
Rockefeller	BEI	MRA-734	Cuba
Liverpool	BEI	NR-48921	West Africa
Costa Rica	BEI	MRA-726	Costa Rica
UGAL	UCR	n/a	Georgia, United States
Puerto Rico	BEI	NR-48830	Puerto Rico
Orco2	BEI	NR-44376	Florida
Orco16	BEI	NR-44378	Florida

*BEI, BEI resources; UCR, University of California, Riverside.*

### Mosquito Culture

Mosquito eggs of each strain were dried and kept for 1 week after being laid. Eggs from each strain were hatched separately in 13″× 20″ pans, in deionized water at 27°C. Every third day, mosquito larvae were fed dry cat food pellets (Special Kitty, Walmart Stores Inc., Bentonville, AR, United States). The water in the pans was changed every fifth day. Adult mosquitoes were reared for 5 days in Bug Dorm-1 insect rearing cages (30 × 30 × 30 centimeters, BugDorm, Taichung, Taiwan) under controlled conditions (27°C, 80% humidity, 14:10 h light:dark cycle), and were maintained on 20% sucrose solutions *ad libitum*.

### Y-Tube Olfactometer Bioassay

**FIGURE 1:**
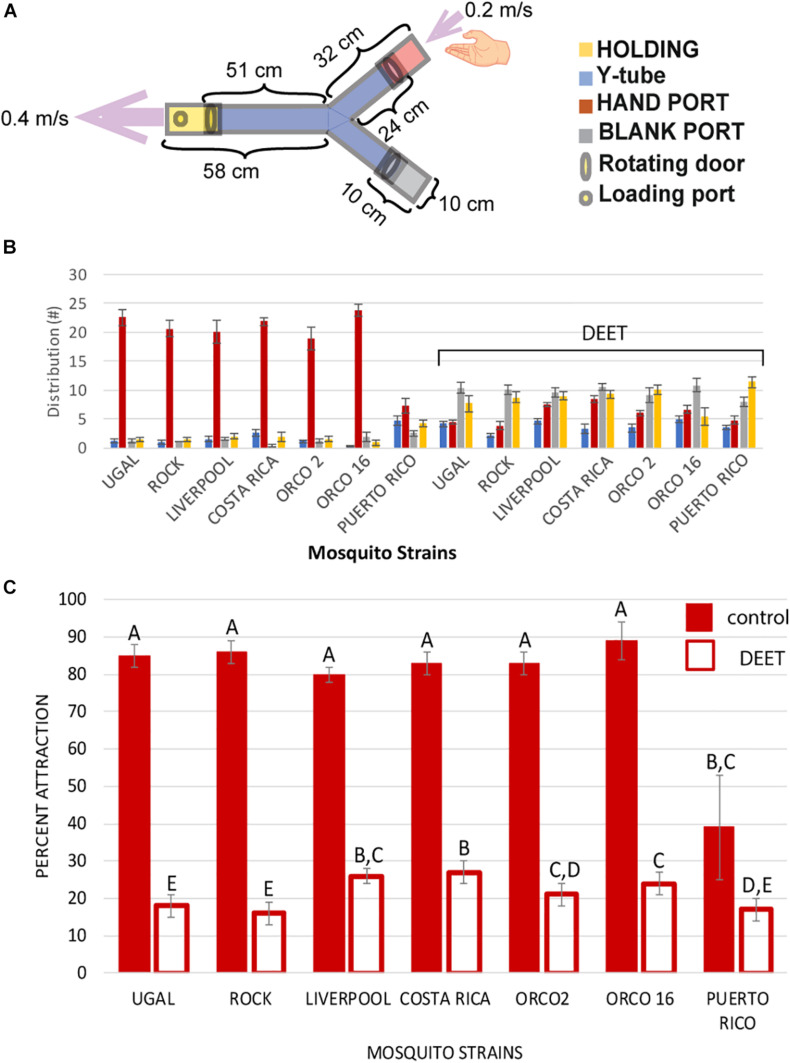
Mosquito attraction bioassay using a Y-tube olfactometer. **(A)** Diagram of Y-tube olfactometer. Dimensions are given in centimeters. Arrows and values represent direction and speed of airflow. **(B)** Distribution of mosquitoes in each Y-tube chamber at the end of each assay. Strains were exposed to either a control hand (left set), or a DEET treated hand (right set under “DEET” bracket). Data is presented as average number of mosquitoes from four treatments in each chamber ± SEM. Column colors correspond to chamber colors in the legend of **(A)**. **(C)** Percent attraction of mosquito strains to the control hand (red columns) and the DEET-treated hand (empty columns) for each mosquito strain. Data is presented as average percent attraction from four treatments ± SEM. Letters above columns represent statistical significance categories determined by Mann–Whitney *U* tests, with different letters representing significantly different (*p* < 0.05) attraction rates. See section “Materials and Methods” Y-tube olfactometer bioassay for a description of how percent attraction was determined.

A plexiglass Y-tube olfactometer (Figure 1A) was constructed according to WHO instructions with some changes (Rodriguez et al., 2015). All Y-tube assays were begun at 8:00 am (zeitgeber time 3). Around 25–30 1-week old female mosquitoes were starved overnight and released into the “Holding” chamber of the Y-tube. A volunteer’s hand was placed at the open end of the “Hand” chamber while the other “Blank” chamber was left empty. A computer fan was placed five cm from the holding chamber to pull air through the Y-tube and the airspeed was measured at different locations within the tube using a digital anemometer (TPI565, Test Products International, Beaverton, OR, United States) to ensure that airspeed in the base of the Y-tube was 0.4 m/s, and airspeed at the hand and blank ports was 0.2 m/s (Rodriguez et al., 2015). After 30 s elapsed, all chamber doors were opened, and mosquitoes were allowed to fly for the next 2 min. At the end of 2 min, all the doors were closed, and the total number of mosquitoes present in each chamber were counted and recorded. Percent attraction was calculated as:

$$\text{Attraction}\%=\frac{\#\,\mathrm{mosquitoes}\,\text{in}\,"\,\mathrm{Hand}\,"}{\text{total}\,\#\,\text{mosquitoes}}\times 100$$

Each strain was tested once per day for 4 days to generate four biological replicates per strain. Another set of Y-tube olfactometer bioassays were conducted using 0.5 milliliter Ben’s^®^ 100% DEET (N, N-diethyl toluamide) (Adventure Ready Brands^TM^, Littleton, NH, United States) applied to the volunteer’s hand before placing it adjacent to the “Hand” chamber. DEET was used to determine any reductions in percent attraction of each strain in this experiment. Significant differences in attraction rates between strains and treatments were determined by Mann–Whitney *U* tests using GraphPad Prism8 (GraphPad Software, San Diego, CA, United States).

### RNA-Seq Sample Preparation

Female mosquitoes were anesthetized using CO_2_ and antennae and pedicels containing the Johnston’s organs were dissected. Three groups of 100 adult female mosquitoes (200 antennae) of each of four strains, Rockefeller, Liverpool, Cost Rica, and Puerto Rico, were dissected. Antennae were homogenized in 500 μL TRI Reagent^®^ (Sigma-Aldrich, St. Louis, MO, United States) using a VWR cordless motor (VWR, cat. No. 4774-370) and disposable polybutylene terephthalate pestles (VWR, cat. No. 4774-358) for 5 min prior to total RNA extraction following the manufacturer’s instructions. RNA samples were shipped to GeneWiz (South Plainfield, NJ, United States) for Illumina HiSeq 150 bp paired end RNA-seq analysis using their Standard RNA-seq service.

### Library Preparation and Sequencing

RNA samples were quantified using a Qubit 2.0 Fluorometer (Life Technologies, Carlsbad, CA, United States) and RNA integrity was checked using the Agilent TapeStation 4200 (Agilent Technologies, Palo Alto, CA, United States). RNA sequencing libraries were prepared using the NEBNext Ultra RNA Library Prep Kit for Illumina using manufacturer’s instructions (NEB, Ipswich, MA, United States). Briefly, mRNAs were initially enriched with Oligod(T) beads and fragmented for 15 min at 94°C. First strand and second strand cDNA were subsequently synthesized. cDNA fragments were end repaired and adenylated at 3′ends, and universal adapters were ligated to cDNA fragments, followed by index addition and library enrichment by PCR with limited cycles. The sequencing library was validated on the Agilent TapeStation (Agilent Technologies, Palo Alto, CA, United States), and quantified by using a Qubit 2.0 Fluorometer (Invitrogen, Carlsbad, CA, United States) as well as by quantitative PCR (KAPA Biosystems, Wilmington, MA, United States). The sequencing libraries were clustered on a single lane of a flowcell which was loaded on the Illumina HiSeq instrument (4000 or equivalent) according to manufacturer’s instructions. The samples were sequenced using a 2 × 150 bp Paired End (PE) configuration. Image analysis and base calling were conducted by the HiSeq Control Software (HCS). Raw sequence data (.bcl files) generated from Illumina HiSeq was converted into fastq files and de-multiplexed using Illumina’s bcl2fastq 2.17 software. One mismatch was allowed for index sequence identification.

### Mapping Sequence Reads to the Reference Genome

Sequence reads were trimmed to remove possible adapter sequences and nucleotides with poor quality using Trimmomatic v.0.36 (Bolger et al., 2014). The trimmed reads were mapped to the *Ae. aegypti* reference genome (AaegL5.3) available on ENSEMBL using the STAR aligner v.2.5.2b (Dobin et al., 2013). Unique gene hit counts were calculated by using featureCounts from the Subread package v.1.5.2 (Liao et al., 2013, 2014). Only unique reads that fell within exon regions were counted. Fragments per kilobase of transcript per million reads (FPKM) values for all reads were generated by GeneWiz.

### Gene Identification and Annotation

Gene RefSeq IDs were obtained from GeneWiz and mapped to the BioMart database from VectorBase^1^. Gene function information (Interpro descriptions, gene descriptions, and GO terms) was obtained from the most recent *Ae. aegypti* genome annotation (*Aedes aegypti* Liverpool genome v5.3) in BioMart. All sensory genes in our transcriptomes were identified by first searching Vectorbase for odorant-related genes using the following description categories, Product.Description, Interpro.Description, Smart.Description, Superfamily.Description, TigrFam.Description, Prositefamilies. Description, PirSF.Description, PFam.Description to identify genes already annotated as olfaction-related, or putative genes with structural similarity. Additionally, this set of genes was cross-referenced to annotated olfaction-related genes in recent publications classifying *Ae. aegypti* sensory genes (Matthews et al., 2016, 2018; Hill et al., 2021), to ensure a comprehensive dataset. As a result of cross-referencing to recent publications, 36 additional genes were added. Genes with zero expression in all samples were filtered out to complete the dataset used for analysis.

### Differential Expression and Statistical Analysis

After extraction of gene hit counts, the gene hit counts table was used for downstream differential expression analysis. Based on their alignment result and annotation, BioMart from vectorbase.org was used to add gene descriptions and RNA lengths of each gene. FPKM data was log scaled and used to perform analysis on the whole transcriptome dataset including principal component analysis (PCA), comparisons between gene families, and median FPKM values of transcriptomes from the four strains. The RNA-seq FPKM data was discretized with the R package Ckmeans.1d.dp (Wang and Song, 2011; Song and Zhong, 2020). Differential gene expression of individual transcripts from each strain was determined using un-scaled FPKM values and chi-square goodness of fit tests to compare gene expression in one strain to all three other strains (*p* < 0.05). Because any given OR, GR, IR, or OBP are only expressed in a small number of cells in the antenna, the abundance of specific fragments of olfaction-related genes in our libraries is likely to be relatively low. We decided to include all odorant related genes that were detected in at least one sample in order to gain a comprehensive picture of strain-specific differences.

### Quantitative Real Time PCR

Quantitative real time PCR (qRT-PCR) was performed on a small subset of olfactory genes to validate RNA-seq data. Three genes with elevated expression in PR mosquitoes as determined by RNA-seq (*obp56a*, *or26*, and *or97*), and two candidate internal reference genes (β-*actin* and *rps7*) (Supplementary File 1) were selected for analysis. qRT-PCR analysis was performed on the same four strains as RNA-seq. Antennae and pedicels containing Johnston’s organs from three groups of 100 mosquitoes (200 antennae per group) per strain were dissected and stored in TRI Reagent^®^ (Sigma-Aldrich Corp., St. Louis, MO, United States) followed by purification and in-column DNase I treatment using an RNA Clean and Concentrator^TM^-100 kit (Zymo Research, Irvine, CA, United States). Total RNA was reverse-transcribed using iScript^TM^ Reverse Transcription Supermix for RT-qPCR (Bio-Rad, Hercules, CA, United States) to generate cDNA templates. To confirm the absence of genomic DNA, non-reverse transcribed (noRT) samples were generated using noRT Supermix. Gene specific primers (Supplementary File 1) were designed using Primer-BLAST (Ye et al., 2012) and evaluated with NetPrimer (Premier Biosoft, Palo Alto, CA, United States). Primers were designed to flank intron sequences to allow for discrimination between mRNA and genomic DNA amplification products. Qualitative PCR amplification was performed for all primer pairs on both RT and noRT samples using Taq 2X Master Mix (New England Biolabs, Ipswich, MA, United States) prior to qRT-PCR. Qualitative PCR products were visualized on 1% agarose gels stained with SYBR^®^ Safe (Invitrogen, Carlsbad, CA, United States) to verify correct amplicon size.

Quantitative real time PCR was performed using the iTaq Universal SYBR^®^ Green One-Step Kit (Bio-Rad, Hercules, CA, United States). Two technical replicates of each sample were performed. Samples were analyzed in 96-well plates with Masterclear real-time PCR Adhesive Film (Eppendorf, Hamburg, Germany) in a Bio-Rad CFX96 Touch Deep Well Real-Time PCR Detection System (Bio-Rad, Hercules, CA, United States) using a protocol consisting of an initial denaturation at 95°C for 30 s. followed by 40 cycles of 95°C denaturation for 5 s, and a combined annealing/elongation step at 60°C for 30 s. Fluorescence measurement was performed after each elongation step. Immediately following the PCR, a melting curve analysis was performed from 60°C to 95°C in 0.5°C increments with a 5 s. hold at each step. qRT-PCR and melting curve data was collected using Bio-Rad CFX Maestro software (Bio-Rad, Hercules, CA, United States). C_q_ values were analyzed using the RefFinder analysis tool^2^ (Xie et al., 2012), which uses four algorithms (Vandesompele et al., 2002; Andersen et al., 2004; Pfaffl et al., 2004; Silver et al., 2006) to identify the most appropriate reference gene based on stability of gene expression. Primer pair amplification efficiency was determined by analyzing raw fluorescence data using the Real-Time PCR Miner tool^3^ (Zhao and Fernald, 2005). C_q_ values were imported from the Bio-Rad CFX Maestro program into Microsoft Excel and averaged C_q_ values from technical replicates of each sample were analyzed using calculated primer pair efficiency adjustment and normalized against the reference gene, *rps7* (a ribosomal subunit), to derive relative mRNA levels between strains. Statistical analysis of relative mRNA levels was performed using GraphPad Prism (GraphPad Software, San Diego, CA, United States). Shapiro–Wilk tests for normality were performed prior to one-way ANOVA and Tukey’s multiple comparisons *post hoc* tests to determine significant differences in relative mRNA levels between each strain.

## Results

### Long-Range Attraction Assay

We used a choice assay with a Y-tube olfactometer (Figure 1A) to determine attraction rates of adult females from different laboratory strains of *Ae. aegypti* (Rodriguez et al., 2015). Six of the seven laboratory strains showed strong attraction toward the human hand that was used as bait (Figures 1B,C). Females from the insecticide-resistant PR strain showed significantly reduced attraction to the human hand bait compared to all other strains. When the human hand was treated with DEET, prior to the experiment, females from all strains showed a significant reduction in attraction (Figures 1B,C).

### General RNA-Seq Results

**FIGURE 2:**
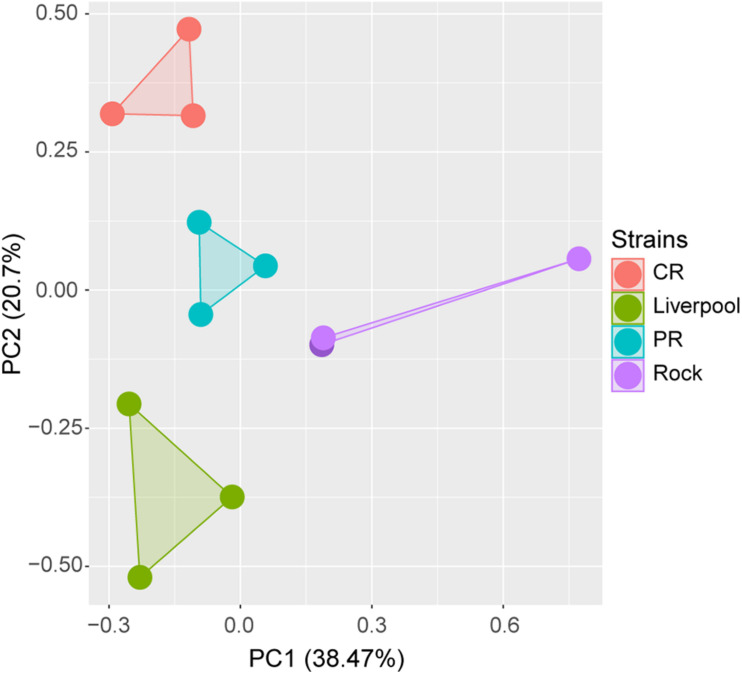
Principal component analysis of variability of gene expression in RNA-seq data from four *Ae. aegypti* strains. Points represent individual biological replicates from each strain.

In total, the RNA-seq analysis produced a mean of 30,596,079 reads per sample (367,152,946 total reads), yielding a mean of 9,179 Mbases per sample (110,146 total Mbases) (Supplementary File 2). The mean percent of bases with a quality score over 30, indicating a 99.9% confidence in call accuracy, was 86.61% across all samples, with a low of 85.9% and a high of 87.58% (Supplementary File 2). The mean quality score across all samples was 35.8, with a low of 35.65 and a high of 36.05 (Supplementary File 2). After adapter trimming and removal of low-quality base pairs, an average of 28,918,212 total reads per sample remained, of which an average of 22,725,239 were unique mapped reads (Supplementary File 2). FPKM of all sensation-associated genes in each sample were generated and included the following chemosensory gene categories: odorant receptors (ORs) and the obligate odorant receptor co-receptor (ORCO), gustatory receptors (GRs), ionotropic receptors (IRs), and odorant binding proteins (OBPs), as well as other genes associated with olfaction including pickpocket (PPK) and transient receptor potential (TRP) channels. Principal component analysis based on the RNA-seq data showed all four strains clustering separately, indicating distinct differences in gene expression profiles between the different laboratory strains (Figure 2). All sequence files for each strain have been uploaded to NCBI BioProject: PRJNA715771.

### Olfaction-Related Gene Expression

#### Odorant Receptors (ORs)

**FIGURE 3:**
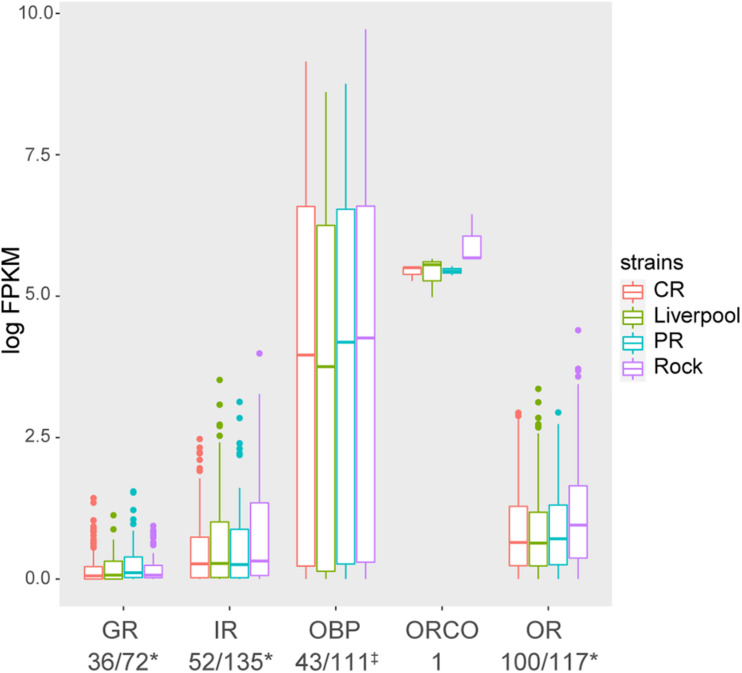
Boxplots of total expression of different olfactory related genes detected in the transcriptomes of four *Ae. aegypti* strains. Olfactory related genes belonging to five categories: odorant receptors (ORs), gustatory receptors (GRs), ionotropic receptors (IRs), odorant receptor co-receptor (ORCO), and odorant binding proteins (OBPs) were detected. Boxplots represent the interquartile range of RNA expression from three biological replicates per strain in log scaled FPKM. Lines in each box represent mean RNA expression, vertical lines represent first and fourth quartiles, and dots represent outliers. Numbers beneath each label represent the number of transcripts detected/total number of genes annotated either by Matthews et al. (2018) (denoted with an asterisk) or Manoharan et al. (2013) (denoted with^‡^).

In our RNA-seq analysis of olfaction related genes on Vectorbase, we identified 100 out of 117 *Ae. aegypti* ORs annotated by Matthews et al. (2018) with expression in at least one sample (Figure 3 and Supplementary File 3). Overall, Rock mosquitoes had slightly higher OR expression than the other three strains (Figure 3). Chi square analysis of olfaction-related gene expression in individual strains compared to all other strains revealed several ORs that were significantly increased or decreased (*p* < 0.05) in each particular strain. In Rock, we identified six ORs [*or122* (AAEL013563), *or125* (AAEL013893), *or116* (AAEL025139), *or13* (AAEL008368), *or28* (AAEL027053), and *or81* (AAEL017305)] with significantly increased expression compared to the other three strains (Supplementary File 5). Liverpool mosquitoes had two ORs [*or40* (AAEL005767) and *or102* (AAEL023017)] with significantly increased expression compared to the other three strains (Supplementary File 6). We did not identify any ORs with significantly different expression in CR mosquitoes compared to the other strains (Supplementary File 7). Finally, PR mosquitoes had one OR [*or103* (AAEL017505)] with significantly increased expression compared to the other three strains (Supplementary File 8). ORCO, initially annotated as *or7* (Melo et al., 2004), was expressed at high levels in all strains, but its expression was increased in Rock (Figure 3).

The OR ligand repertoire of *Ae. aegypti* is not well classified, but we have found reports of seven *Ae. aegypti* ORs that have been experimentally determined to respond to human odors. We identified all seven of these ORs in our transcriptome dataset. Sulcatone-sensitive *or4* (AAEL015147) (McBride et al., 2014) was the highest expressed of these seven ORs in all four strains, with average FPKM values of 14.9 in CR, 5.4 in Liverpool, 8.6 in PR and 26.4 in Rock (Supplementary Files 3, 4). 3-methylindole-sensing *or10* (AAEL006003) (Bohbot and Pitts, 2015; Ruel et al., 2019) and indole-sensing *or2* (AAEL005999) (Bohbot and Dickens, 2010; Bohbot et al., 2011) were the second and third highest expressed of the known human-sensing ORs, respectively (Supplementary Files 3, 4). The final four human-sensing ORs, *or14* (AAEL008442) (Zeng et al., 2019), *or15* (AAEL008448) (Zeng et al., 2019), *or9* (AAEL006005) (Bohbot and Pitts, 2015), and *or8* (AAEL012254) (Bohbot and Dickens, 2009) were the lowest expressed across all strains, with *or8* expression being higher in CR and *or15* expression being lower in PR relative to the other strains (Supplementary Files 3, 4).

#### Gustatory Receptors (GRs)

Out of 72 *Ae. aegypti* GRs annotated by Matthews et al. (2018), we were able to detect 36 in our antennae sensory transcriptome that were expressed in at least one sample (Figure 3 and Supplementary File 3). Overall, GRs had the lowest expression of the five major sensory gene classes surveyed in this study (Figure 3) with only eight of the 36 GRs having an expression >1 FPKM in at least one sample (Supplementary File 3). GR genes *gr1*, *gr2*, and *gr3* have previously been reported to form a CO_2_-sensing complex in the maxillary palps (Erdelyan et al., 2012; Ray, 2015; Fleischer et al., 2018; Kumar et al., 2020). Interestingly, all three of these GRs were among the seven with expression >1 FPKM in at least one sample of our antennal transcriptomes (Supplementary File 3). We identified several GRs with significantly different expression in different strains, with *gr66* (AAEL017394) having elevated expression in Liverpool (Supplementary File 6), *gr2* (AAEL002167) having significantly higher expression in CR (Supplementary File 7), and *gr45* (AAEL006494) being significantly increased in PR (Supplementary File 8).

#### Ionotropic Receptors (IRs)

We identified 52 IRs out of 135 *Ae. aegypti* IRs annotated by Matthews et al. (2018) with expression in at least one sample (Figure 3). Twenty-nine IRs have previously been identified as antennal IRs in female *Ae. aegypti* (Tallon et al., 2019), of which we identified 25 in our dataset. No antennal IRs were significantly different between strains, and only *ir101* (AAEL022101) showed significantly different expression, being significantly elevated in Rock mosquitoes (Supplementary File 5). Of the antennal IRs, *ir8a* (AAEL002922), *ir87a.2* (AAEL011690), *ir75o* (AAEL014086), *ir75k.1* (AAEL014089), *ir41k* (AAEL000066), and *ir92a* (AAEL011001) had elevated expression in Rock compared to the other strains (Supplementary File 4). Several antennal IRs, *ir75o* (AAEL014086), *ir75e.3* (AAEL010775), *ir75e.3* (AAEL010775), *ir75i* (AAEL013198), *ir75k.2* (AAEL024757), *ir41p.2* (AAEL018060), *ir41i* (AAEL000047), and *ir25a* (AAEL009813) had lower expression in CR relative to the other strains (Supplementary File 4), and three antennal IRs, *ir92a* (AAEL011001), *ir76b* (AAEL006360), and *ir87a.1* (AAEL011691) had lower expression in PR than the other strains (Supplementary File 4).

#### Odorant-Binding Proteins (OBPs)

We detected 43 out of 111 OBPs annotated by Manoharan et al. (2013) with expression in at least one sample in this study. Our analysis showed high variability in expression of different OBPs in each strain, but the overall expression distribution was similar across all four strains (Figure 3). Substrates for two *Ae. aegypti* OBPs have been previously identified. We detected both of these OBPs at high levels in all samples of or transcriptome data. The long chain fatty acid-sensitive *obp22* (AAEL005772) (Wang et al., 2020) was expressed at similar levels in all strains, while the oviposition pheromone-sensing *obp39* (AAEL009449) (Leal and Leal, 2015) was decreased in Liverpool and Rock samples (Supplementary File 4). Despite the wide range of OBP expression in each strain (Figure 3), we only identified one significantly differentially expressed OBP, *obp47* (AAEL011499), which had higher expression in CR relative to the other strains (Supplementary File 7).

#### Other Proteins Implicated in Odor Sensation

We also identified members of non-canonical chemosensory receptor PPK and TRP channel families in our dataset. These receptor types encode gated transmembrane sodium (PPK) and calcium (TRP) channels that can be activated by a variety of stimuli including chemical odorants. We identified 29 annotated PPK genes out of 37 previously annotated PPKs (Matthews et al., 2016; Hill et al., 2021) in our RNA-seq data, only one of which [*ppk14009* (AAEL014009)] was significantly differentially expressed in PR relative to the other strains (Supplementary File 8). Fifteen TRP channels have been previously annotated (Matthews et al., 2016). We observed all 15 TRPs and one transcript (AAEL021199) putatively identified as a TRP in our dataset (Supplementary File 3). None of these TRP channels were significantly different between any of the four strains.

### Transcripts With High Expression in Puerto Rico

**FIGURE 4:**
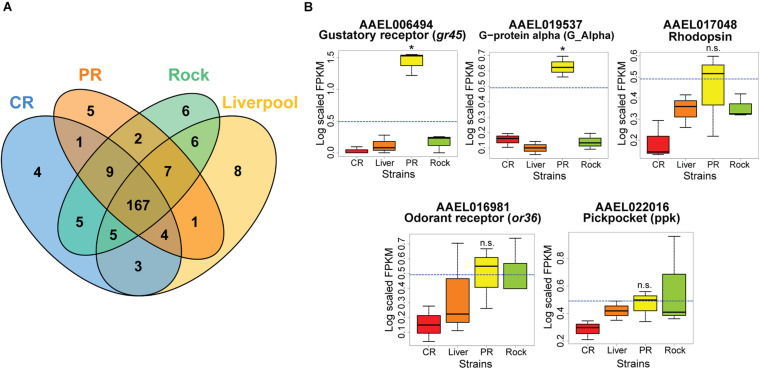
Olfactory related genes highly expressed in PR mosquitoes. **(A)** Venn diagram of olfactory genes in each strain with expression greater than the log scaled median olfactory related gene expression (0.4909181 FPKM). Five genes were identified as having higher median expression in PR mosquitoes relative to the median expression of all olfactory genes. **(B)** Boxplots of the normalized FPKM values of the five overexpressed genes in PR mosquitoes. The dashed blue line represents the median log scaled FPKM value of all olfactory related genes. Genes with significantly increased expression (determined by Kruskal–Wallis tests, *p* < 0.05) in PR relative to the other three strains are marked with asterisks, and non-significant genes are marked (n.s.).

We next wanted to identify which genes in our dataset had very high expression in each strain. To determine this, we calculated the median FPKM value of all odor-associated genes, and then selected all genes with a median expression value above this level in at least one strain. We visualized this data in a Venn diagram, and observed that each strain had a small sub-set of genes whose median expression in that strain was greater than the median expression of all olfaction-associated genes (Figure 4A). We identified five olfaction-associated genes in PR with increased expression when compared to the expression levels of all olfactory genes in all strains (Figure 4A). Interestingly, one of these highly expressed genes was an odorant receptor (*or 36*, AAEL016981) and one was a gustatory receptor (*gr45*, AAEL006494), which was significantly higher in PR mosquitoes relative to the other strains. We visualized the expression patterns of the five genes with high expression in PR in Figure 4B.

### qRT-PCR Validation

We selected three olfaction related genes to validate the expression profiles observed in our RNA-seq dataset. We performed qRT-PCR on three olfaction related genes (*or26*, *or97*, and *obp56a*) and two candidate reference genes (*rps7* and β-*actin*). We used the RefFinder tool (Xie et al., 2012) to identify the most stably expressed gene and determined that *rps7* was the most appropriate reference gene for normalization of our olfaction related gene data (Supplementary File 9A).

We calculated relative mRNA levels of three olfaction related genes to validate their expression patterns in relation to the expression patterns we observed in our RNA-seq data. We observed no significant difference in *or26* gene expression between strains (Supplementary File 9B, top). However, besides high variation within samples, mean *or26* mRNA levels were highest in PR followed by Rock, which is consistent with the observed trend in our RNA-seq data (Supplementary File 9B, top). PR and Rock *or97* relative mRNA levels were significantly greater than Liverpool *or97* (*p* < 0.05) (Supplementary File 9B, middle). Additionally, Liverpool *or97* relative mRNA levels were lower than CR *or97*, but not significantly so (Supplementary File 9B, middle). These trends in relative *or97* mRNA levels also follow the trends observed in our RNA-seq data, with Liverpool *or97* being the lowest expressed among the four strains, and PR and Rock having similar *or97* expression (Supplementary File 9B, middle). Finally, relative PR mRNA levels of *obp56a* were significantly greater than all other strains (*p* < 0.05), and relative CR mRNA levels were significantly lower than all other strains (*p* < 0.05) (Supplementary File 9B, bottom). These trends again match the observed trend in our RNA-seq data of *obp56a* expression (Supplementary File 9B, bottom).

## Discussion

The yellow fever mosquito, *Aedes aegypti*, is a principal vector of several important arboviral diseases that cause widespread human morbidity and mortality in its distribution range (Marquardt, 2004; World Health Organization, 2016). Disease transmission by mosquitoes is tightly connected with the acquisition of vertebrate blood by female mosquitoes to gain nutrients for egg development (Clements, 1992). Thus, it is important to understand the physiology behind how *Ae. aegypti* females sense and respond to olfactory cues from their human hosts. Many different laboratory strains are available for use in behavioral and molecular experiments, so it is important to determine how applicable host seeking and gene expression results from one strain are to other strains, and the species as a whole.

In this study, we report the attraction of seven strains of *Ae. aegypti* to a human host, and the associated olfactory gene expression profile from antennae of four of these strains. Our comparison of average attraction rates in seven different strains of *Ae. aegypti* via Y-tube olfactometer showed significantly reduced attraction rates in mosquitoes from the insecticide-resistant *Ae. aegypti* Puerto Rico strain. All other strains UGAL, Liverpool, Rockefeller, Costa Rica, Orco2, and Orco16 showed similar, high attraction rates toward humans (Figure 1). The attraction behavior of the Orco mutant strains was surprising as mutations in the *orco* gene have been reported to decrease attraction to human odor and repellency response to aerosolized DEET (DeGennaro et al., 2013). In the previous study, the loss of attraction to human odors in *orco* mutant mosquitoes was alleviated by performing the attraction assay in the presence of CO_2_ (DeGennaro et al., 2013). As our Y-tube bioassay measures attraction to a live human host, the Orco strains used in our study were exposed to human odors, CO_2_, and body heat which may explain the equivalent attraction rates of Orco mutant strains with aggressive strains such as UGAL, Rock, and Liverpool. Even more surprising was the reduction in host attraction of Orco strains in the presence of DEET. Previously, Orco mutants have been reported to have reduced repellency to aerosolized DEET (DeGennaro et al., 2013). However, our results showed a significant reduction in attraction of Orco2 and Orco16 strains to a human host in the presence of DEET (Figures 1B,C). We are not entirely sure why these strains had a significant response to DEET, but it may be due to differences in the mutants reported in our paper and those reported by DeGennaro et al. (2013). In the previous study, heteroallelic strains of *orco*^2/5^ or *orco*^16/5^ were reported to have reduced repellency to DEET (DeGennaro et al., 2013). However, we used homoallelic *orco*^2/2^ (Orco2 strain) and *orco*^16/16^ (Orco16 strain) mutants, so it is possible that a synergistic effect with the *orco*^5^ allele is necessary for reduced DEET repellency, which we would not see with our strains.

We also performed RNA-seq analysis of the antennae transcriptomes of four different strains of *Aedes aegypti*: PR, CR, Rock, and Liverpool. We selected these four strains as they represent commonly used laboratory strains in experiments with wild type (CR, Liverpool, Rock), and insecticide-resistant (PR) *Ae. aegypti*. PCA analysis of transcriptomes from the four strains revealed that all four strains clustered separately (Figure 2) indicating that the gene expression profiles of these strains are different despite the similarity in human attraction of CR, Liverpool, and Rock strains. We detected a large number of olfaction-associated genes, including ORs, OBPs, IRs, GRs, PPKs, and TRP channels in all four strains (Figure 3 and Supplementary File 3). We observed slightly higher overall expression of ORCO in Rock compared to the other three strains (Figure 3) which follows the trend of non-significantly increased percent attraction in Rock relative to the other two wild type strains (Figure 1) as ORCO is an obligate co-receptor necessary for OR function. We did not observe significant differences in expression of the seven ORs previously determined to sense human odor compounds (Bohbot and Dickens, 2010; Bohbot et al., 2011; McBride et al., 2014; Bohbot and Pitts, 2015; Ruel et al., 2019; Zeng et al., 2019). However, we did observe significant differences in expression of several ORs between the four strains analyzed. This includes significantly higher expression of *or103* (AAEL017505) in PR mosquitoes. While the ligand binding profile of *or103* has not been determined in *Ae. aegypti*, this OR has been previously shown to be highly expressed in a strain of human-preferring *Ae. aegypti* in Kenya (McBride et al., 2014). Wang et al. (2010) identified large amounts of overlap in interactions between certain ligands and different ORs in the related mosquito, *Anopheles gambiae*. So it is possible that while we do see some variability in antennal gene expression, the relative attraction of these strains with the exception of PR to a human host may be explained by functional redundancy between differentially expressed ORs to the same set of ligands. The set of odorant ligands of *Ae. aegypti* ORs is not as well classified as other species, particularly *Anopheles gambiae* (Carey et al., 2010; Wang et al., 2010). Thus, future studies to determine the full suite of human-sensing ORs in *Ae. aegypti* are necessary to further our understanding of what ORs odorant ligands are most important for host-seeking and how much redundancy is encoded into the odor sensing repertoire of this species.

In addition to ORs, other receptor classes including GRs and IRs have been implicated to play important roles in odor sensation. One IR in particular, *ir8a* (AAEL002922), is an IR co-receptor necessary for sensation of lactic acid in *Ae. aegypti* (Raji et al., 2019). We identified this IR in our transcriptome assay, and while its expression varied between strains ranging from lowest expression in CR to highest in Rock (Supplementary Files 3, 4), these differences were not significant. As *ir8a* is a co-receptor for other IRs, it is possible that these non-significant differences in its expression may alter the function of other IRs and could explain some of the non-significant differences in percent attraction observed in our Y-tube bioassay (Figure 1). Additionally, in *An. gambiae*, another antennal IR, *ir75k.1* (AAEL014809), whose ortholog was annotated in our study has been demonstrated to respond to C7-C9 straight chain carboxylic acids (Pitts et al., 2017) which are components of human sweat (Cork and Park, 1996). This IR was also elevated in our Rock samples relative to the other three strains. This non-significant elevation of *ir75k.1* in Rock may also contribute to the observed differences in attraction between different strains (Figure 1). Three GRs, *gr1* (AAEL002380), *gr2* (AAEL002167), and *gr3* (AAEL010058) form complexes necessary for CO_2_ sensation in mosquitoes (Erdelyan et al., 2012; Ray, 2015; Fleischer et al., 2018; Kumar et al., 2020). We identified all three of these GRs in our antennal transcriptome data (Supplementary File 3). Interestingly, *gr2* (AAEL002167) expression was significantly higher in CR mosquitoes relative to the other strains (Supplementary File 7). This increase in *gr2* expression was not associated with an increase in percent attraction in CR relative to other strains, but *gr2* knockdowns have been previously reported to not affect CO_2_ sensation in *Ae. aegypti* (Erdelyan et al., 2012), so it is possible that increased levels of *gr2* alone is not enough to increase host attraction. Also, GRs are more prominently expressed in the maxillary palps of mosquitoes (Lu et al., 2007; Syed and Leal, 2007), so it is more likely that the observed increase in *gr2* expression in CR without increased host attraction is due to higher levels of GR expression, particularly of the CO_2_-sensing GRs in the maxillary palps of all strains.

Chemoreception in insects occurs in several different structures including antennae, mouthparts, wing fringes, and tarsal segments (Vosshall and Stocker, 2007). For example, GRs are receptors generally localized in sensilla on other sensory regions than antennae such as the tarsal segments and mouthparts (Dunipace et al., 2001; Scott et al., 2001; Thorne et al., 2004; Wang et al., 2004; Dahanukar et al., 2007; Sparks et al., 2013, 2014). Additionally, ORs, IRs, and OBPs are differentially expressed both between males and females, and across different life stages, sensory structures and during female reproductive cycles (Bohbot et al., 2007; Matthews et al., 2016; Fleischer et al., 2018; Tallon et al., 2019; Hill et al., 2021). Therefore, we were not surprised to find a limited complement of each chemoreceptor class as we only analyzed antennae from non-blood fed adult (5-days post emergence) females. Of interest, we did identify several sensory receptors with expression previously classified to be specific to the maxillary palps, including the major CO_2_-sensing GRs *gr1* (AAEL002380), *gr2* (AAEL002167), and *gr3* (AAEL010058) (Erdelyan et al., 2012; Fleischer et al., 2018), as well as *or8* (AAEL012254) (Bohbot et al., 2007). We do not believe our detection of these transcripts is due to cross-contamination of our antennal samples with maxillary palp tissues, as the detected expression of these genes across all samples is low (<2 FPKM) (Supplementary File 3). We also identified several larval-specific ORs, [*or9* (AAEL006005), *or14* (AAEL008442), *or34* (AAEL003395), *or40* (AAEL005767), *or48* (AAEL020825), and *or58* (AAEL006202)] (Bohbot et al., 2007) with low (<1 FPKM) expression in many of our samples (Supplementary File 3). We are certain there was no cross-contamination between life stages in our samples, so this is likely an example of “leaky” transcription of these genes which we detected due to the sequencing depth of our samples. This also provides support for our identification of maxillary palp-specific genes in our dataset being due to a basal level of gene expression rather than tissue contamination. Thus, while we included these transcripts in our findings, we do not believe that they represent a significant contamination of our samples, nor do they have any effect on our conclusions. Finally, we analyzed strains that have been reared under standardized laboratory conditions for generations so it is possible that over the generations of breeding under laboratory conditions, the numbers of receptors necessary for these mosquitoes to identify meals, hosts, and egg-laying sites has changed, and the gene expression pattern has also changed in response, leading to a lower variation in overall receptor expression. Therefore it would be interesting to perform a similar study with wild strains from different locations in the future.

Principal component analysis of the four strains indicated that differential gene expression profiles exist between the four strains in our transcriptome study (Figure 2). Despite the differences in overall gene expression, we did not observe significant differences in olfaction-associated genes with known human-sensing capability between any of the four strains with the exception of *gr2* (AAEL002167) which was expressed at low levels in all tissues, but was significantly higher levels in CR mosquitoes (Supplementary File 7). In addition to this general pattern of olfactory gene expression, we only observed significant changes in one *Ae. aegypti* OR previously implicated with human attraction, *or103* (AAEL017505) (McBride et al., 2014). Interestingly, this OR was expressed at significantly greater levels in PR mosquitoes relative to the other three strains (Supplementary File 8). This pattern was unexpected given the behavior of these mosquito strains in our Y-tube olfaction bioassay (Figure 1). We propose several possible explanations for this. First, the ligands of many *Ae. aegypti* odorant receptors are not classified. Thus, it is possible that while the expression of most olfactory related genes is similar between PR and other strains, certain ORs or OBPs with unknown human odor ligands may be differentially expressed in PR. Second, it is possible that sequence variations or splice variations that change the affinity and/or activity of ORs or OBPs are found in PR mosquitoes but not in other strains. These alterations in mRNA sequence may explain the difference in host seeking behavior between strains even though gene expression patterns are similar among these strains. Third, it is possible that while antennal gene expression of human-associated olfactory genes is similar between PR and the other strains, expression of human-sensing genes differs between PR and the other strains in other sensory structures, such as the maxillary palps which were not assayed in this study. Finally, we have previously profiled the sequence of the sodium voltage gated channel (*para*) in PR and Rock mosquitoes (Pinch et al., 2020) and identified multiple known knockdown resistance (*kdr*) mutations in the PR *para* gene. These *kdr* mutations alter the activity of *para* channels, so it is possible that these mutations present in PR mosquitoes disrupt signal propagation along olfactory neurons. We hypothesize that this represents an evolutionary cost of pyrethroid resistance whereby altered signal propagation negatively affects host odor perception and therefore host seeking in PR mosquitoes, even with minimal alterations in expression of human odor sensation genes.

These experiments provide a comprehensive overview of host attraction and olfactory gene expression in the antennae of several common laboratory strains of *Ae. aegypti*. We observed different expression profiles of many olfaction-related genes between Liverpool, Rock, CR, and PR females, with all four strains showing distinct gene expression profiles (Figure 2). Interestingly, despite these differences in gene expression between strains (Figure 2), only PR mosquitoes exhibited a different behavioral response to a human host (Figures 1B,C). Taken together, these results demonstrate that odor detection and host sensing behavior share a complex interaction with built-in redundancy for sensing different combinations of odor molecules, and likely interactions with other signal sensation and propagation mechanisms. This study highlights the importance of using standardized strains to minimize variability, and the necessity of accurately reporting which strains are used in experiments so that conclusions about phenotype can be placed in the correct genotypic context.

## Data Availability Statement

The data presented in the study are deposited in the NCBI BioProject repository, accession number PRJNA715771.

## Author Contributions

SM cultured mosquito strains, performed Y-tube assays, performed dissections of antennae and pedicels, performed qRT-PCR, generated figures and tables, and wrote the manuscript. MP cleaned-up RNA for Illumina RNA-seq, performed qRT-PCR, generated figures and tables, and wrote the manuscript. YK performed Y-tube assays, performed statistical analysis on behavioral data, and generated figures. YL performed statistical analysis of Illumina RNA-seq data and generated figures. SR cultured mosquito strains, extracted RNA for Illumina RNA-seq, and generated figures. IH conceived of the experiments. All authors contributed to the article and approved the submitted version.

## Conflict of Interest

The authors declare that the research was conducted in the absence of any commercial or financial relationships that could be construed as a potential conflict of interest.

## Publisher’s Note

All claims expressed in this article are solely those of the authors and do not necessarily represent those of their affiliated organizations, or those of the publisher, the editors and the reviewers. Any product that may be evaluated in this article, or claim that may be made by its manufacturer, is not guaranteed or endorsed by the publisher.

## Footnotes

1
vectorbase.org

2
https://www.heartcure.com.au/reffinder/

3
http://ewindup.info/miner/

## References

[B1] Acree F. Jr. Turner R. B. Gouck H. K. Beroza M. Smith N. (1968). L-Lactic acid: a mosquito attractant isolated from humans. *Science* 161 1346–1347. 10.1126/science.161.3848.1346 5673445

[B2] Andersen C. L. Jensen J. L. Orntoft T. F. (2004). Normalization of real-time quantitative reverse transcription-PCR data: a model-based variance estimation approach to identify genes suited for normalization, applied to bladder and colon cancer data sets. *Cancer Res.* 64 5245–5250. 10.1158/0008-5472.can-04-0496 15289330

[B3] Biessmann H. Andronopoulou E. Biessmann M. R. Douris V. Dimitratos S. D. Eliopoulos E. (2010). The Anopheles gambiae odorant binding protein 1 (AgamOBP1) mediates indole recognition in the antennae of female mosquitoes. *PLoS One* 5:e9471. 10.1371/journal.pone.0009471 20208991 PMC2830424

[B4] Bohbot J. D. Dickens J. C. (2009). Characterization of an enantioselective odorant receptor in the yellow fever mosquito *Aedes aegypti*. *PLoS One* 4:e7032. 10.1371/journal.pone.0007032 19753115 PMC2737144

[B5] Bohbot J. D. Dickens J. C. (2010). Insect repellents: modulators of mosquito odorant receptor activity. *PLoS One* 5:e12138. 10.1371/journal.pone.0012138 20725637 PMC2920324

[B6] Bohbot J. D. Pitts R. J. (2015). The narrowing olfactory landscape of insect odorant receptors. *Front. Ecol. Evol.* 3:39. 10.3389/fevo.2015.00039

[B7] Bohbot J. D. Jones P. L. Wang G. Pitts R. J. Pask G. M. Zwiebel L. J. (2011). Conservation of indole responsive odorant receptors in mosquitoes reveals an ancient olfactory trait. *Chem. Senses.* 36 149–160. 10.1093/chemse/bjq105 20956733 PMC3020388

[B8] Bohbot J. D. Sparks J. T. Dickens J. C. (2014). The maxillary palp of *Aedes aegypti*, a model of multisensory integration. *Insect Biochem. Mol. Biol.* 48 29–39. 10.1016/j.ibmb.2014.02.007 24613607

[B9] Bohbot J. Pitts R. J. Kwon H. W. Rutzler M. Robertson H. M. Zwiebel L. J. (2007). Molecular characterization of the *Aedes aegypti* odorant receptor gene family. *Insect Mol Biol.* 16 525–537. 17635615 10.1111/j.1365-2583.2007.00748.x PMC3100214

[B10] Bolger A. M. Lohse M. Usadel B. (2014). Trimmomatic: a flexible trimmer for Illumina sequence data. *Bioinformatics* 30 2114–2120. 10.1093/bioinformatics/btu170 24695404 PMC4103590

[B11] Brito N. F. Moreira M. F. Melo A. C. (2016). A look inside odorant-binding proteins in insect chemoreception. *J. Insect Physiol.* 95 51–65. 10.1016/j.jinsphys.2016.09.008 27639942

[B12] Carey A. F. Carlson J. R. (2011). Insect olfaction from model systems to disease control. *Proc. Natl. Acad. Sci. U.S.A.* 108 12987–12995. 10.1073/pnas.1103472108 21746926 PMC3156210

[B13] Carey A. F. Wang G. Su C. Y. Zwiebel L. J. Carlson J. R. (2010). Odorant reception in the malaria mosquito Anopheles gambiae. *Nature* 464 66–71. 10.1038/nature08834 20130575 PMC2833235

[B14] Carlson D. A. Smith N. Gouck H. K. Godwin D. R. (1973). Yellowfever mosquitoes: compounds related to lactic acid that attract females. *J. Econ. Entomol.* 66 329–331. 10.1093/jee/66.2.329

[B15] Clements A. N. (1992). *The Biology of Mosquitoes.* London: Chapman & Hall.

[B16] Colomb J. Brembs B. (2014). Sub-strains of *Drosophila* Canton-S differ markedly in their locomotor behavior. *F1000Res.* 3:176. 10.12688/f1000research.4263.2 25210619 PMC4156027

[B17] Cork A. Park K. C. (1996). Identification of *electrophysiologically*-active compounds for the malaria mosquito, Anopheles gambiae, in human sweat extracts. *Med. Vet. Entomol.* 10 269–276. 10.1111/j.1365-2915.1996.tb00742.x 8887339

[B18] Costa-da-Silva A. L. Ioshino R. S. Araújo H. R. Kojin B. B. Zanotto P. M. Oliveira D. B. L. (2017). Laboratory strains of *Aedes aegypti* are competent to Brazilian Zika virus. *PLoS One* 12:e0171951. 10.1371/journal.pone.0171951 28187183 PMC5302382

[B19] Dahanukar A. Lei Y.-T. Kwon J. Y. Carlson J. R. (2007). Two Gr genes underlie sugar reception in *Drosophila*. *Neuron* 58 503–516. 10.1016/j.neuron.2007.10.024 17988633 PMC2096712

[B20] DeGennaro M. McBride C. S. Seeholzer L. Nakagawa T. Dennis E. J. Goldman C. (2013). orco mutant mosquitoes lose strong preference for humans and are not repelled by volatile DEET. *Nature* 498 487–491. 10.1038/nature12206 23719379 PMC3696029

[B21] Dobin A. Davis C. A. Schlesinger F. Drenkow J. Zaleski C. Jha S. (2013). STAR: ultrafast universal RNA-seq aligner. *Bioinformatics* 29 15–21. 10.1093/bioinformatics/bts635 23104886 PMC3530905

[B22] Dunipace L. Meister S. McNealy C. Amrein H. (2001). Spatially restricted expression of candidate taste receptors in the *Drosophila* gustatory system. *Curr. Biol.* 11 822–835. 10.1016/s0960-9822(01)00258-5 11516643

[B23] Erdelyan C. N. Mahood T. H. Bader T. S. Whyard S. (2012). Functional validation of the carbon dioxide receptor genes in *Aedes aegypti* mosquitoes using RNA interference. *Insect Mol. Biol.* 21 119–127. 10.1111/j.1365-2583.2011.01120.x 22122783

[B24] Fleischer J. Krieger J. (2018). Insect pheromone receptors - key elements in sensing intraspecific chemical signals. *Front. Cell Neurosci.* 12:425. 10.3389/fncel.2018.00425 30515079 PMC6255830

[B25] Fleischer J. Pregitzer P. Breer H. Krieger J. (2018). Access to the odor world: olfactory receptors and their role for signal transduction in insects. *Cell. Mol. Life Sci.* 75 485–508. 10.1007/s00018-017-2627-5 28828501 PMC11105692

[B26] Geier M. Boeckh J. (2003). A new Y-tube olfactometer for mosquitoes to measure the attractiveness of host odours. *Entomol. Exp. Appl.* 92 9–19. 10.1046/j.1570-7458.1999.00519.x

[B27] Gloria-Soria A. Soghigian J. Kellner D. Powell J. R. (2019). Genetic diversity of laboratory strains and implications for research: the case of *Aedes aegypti*. *PLoS Negl. Trop. Dis.* 13:e0007930. 10.1371/journal.pntd.0007930 31815934 PMC6922456

[B28] Gómez A. Seccacini E. Zerba E. Licastro S. (2011). Comparison of the insecticide susceptibilities of laboratory strains of *Aedes aegypti* and *Aedes albopictus*. *Mem. Inst. Oswaldo Cruz.* 106 993–996. 10.1590/s0074-02762011000800015 22241122

[B29] Hill S. R. Taparia T. Ignell R. (2021). Regulation of the antennal transcriptome of the dengue vector, *Aedes aegypti* during the first gonotrophic cycle. *BMC Genomics.* 22:71. 10.1186/s12864-020-07336-w 33478394 PMC7821643

[B30] Kumar A. Tauxe G. M. Perry S. Scott C. A. Dahanukar A. Ray A. (2020). Contributions of the conserved insect carbon dioxide receptor subunits to odor detection. *Cell Rep.* 31:107510. 10.1016/j.celrep.2020.03.074 32294446 PMC7552916

[B31] Kuno G. (2014). Early history of laboratory breeding of *Aedes aegypti* (Diptera: Culicidae) focusing on the origins and use of selected strains. *J. Med. Entomol.* 47 957–971. 10.1603/me10152 21175042

[B32] Leal G. M. Leal W. S. (2015). Binding of a fluorescene reporter and a ligand to an odorant-binding protein of the yellow fever mosquito, *Aedes aegypti* [version 2; peer review: 2 approved]. *F1000 Res.* 3:305. 10.12688/f1000research.5879.2 25671088 PMC4309172

[B33] Leal W. S. (2013). Odorant reception in insects: roles of receptors, binding proteins, and degrading enzymes. *Annu. Rev. Entomol.* 58 373–391. 10.1146/annurev-ento-120811-153635 23020622

[B34] Liao Y. Smyth G. K. Shi W. (2013). The Subread aligner: fast, accurate and scalable read mapping by seed-and-vote. *Nucleic Acids Res.* 41:e108. 10.1093/nar/gkt214 23558742 PMC3664803

[B35] Liao Y. Smyth G. K. Shi W. (2014). featureCounts: an efficient general purpose program for assigning sequence reads to genomic features. *Bioinformatics* 30 923–930. 10.1093/bioinformatics/btt656 24227677

[B36] Lu T. Qiu Y. T. Wang G. Kwon J. Y. Rutzler M. Kwon H.-W. (2007). Odor coding in the maxillary palp of the malaria vector mosquito Anopheles gambiae. *Curr. Biol.* 17 1533–1544. 10.1016/j.cub.2007.07.062 17764944 PMC3113458

[B37] Manoharan M. Ng Fuk Chong M. Vaïtinadapoulé A. Frumence E. Sowdhamini R. Offmann B. (2013). Comparative genomics of odorant binding proteins in Anopheles gambiae, *Aedes aegypti*, and Culex quinquefasciatus. *Genome Biol. Evol.* 5 163–180. 10.1093/gbe/evs131 23292137 PMC3595023

[B38] Marquardt W. H. (2004). *Biology of Disease Vectors*, 2nd Edn. Cambridge, MA: Academic Press.

[B39] Matthews B. J. Dudchenko O. Kingan S. B. Koren S. Antoshechkin I. Crawford J. E. (2018). Improved reference genome of *Aedes aegypti* informs arbovirus vector control. *Nature* 563 501–507. 30429615 10.1038/s41586-018-0692-z PMC6421076

[B40] Matthews B. J. McBride C. S. DeGennaro M. Despo O. Vosshall L. B. (2016). The neurotranscriptome of the *Aedes aegypti* mosquito. *BMC Genomics* 17:32. 10.1186/s12864-015-2239-0 26738925 PMC4704297

[B41] McBride C. S. Baier F. Omondi A. B. Spitzer S. A. Lutomiah J. Sang R. (2014). Evolution of mosquito preference for humans linked to an odorant receptor. *Nature* 515 222–227. 10.1038/nature13964 25391959 PMC4286346

[B42] Melo A. C. A. Rützler M. Pitts R. J. Zwiebel L. J. (2004). Identification of a chemosensory receptor from the yellow fever mosquito, *Aedes aegypti*, that is highly conserved and expressed in olfactory and gustatory organs. *Chem. Senses.* 29 403–410. 10.1093/chemse/bjh041 15201207

[B43] Molestina R. E. (2010). BEI Resources: a biological resource center for parasitologists. *Trends Parasitol.* 26:559. 10.1016/j.pt.2010.09.003 20932801 PMC2980584

[B44] Pelosi P. Iovinella I. Zhu J. Wang G. Dani F. R. (2018). Access to the odor world: olfactory receptors and their role for signal transduction in insects. *Cell. Mol. Life Sci.* 75 485–508. 28828501 10.1007/s00018-017-2627-5 PMC11105692

[B45] Pfaffl M. W. Tichopad A. Prgomet C. Neuvians T. P. (2004). Determination of stable housekeeping genes, differentially regulated target genes and sample integrity: bestkeeper–Excel-based tool using pair-wise correlations. *Biotechnol. Lett.* 26 509–515. 10.1023/b:bile.0000019559.84305.47 15127793

[B46] Pinch M. Rodriguez S. D. Mitra S. Kandel Y. Moore E. Hansen I. A. (2020). Low levels of Pyrethroid resistance in hybrid offspring of a highly resistant and a more susceptible mosquito strain. *J. Insect. Sci.* 20:1. 10.1093/jisesa/ieaa060 32610346 PMC7329315

[B47] Pitts R. J. Derryberry S. L. Zhang Z. Zwiebel L. J. (2017). Variant ionotropic receptors in the malaria vector mosquito Anopheles gambiae tuned to amines and carboxylic acids. *Sci. Rep.* 7:40297. 28067294 10.1038/srep40297 PMC5220300

[B48] Raji J. I. Melo N. Castillo J. S. Gonzalez S. Saldana V. Stensmyr M. C. (2019). *Aedes aegypti* mosquitoes detect acidic volatiles found in human odor using the IR8a pathway. *Curr. Biol.* 29 1253–1262e1257. 30930038 10.1016/j.cub.2019.02.045 PMC6482070

[B49] Ray A. (2015). Reception of odors and repellents in mosquitoes. *Curr. Opin. Neurobiol.* 34 158–164. 10.1016/j.conb.2015.06.014 26202080 PMC4577459

[B50] Rodriguez S. D. Drake L. L. Price D. P. Hammond J. I. Hansen I. A. (2015). The Efficacy of Some Commercially Available Insect Repellents for Aedes aegypti (Diptera: Culicidae) and *Aedes albopictus* (Diptera: Culicidae). *J. Insect Sci.* 15:140. 10.1093/jisesa/iev125 26443777 PMC4667684

[B51] Roth L. M. (1951). Loci of sensory end-organs used by mosquitoes (*Aedes aegypti* (L.) and Anopheles quadrimaculatus Say) in receiving host stimuli. *Annal. Entomol. Soc. Am.* 44 59–74. 10.1093/aesa/44.1.59

[B52] Ruel D. M. Yakir E. Bohbot J. D. (2019). Supersensitive odorant receptor underscores pleiotropic roles of indoles in mosquito ecology. *Front. Cell. Neurosci.* 12:533. 10.3389/fncel.2018.00533 30733668 PMC6353850

[B53] Scott K. Brady R. Jr. Cravchik A. Morozov P. Rzhetsky A. Zuker C. (2001). A chemosensory gene family encoding candidate gustatory and olfactory receptors in *Drosophila*. *Cell* 104 661–673. 10.1016/s0092-8674(01)00263-x 11257221

[B54] Silver N. Best S. Jiang J. Thein S. L. (2006). Selection of housekeeping genes for gene expression studies in human reticulocytes using real-time PCR. *BMC Mol. Biol.* 7:33. 17026756 10.1186/1471-2199-7-33 PMC1609175

[B55] Song M. Zhong H. (2020). Efficient weighted univariate clustering maps outstanding dysregulated genomic zones in human cancers. *Bioinformatics* 36 5027–5036. 10.1093/bioinformatics/btaa613 32619008 PMC7755420

[B56] Sparks J. T. Bohbot J. D. Dickens J. C. (2014). The genetics of chemoreception in the labella and tarsi of *Aedes aegypti*. *Insect Biochem. Mol. Biol.* 48 8–16. 10.1016/j.ibmb.2014.02.004 24582661

[B57] Sparks J. T. Vinyard B. T. Dickens J. C. (2013). Gustatory receptor expression in the labella and tarsi of *Aedes aegypti*. *Insect Biochem. Mol. Biol.* 43 1161–1171. 10.1016/j.ibmb.2013.10.005 24157615

[B58] Syed Z. Leal W. S. (2007). Maxillary palps are broad spectrum odorant detectors in *Culex quinquefasciatus*. *Chem. Senses.* 32 727–738. 10.1093/chemse/bjm040 17569743

[B59] Takken W. Knols B. G. (1999). Odor-mediated behavior of *Afrotropical* malaria mosquitoes. *Annu. Rev. Entomol.* 44 131–157. 10.1146/annurev.ento.44.1.131 9990718

[B60] Tallon A. K. Hill S. R. Ignell R. (2019). Sex and age modulate antennal chemosensory-related genes linked to the onset of host seeking in the yellow-fever mosquito, *Aedes aegypti*. *Sci. Rep.* 9:43. 30631085 10.1038/s41598-018-36550-6 PMC6328577

[B61] Thorne N. Chromey C. Bray S. Amrein H. (2004). Taste perception and coding in *Drosophila*. *Curr. Biol.* 14 1065–1079. 10.1016/j.cub.2004.05.019 15202999

[B62] Vandesompele J. De Preter K. Pattyn F. Poppe B. Van Roy N. De Paepe A. (2002). Accurate normalization of real-time quantitative RT-PCR data by geometric averaging of multiple internal control genes. *Genome Biol.* 3:RESEARCH0034. 12184808 10.1186/gb-2002-3-7-research0034 PMC126239

[B63] Venthur H. Zhou J. J. (2018). Odorant receptors and odorant-binding proteins as insect pest control targets: a comparative analysis. *Front. Physiol.* 9:1163. 10.3389/fphys.2018.01163 30197600 PMC6117247

[B64] Vosshall L. B. Hansson B. S. (2011). A unified nomenclature system for the insect olfactory coreceptor. *Chem. Senses* 36 497–498. 10.1093/chemse/bjr022 21441366

[B65] Vosshall L. B. Stocker R. F. (2007). Molecular architecture of smell and taste in *Drosophila*. *Annu. Rev. Neurosci.* 30 505–533. 17506643 10.1146/annurev.neuro.30.051606.094306

[B66] Wang G. Carey A. F. Carlson J. R. Zwiebel L. J. (2010). Molecular basis of odor coding in the malaria vector mosquito Anopheles gambiae. *Proc. Natl. Acad. Sci. U.S.A.* 109 4418–4423. 10.1073/pnas.0913392107 20160092 PMC2840125

[B67] Wang H. Song M. (2011). Ckmeans.1d.dp: optimal k-means clustering in one dimension by dynamic programming. *R J.* 3 29–33. 10.32614/rj-2011-015 27942416 PMC5148156

[B68] Wang J. Murphy E. J. Nix J. C. Jones D. N. (2020). *Aedes aegypti* odorant binding protein 22 selectively binds fatty acids through a conformational change in its C-terminal tail. *Sci. Rep.* 10:3300. 32094450 10.1038/s41598-020-60242-9 PMC7039890

[B69] Wang Z. Singhvi A. Kong P. Scott K. (2004). Taste representations in the *Drosophila* brain. *Cell.* 117 981–991. 10.1016/j.cell.2004.06.011 15210117

[B70] Wicher D. (2018). Tuning insect odorant receptors. *Front. Cell. Neurosci.* 12:94. 10.3389/fncel.2018.00094 29674957 PMC5895647

[B71] World Health Organization (2016). *Dengue and Severe Dengue.* Available online at: https://www.who.int/news-room/fact-sheets/detail/dengue-and-severe-dengue (accessed September 20, 2019).

[B72] Xie F. Xiao P. Chen D. Xu L. Zhang B. (2012). miRDeepFinder: a miRNA analysis tool for deep sequencing of plant small RNAs. *Plant Mol. Biol.* 10.1007/s11103-012-9885-2 [Epub ahead of print]. 22290409

[B73] Ye J. Coulouris G. Zaretskaya I. Cutcutache I. Rozen S. Madden T. L. (2012). Primer-BLAST: a tool to design target-specific primers for polymerase chain reaction. *BMC Bioinformatics* 13:134. 10.1186/1471-2105-13-134 22708584 PMC3412702

[B74] Zarubin M. Yakhnenko A. Kravchenko E. (2020). Transcriptome analysis of Drosophila melanogaster laboratory strains of different geographical origin after long-term laboratory maintenance. *Ecol. Evol.* 10 7082–7093. 10.1002/ece3.6410 32760513 PMC7391317

[B75] Zeng F. Xu P. Leal W. S. (2019). Odorant receptors from *Culex quinquefasciatus* and *Aedes aegypti* sensitive to floral compounds. *Insect Biochem. Mol. Biol.* 113:103213. 10.1016/j.ibmb.2019.103213 31442487 PMC6733653

[B76] Zhao S. Fernald R. D. (2005). Comprehensive algorithm for quantitative real-time polymerase chain reaction. *J. Comput. Biol.* 12 1047–1064. 10.1089/cmb.2005.12.1047 16241897 PMC2716216

