# Supplementary material for: Olfaction-Related Gene Expression in the Antennae of Female Mosquitoes From Common Aedes aegypti Laboratory Strains
Source: Front Physiol. 2021 Aug 23;12:668236. doi: 10.3389/fphys.2021.668236 (PMC8419471; doi:10.3389/fphys.2021.668236)
Supplement: Supplementary file 1 [file Table_1.DOCX]

**Supplemental File 1. qRT-PCR primers used in this study.** *Candidate internal reference genes.

| **Gene** | **Primer** | **Sequence** | **Annealing Temp. (°C)** |
| --- | --- | --- | --- |
| *or26* | For | AGTGTGCTTCAGAACTTCTCGTCG | 60 |
|  | Rev | ACTCGCATCCATCAACTCGGTTCC |  |
| *or97* | For | CGCAAAAATAGGACCGTCACCG | 60 |
|  | Rev | CGAGGCACATAACAGAGCATTGG |  |
| *obp56a* | For | GGTTGTTTTTCTTACCGTGGTTGC | 60 |
|  | Rev | TGCTTTCCTCCAAAATTGACCCC |  |
| *β-actin** | For | GACTACCTGATGAAGATCCTGAC | 60 |
|  | Rev | GCACAGCTTCTCCTTAATGTCAC |  |
| *rps7** | For | TCAGTGTACAAGAAGCTGACCGGA | 60 |
|  | Rev | TTCCGCGCGCGCTCACTTATTAGATT |  |
