# Supplementary material for: Olfaction-Related Gene Expression in the Antennae of Female Mosquitoes From Common Aedes aegypti Laboratory Strains
Source: Front Physiol. 2021 Aug 23;12:668236. doi: 10.3389/fphys.2021.668236 (PMC8419471; doi:10.3389/fphys.2021.668236)
Supplement: Supplementary file 4 [file Data_Sheet_1.pdf]

Supplemental File 5. Boxplots of top 20 olfaction-associated genes expressed differentially in Rock compared to other strains. Boxplots represent interquartile range of expression from Rock (n=3) versus the other three strains, whiskers represent first and fourth quartiles, and solid lines in boxes represent median expression values. Circles represent expression values from individual samples. Differential expression was determined using chi-squared goodness of fit tests, and $p<0.05$ represent significantly different genes.

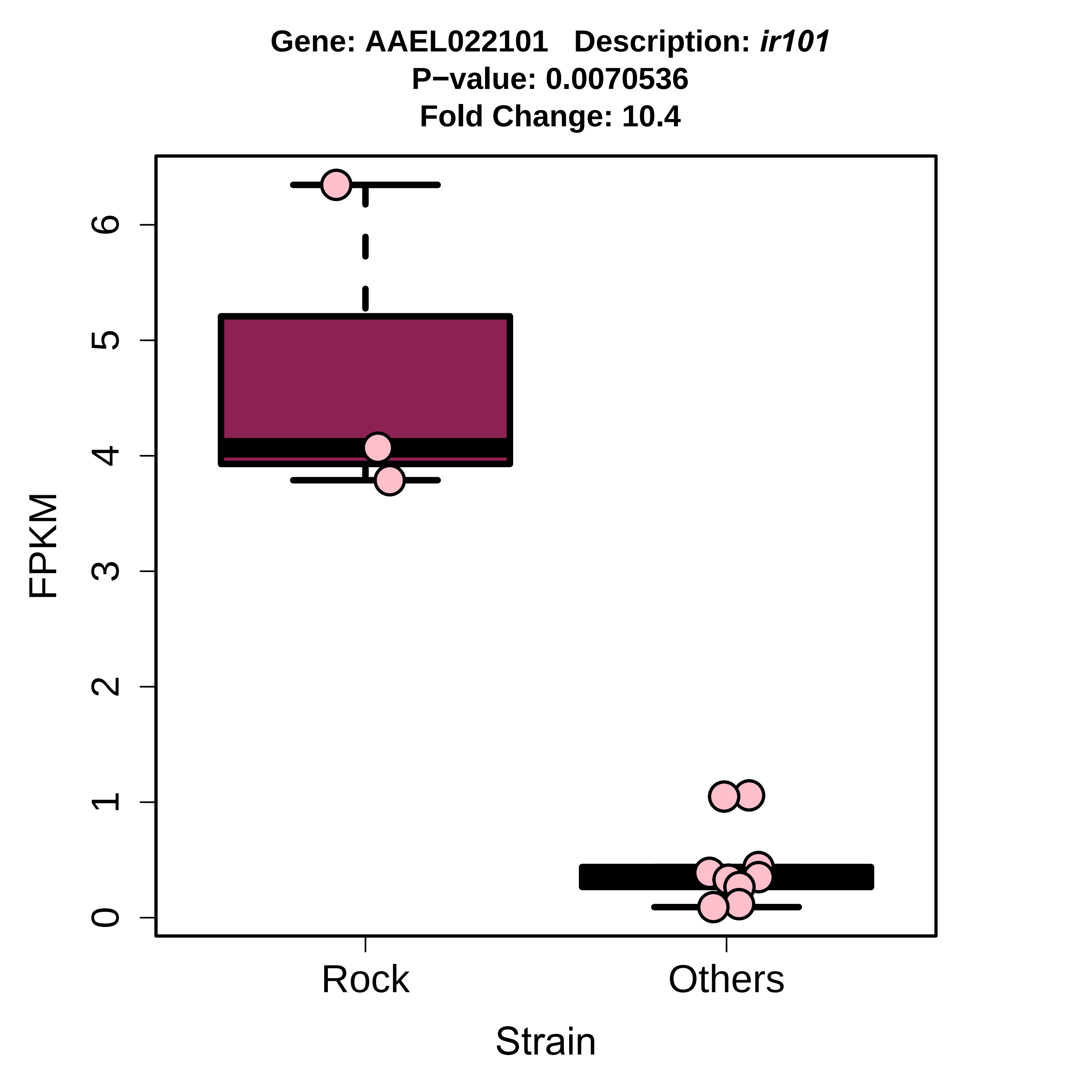
Gene: AAEL022101 Description: *ir101*
P−value: 0.0070536
Fold Change: 10.4
FPKM
0
1
2
3
4
5
6
Rock
Others
Strain

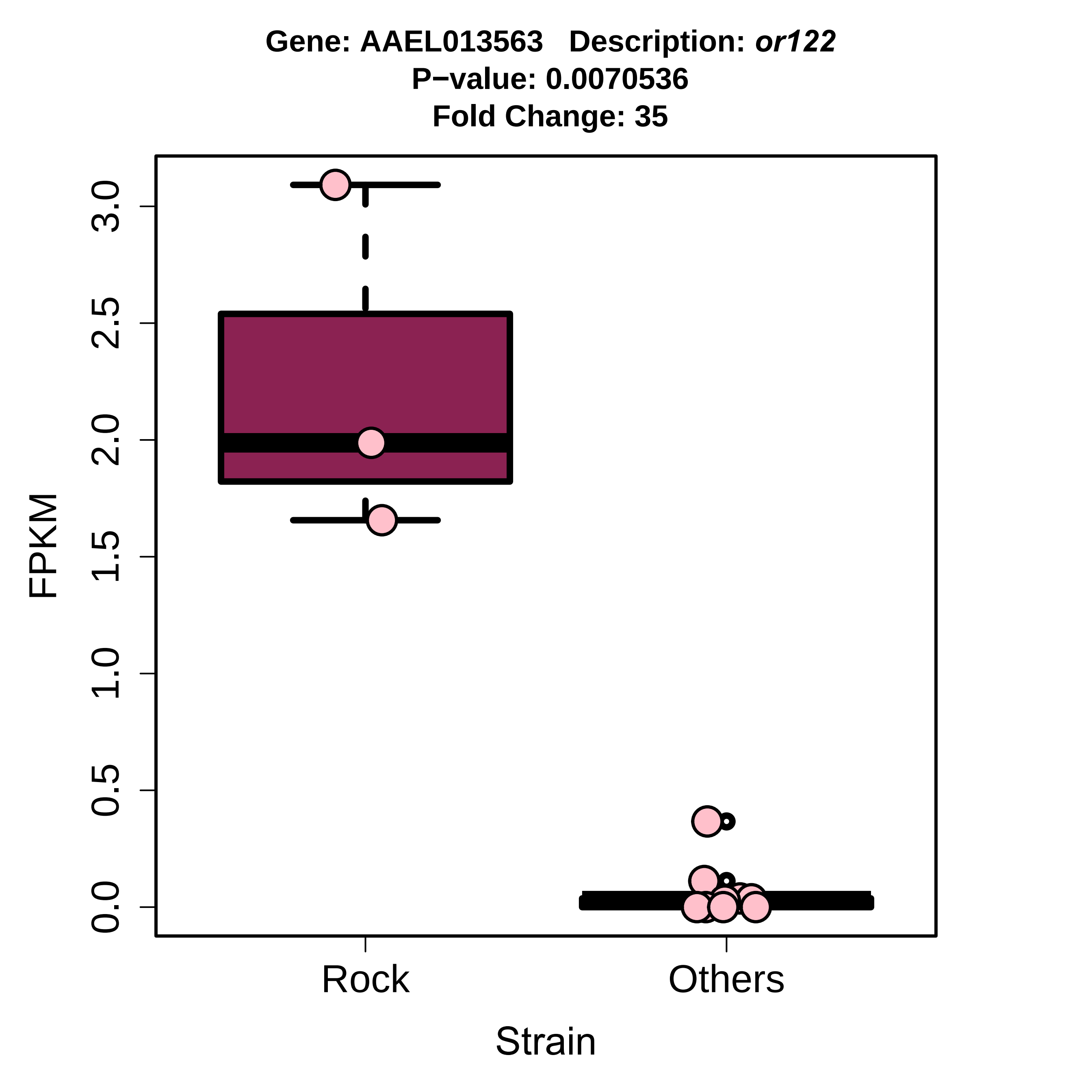
Gene: AAEL013563 Description: *or122*
P−value: 0.0070536
Fold Change: 35
FPKM
0.0
0.5
1.0
1.5
2.0
2.5
3.0
Rock
Others
Strain

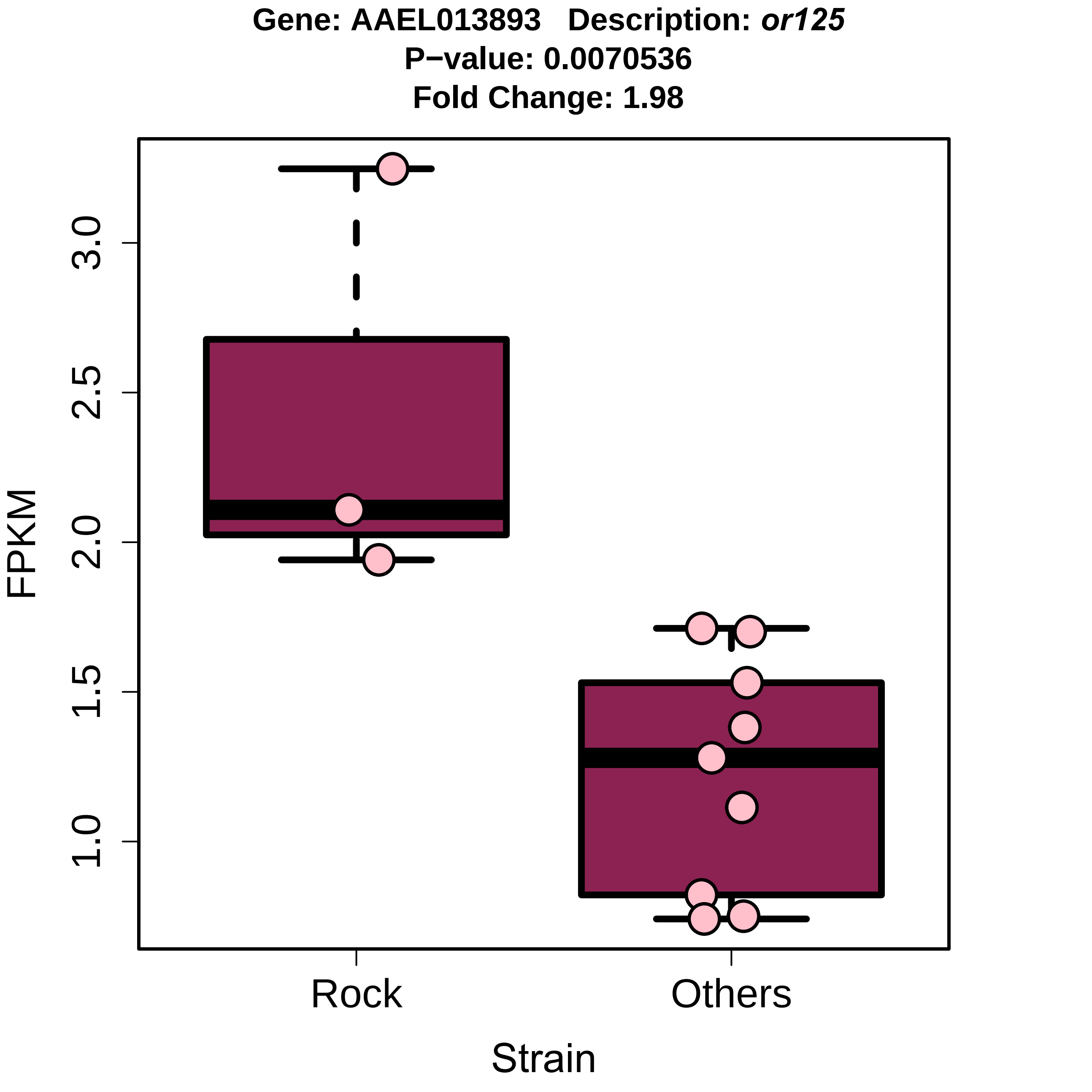
Gene: AAEL013893 Description: *or125*
P−value: 0.0070536
Fold Change: 1.98
FPKM
3.0
2.5
2.0
1.5
1.0
Rock
Others
Strain

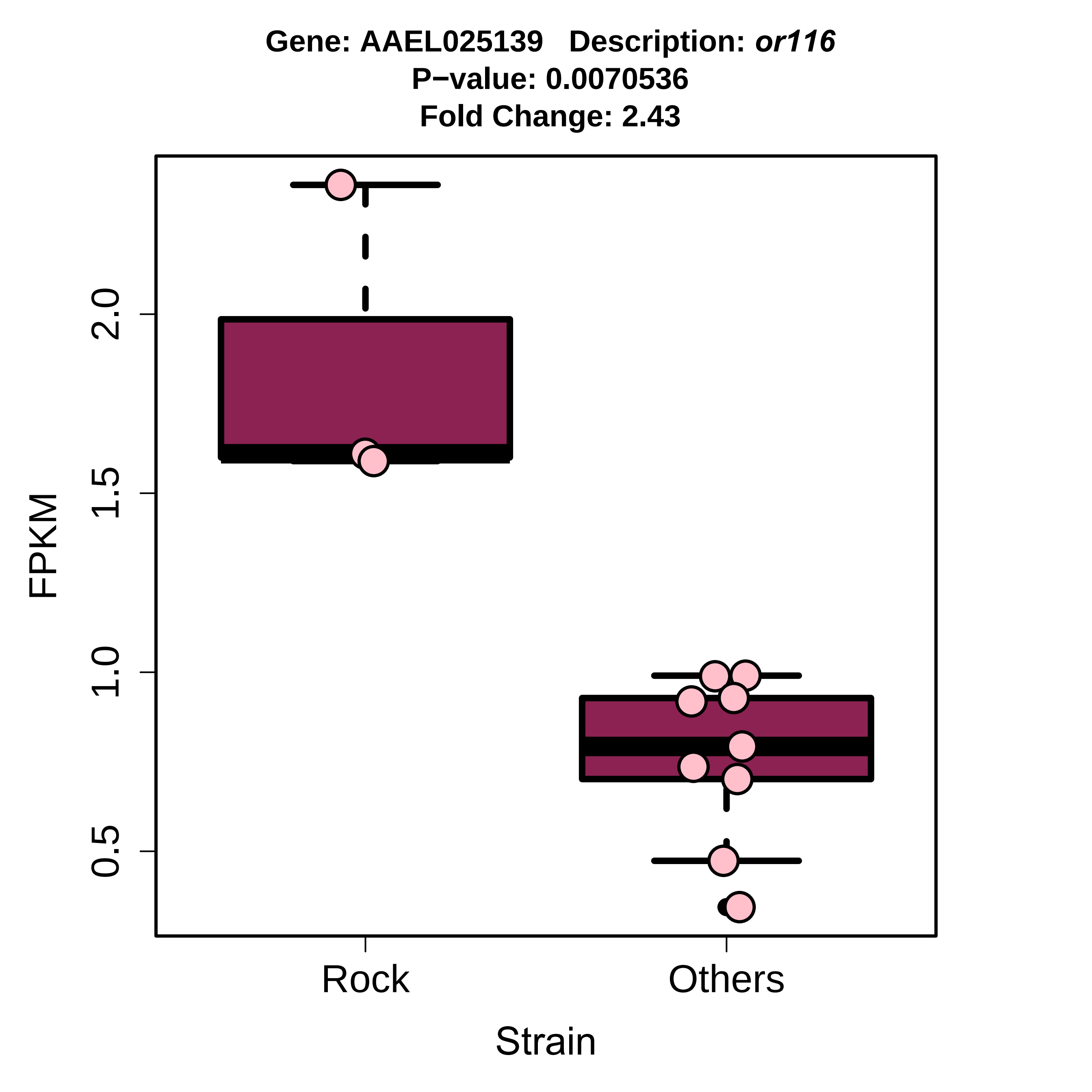

Gene: AAEL025139 Description: *or116*
P−value: 0.0070536
Fold Change: 2.43
FPKM
2.0
1.5
1.0
0.5
Rock
Others
Strain

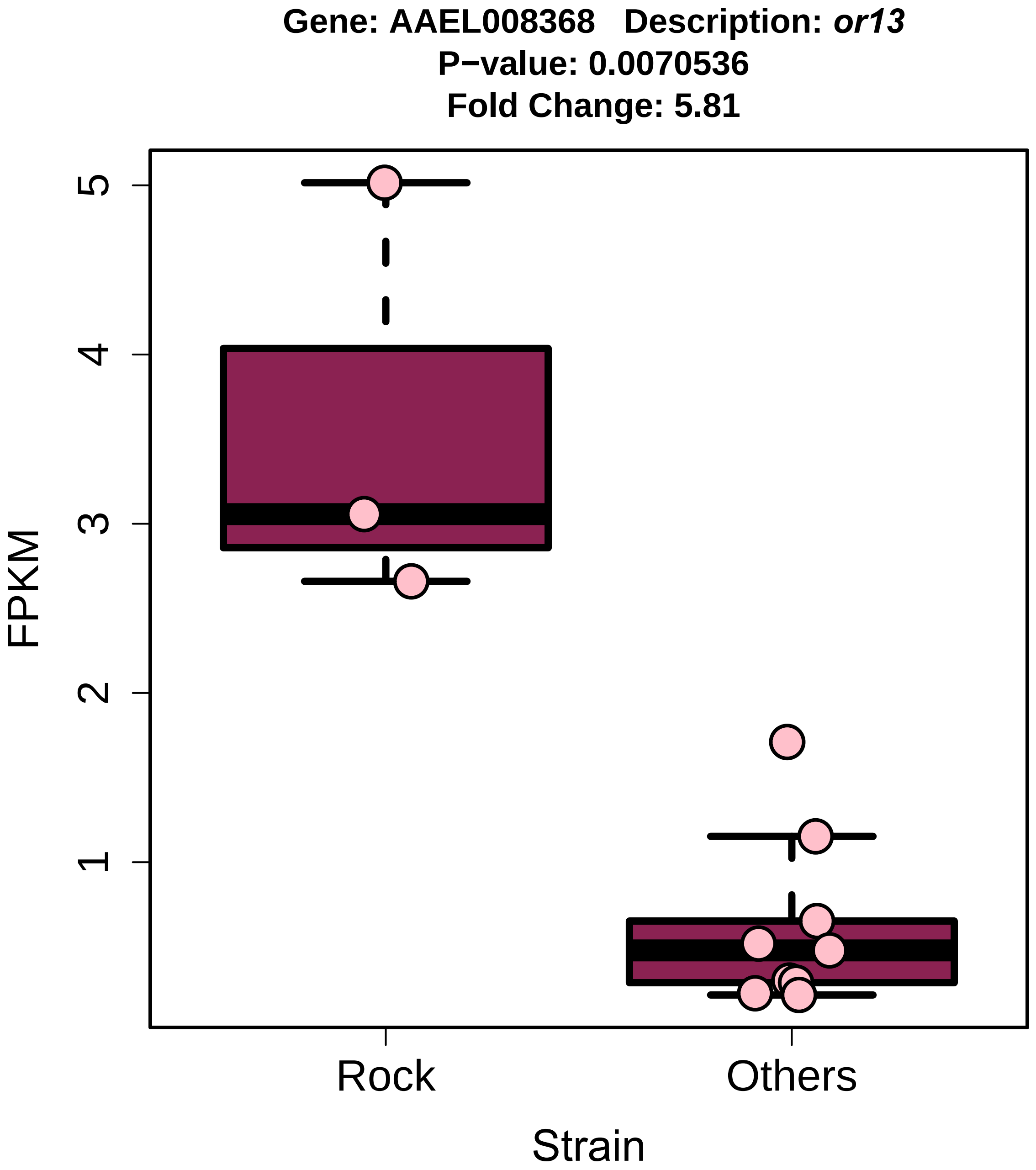
Gene: AAEL008368 Description: or13
P−value: 0.0070536
Fold Change: 5.81
FPKM
1
2
3
4
5
Rock
Others
Strain

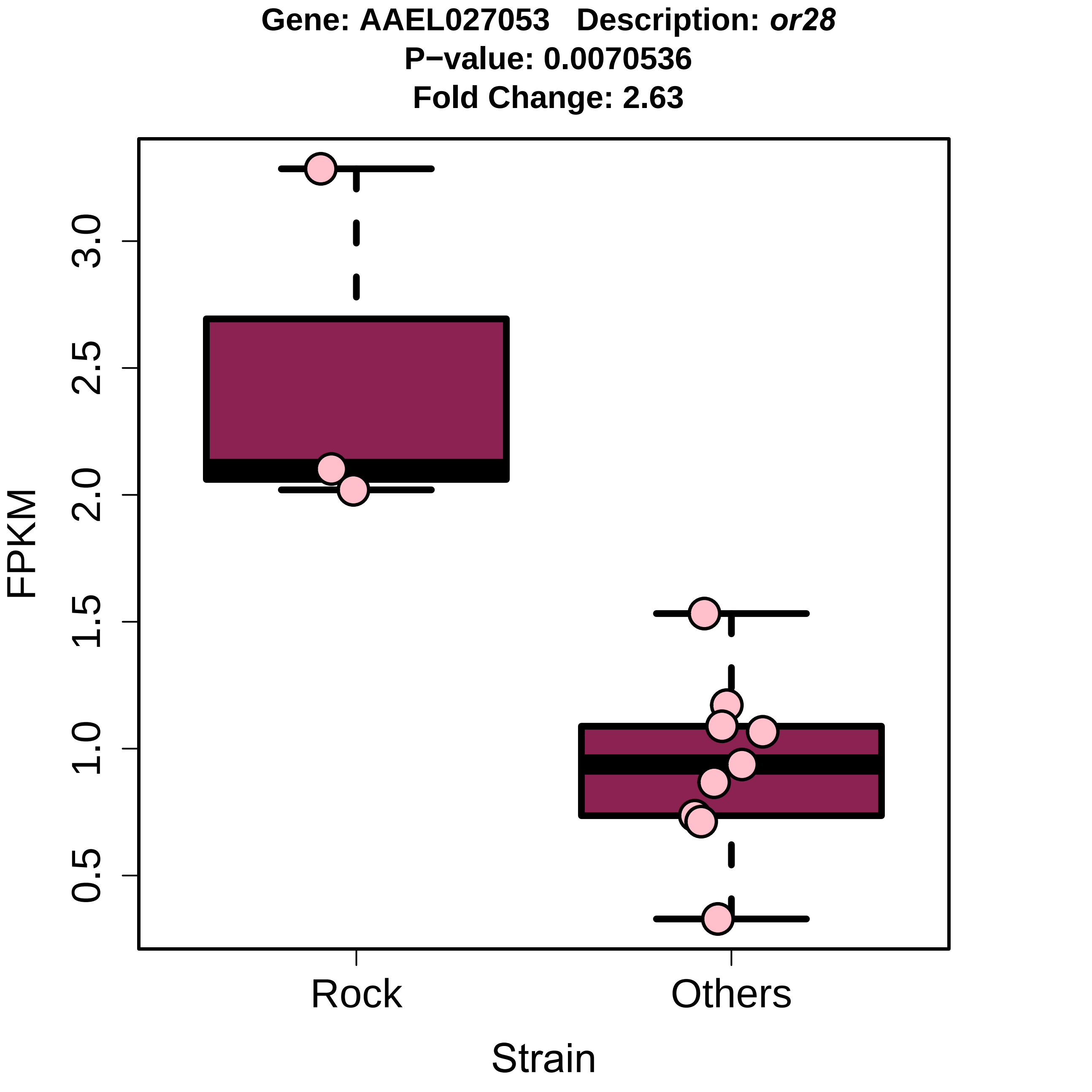

Gene: AAEL027053 Description: *or28*
P−value: 0.0070536
Fold Change: 2.63
FPKM
0.5
1.0
1.5
2.0
2.5
3.0
Rock
Others
Strain

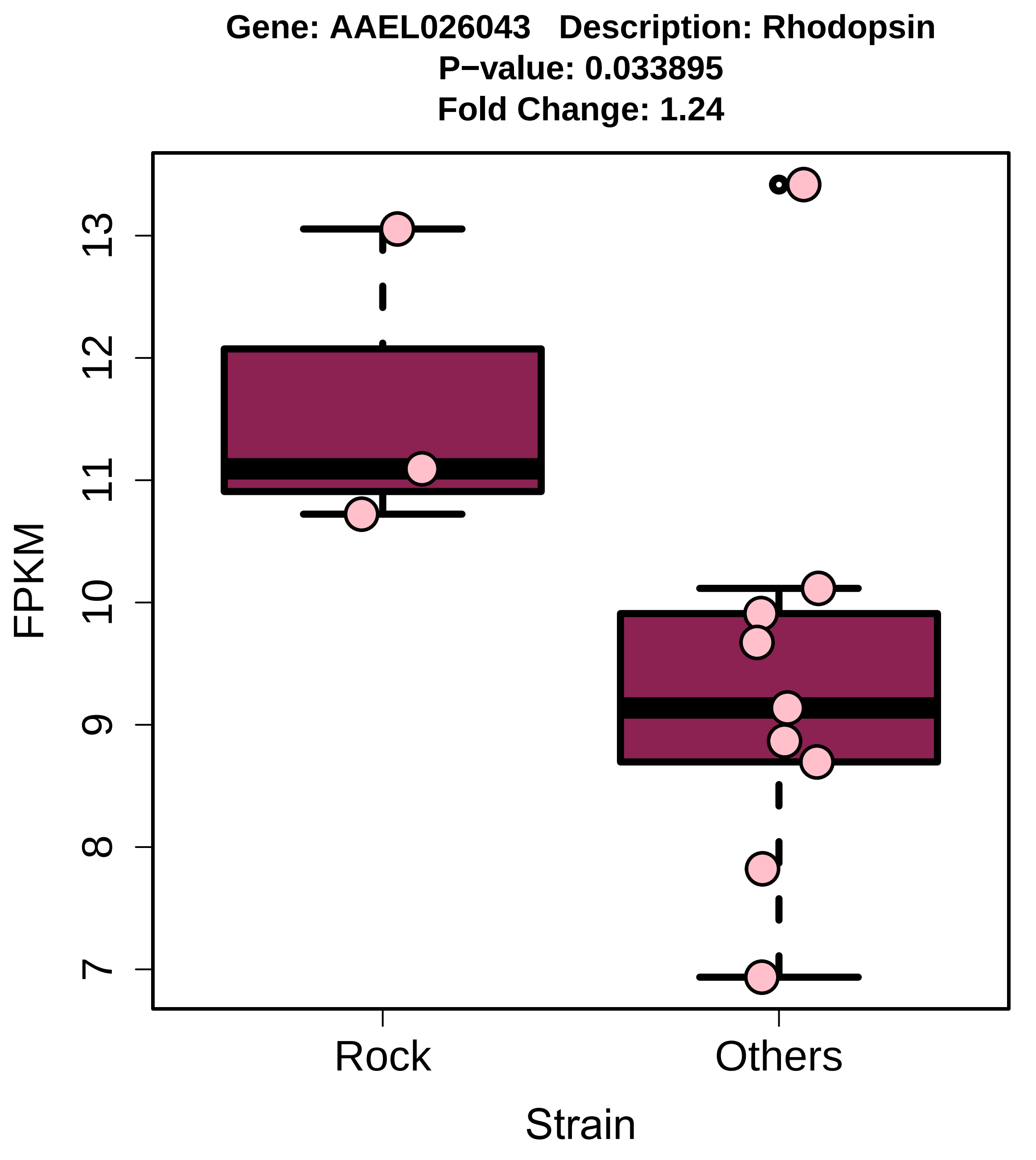
Gene: AAEL026043   Description: Rhodopsin
P−value: 0.033895
Fold Change: 1.24
FPKM
13
12
11
10
9
8
7
Rock
Others
Strain

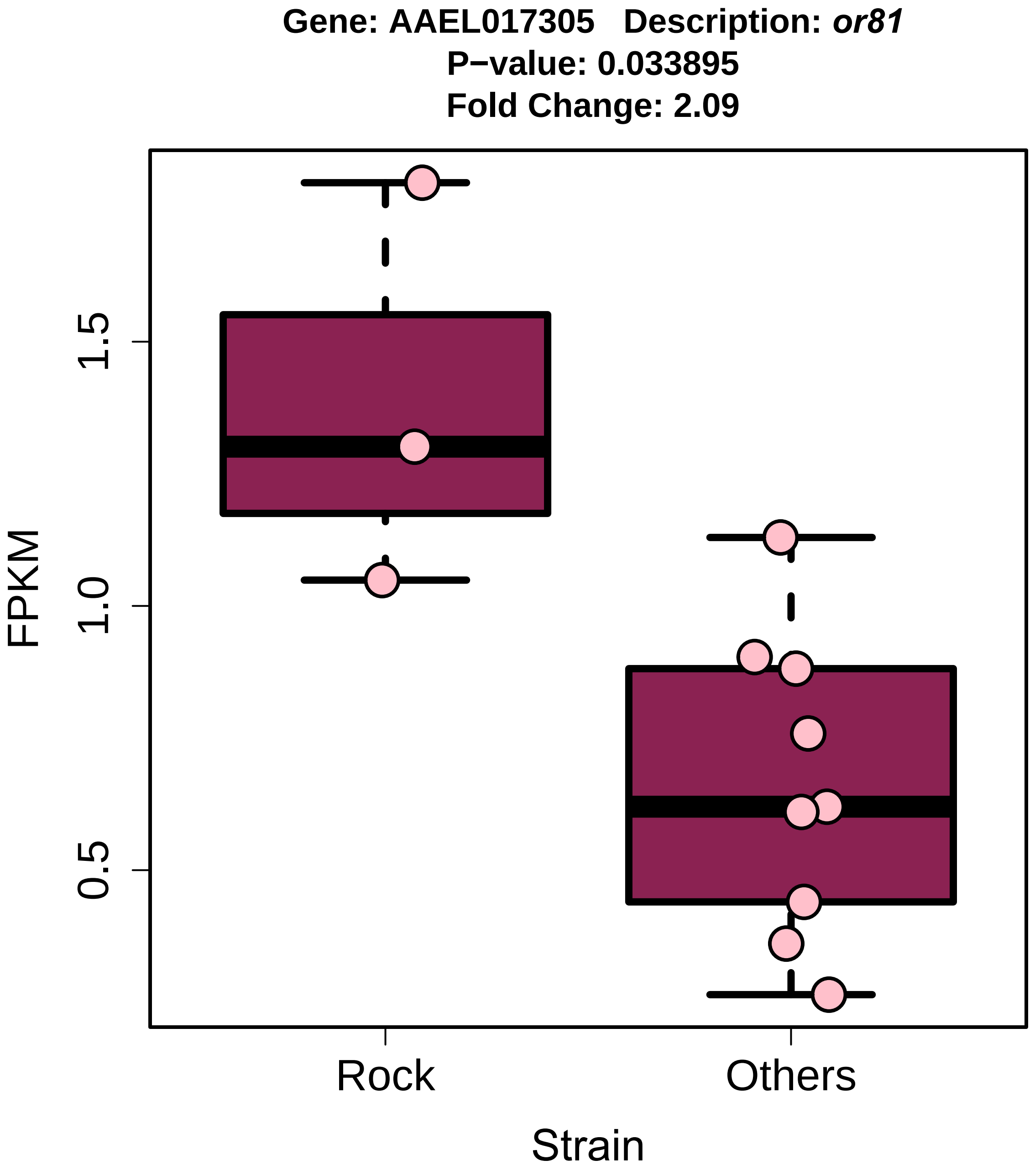

Gene: AAEL017305 Description: *or81*
P−value: 0.033895
Fold Change: 2.09
FPKM
1.5
1.0
0.5
Rock
Others
Strain

Gene: AAEL023000   Description: Arrestin
P−value: 0.033895
Fold Change: 2.92

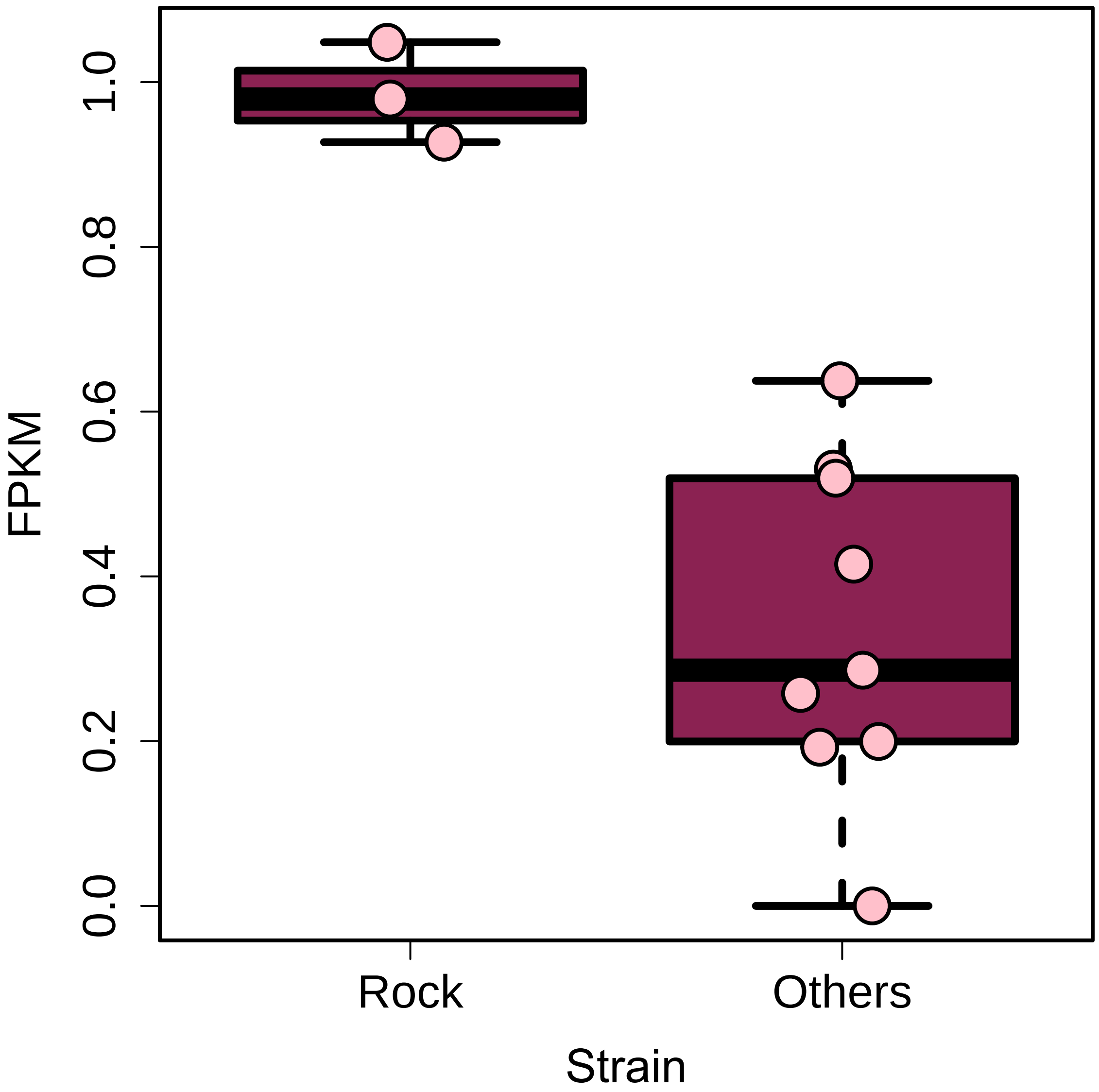

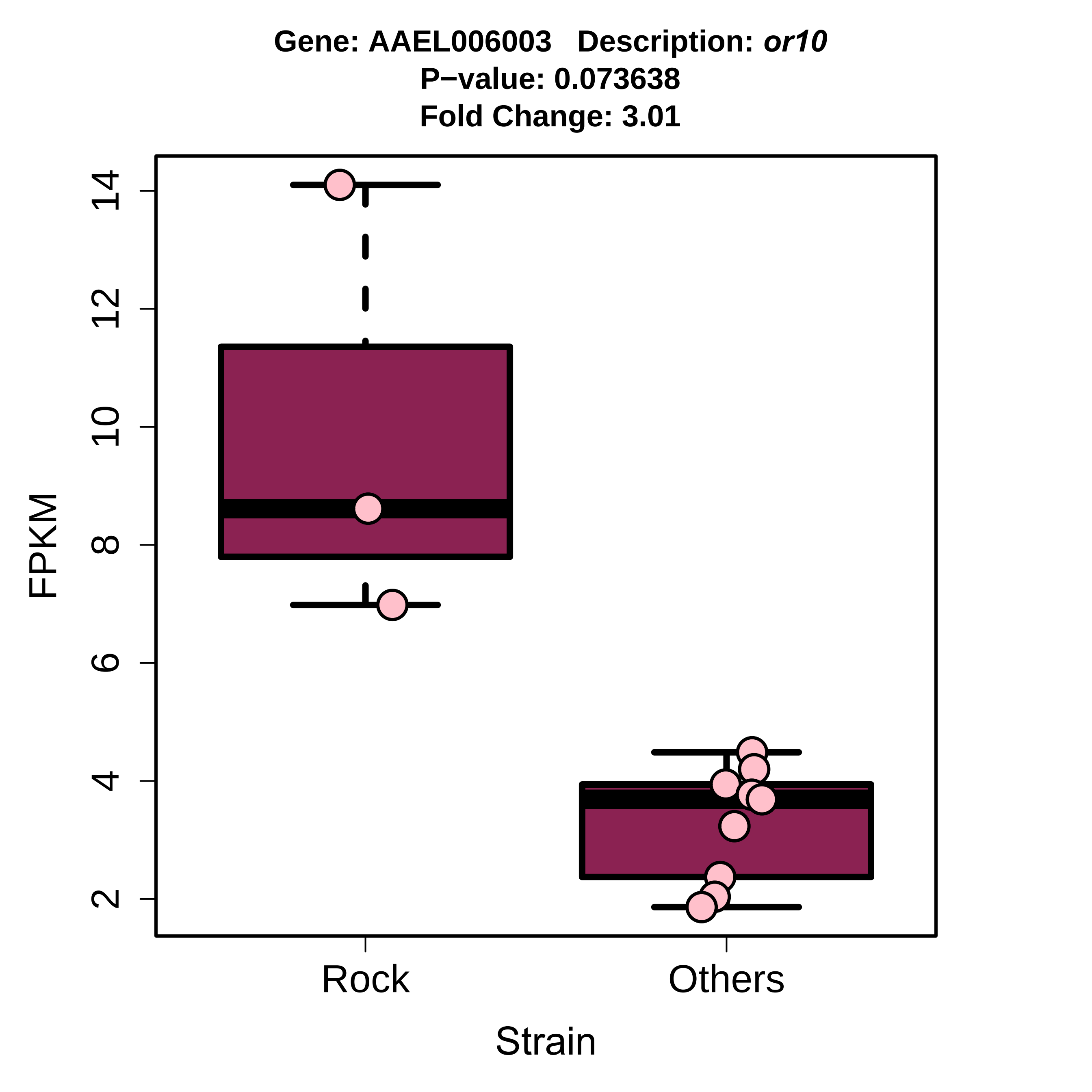

Gene: AAEL006003 Description: *or10*
P−value: 0.073638
Fold Change: 3.01
FPKM
2
4
6
8
10
12
14
Rock
Others
Strain

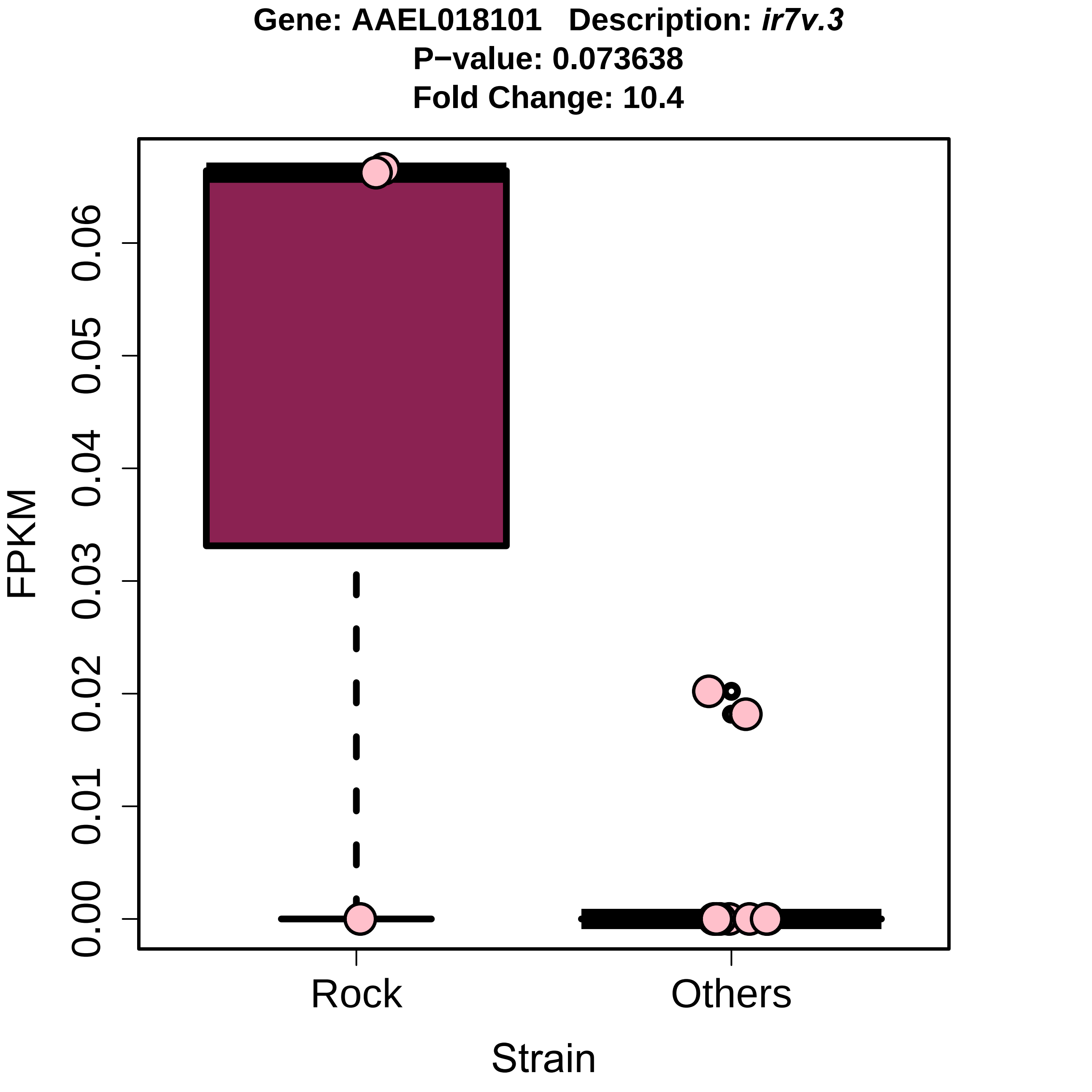

Gene: AAEL018101 Description: *ir7v.3*
P−value: 0.073638
Fold Change: 10.4
FPKM
0.00
0.01
0.02
0.03
0.04
0.05
0.06
Rock
Others
Strain

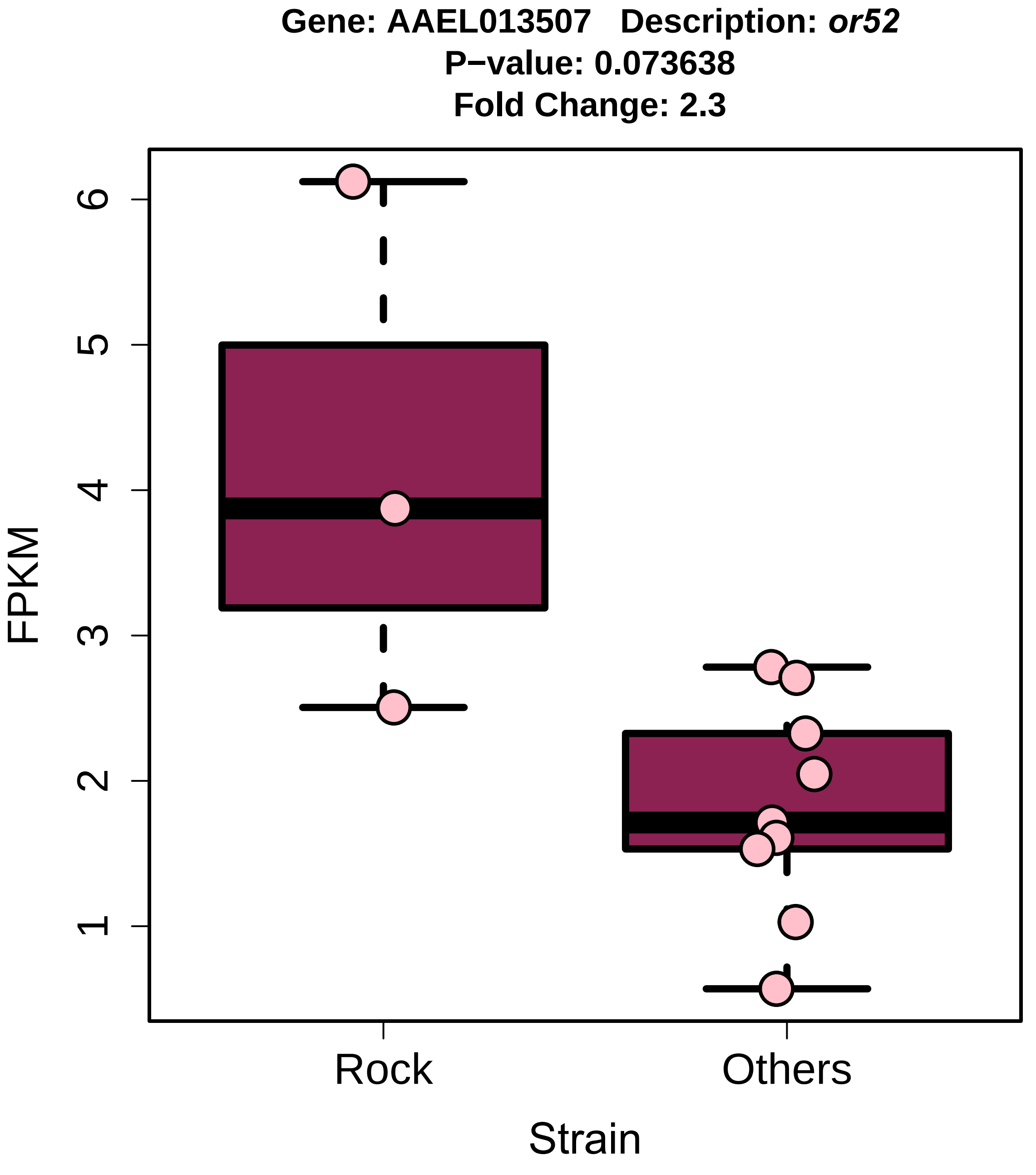
Gene: AAEL013507 Description: *or52*
P−value: 0.073638
Fold Change: 2.3
FPKM
1
2
3
4
5
6
Rock
Others
Strain

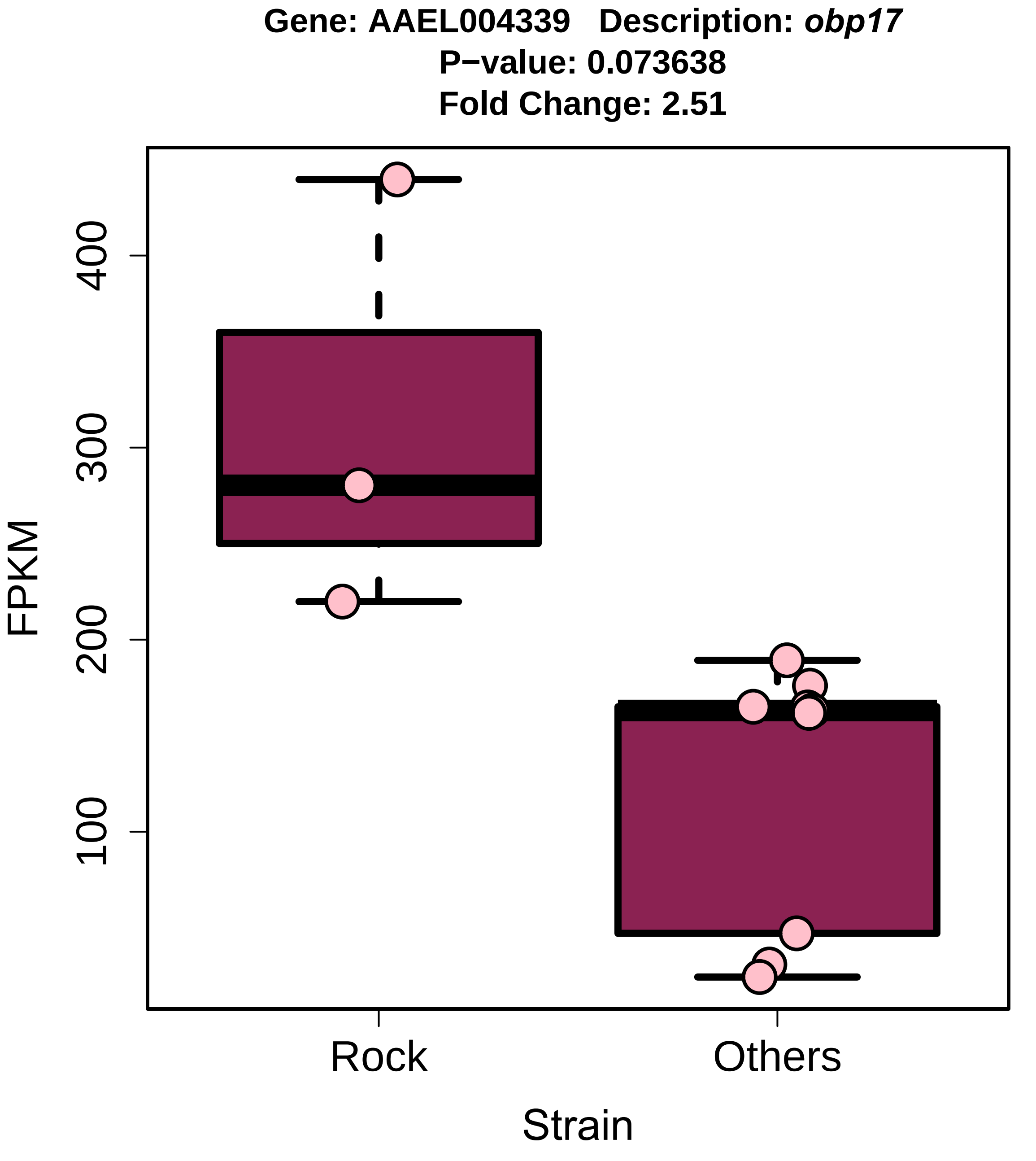

Gene: AAEL004339 Description: *obp17*
P−value: 0.073638
Fold Change: 2.51
FPKM
100
200
300
400
Rock
Others
Strain

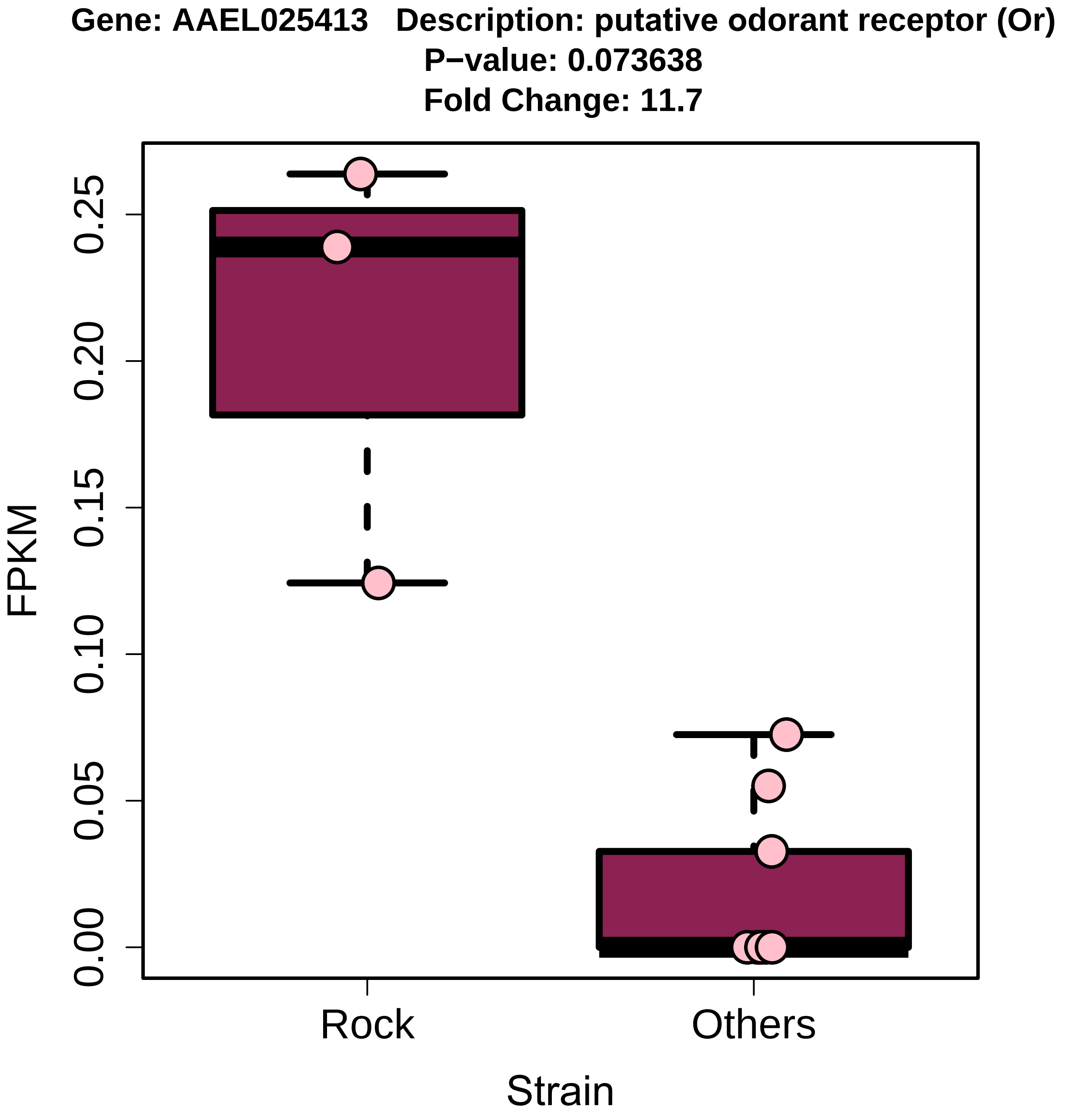

Gene: AAEL025413 Description: putative odorant receptor (Or)
P−value: 0.073638
Fold Change: 11.7
FPKM
0.00
0.05
0.10
0.15
0.20
0.25
Rock
Others
Strain

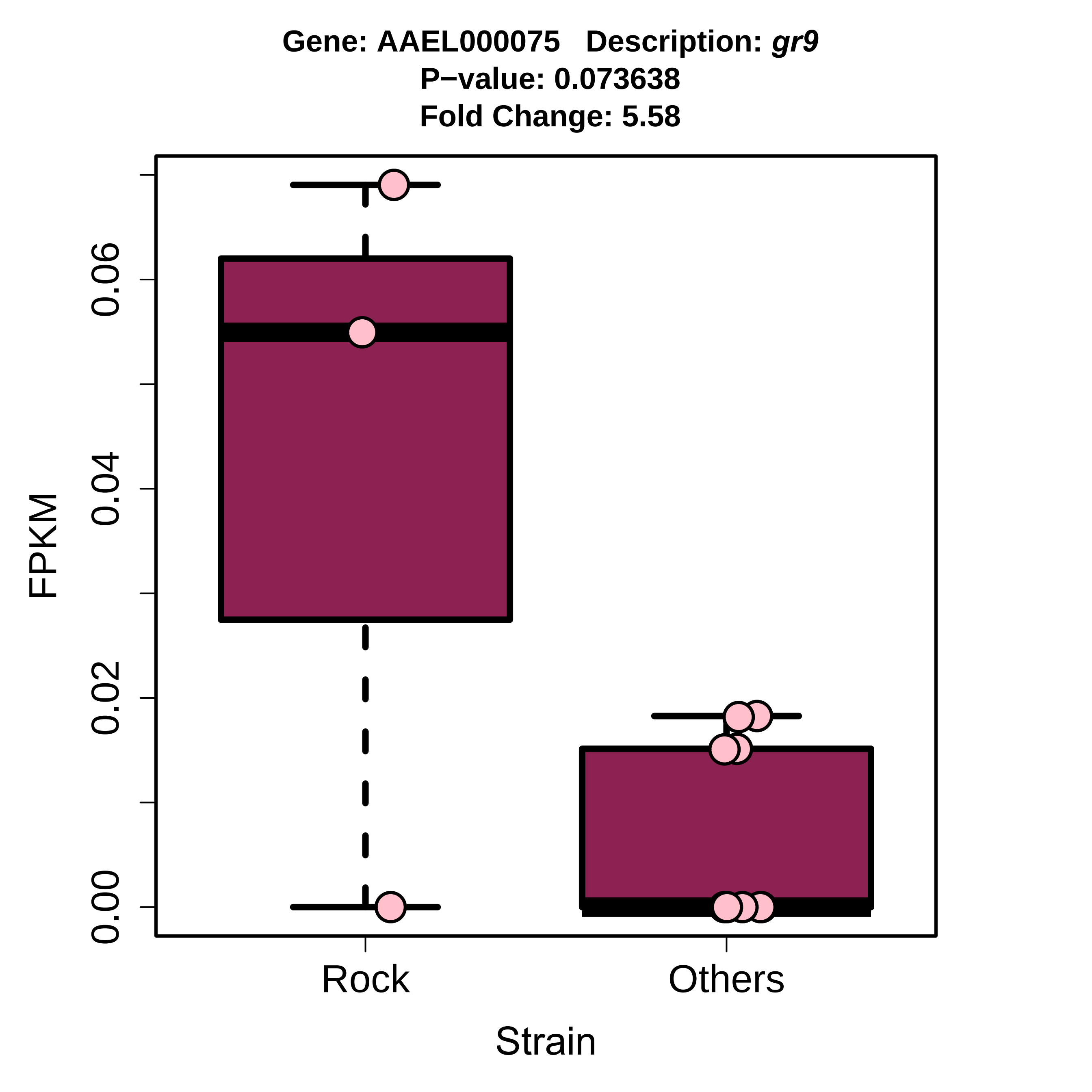
Gene: AAEL000075 Description: gr9
P−value: 0.073638
Fold Change: 5.58
FPKM
0.00
0.02
0.04
0.06
Rock
Others
Strain

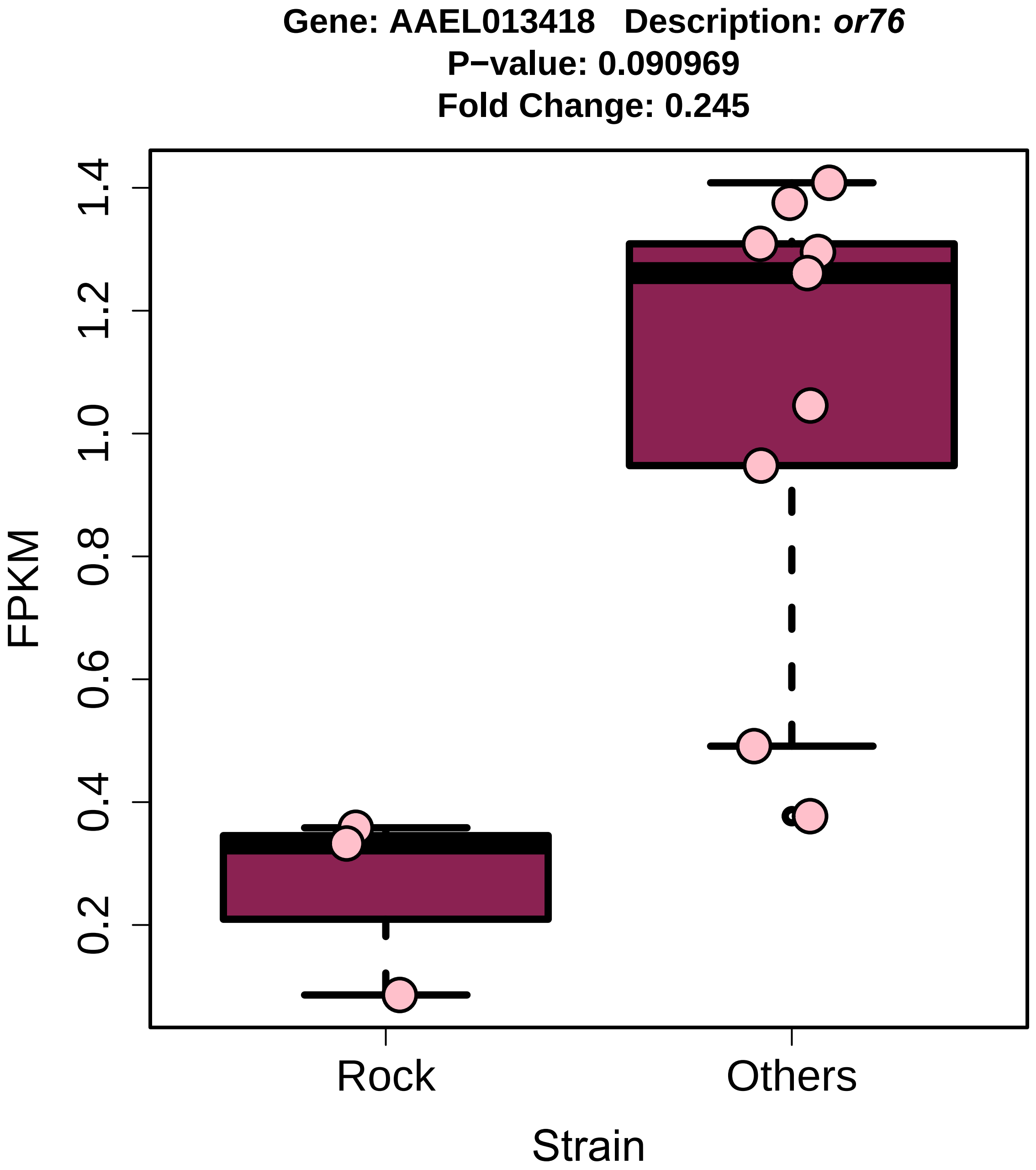
Gene: AAEL013418 Description: or76
P−value: 0.090969
Fold Change: 0.245
FPKM
1.4
1.2
1.0
0.8
0.6
0.4
0.2
Rock
Others
Strain

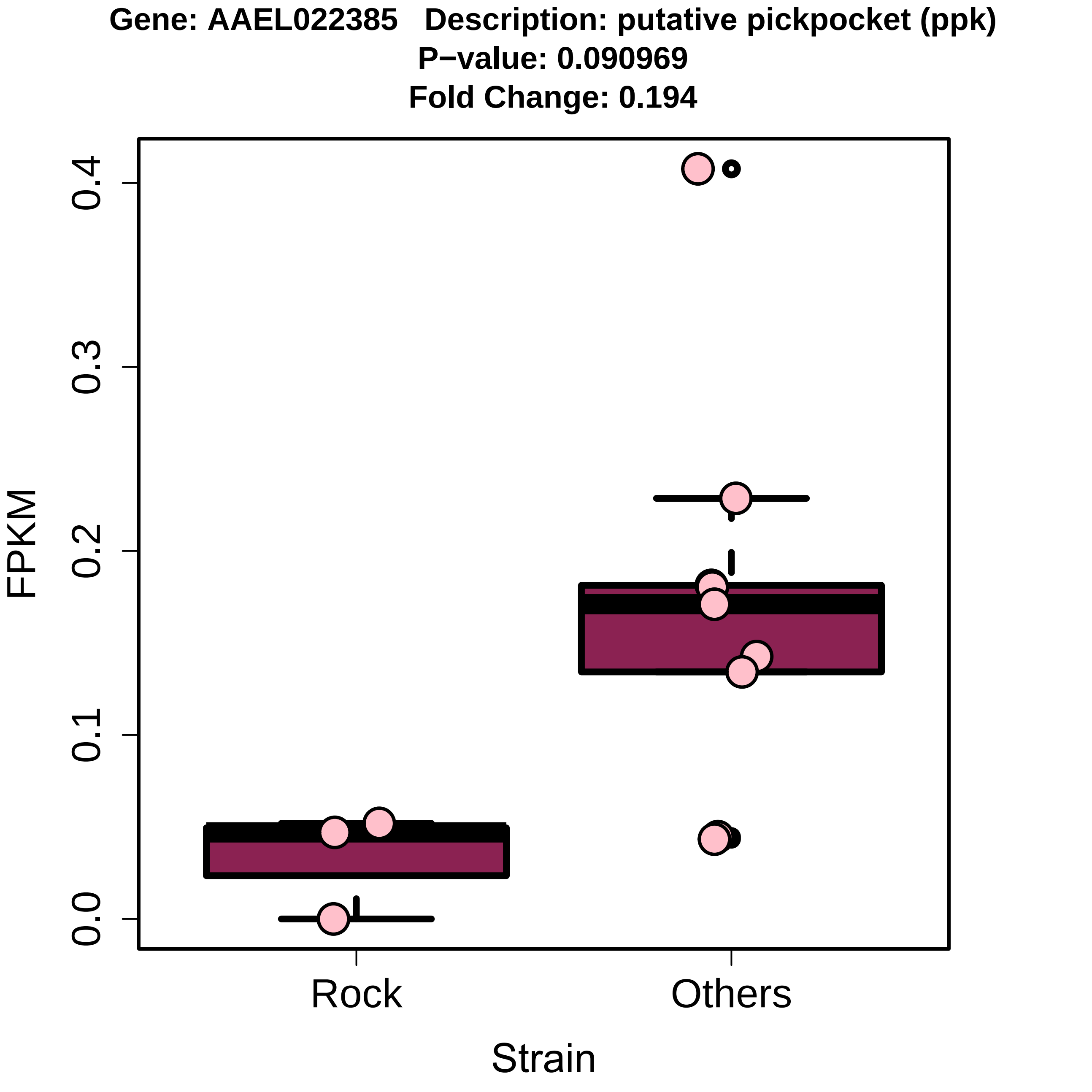

Gene: AAEL022385   Description: putative pickpocket (ppk)
P−value: 0.090969
Fold Change: 0.194
FPKM
0.0
0.1
0.2
0.3
0.4
Rock
Others
Strain

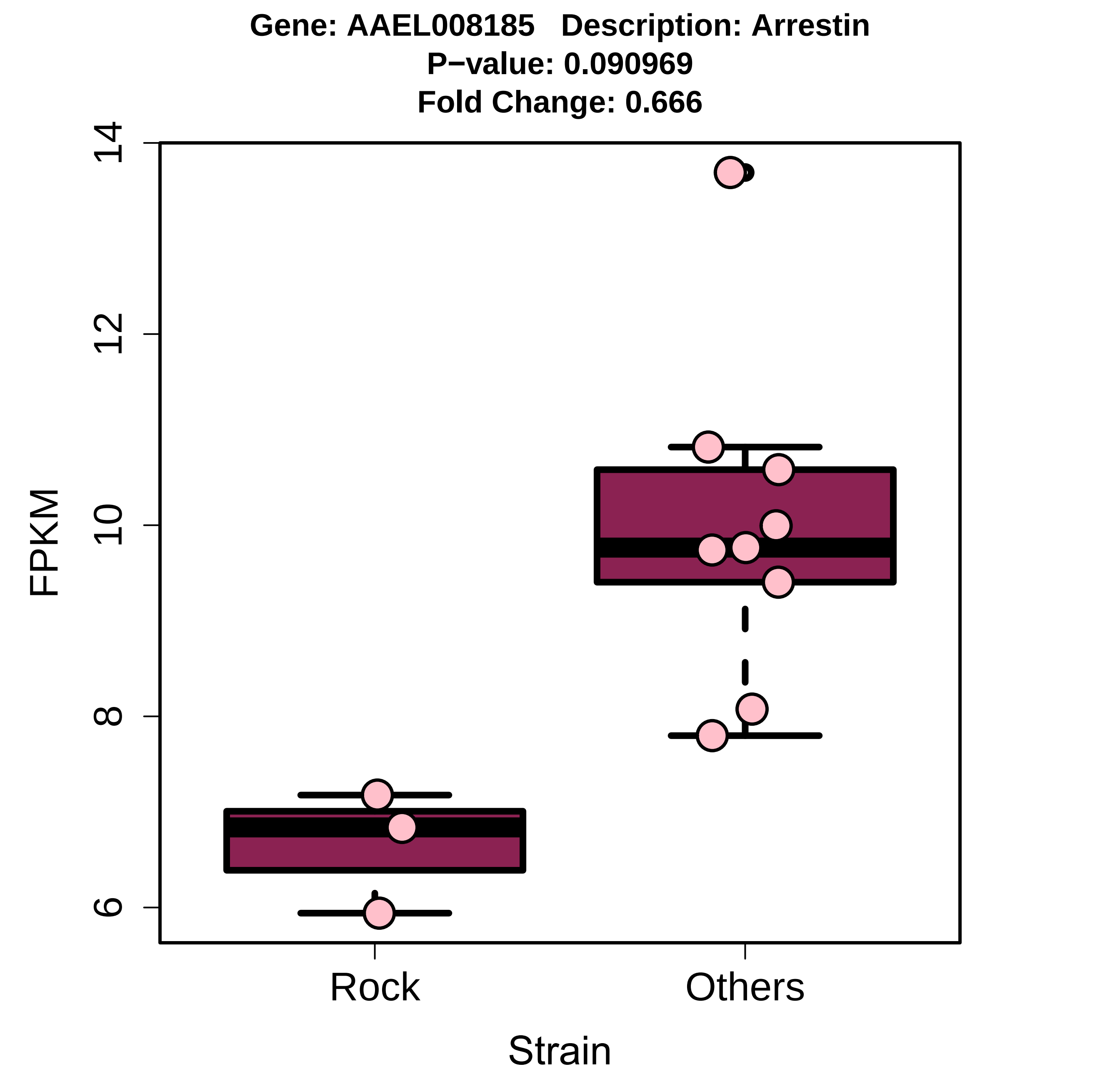
Gene: AAEL008185 Description: Arrestin
P−value: 0.090969
Fold Change: 0.666
14
12
10
8
6
FPKM
Rock
Others
Strain

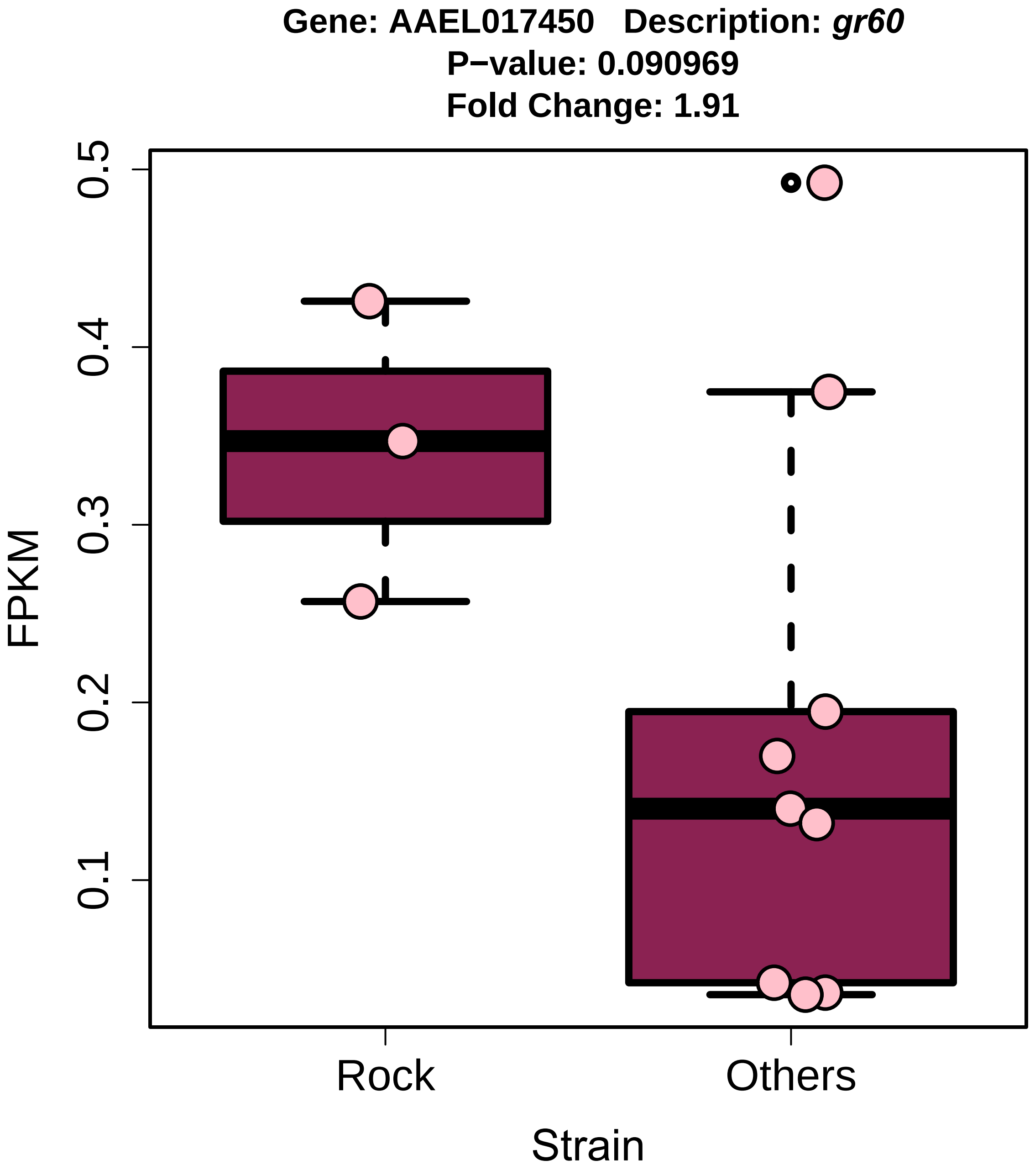

Gene: AAEL017450 Description: *gr60*
P−value: 0.090969
Fold Change: 1.91
FPKM
0.1
0.2
0.3
0.4
0.5
Rock
Others
Strain

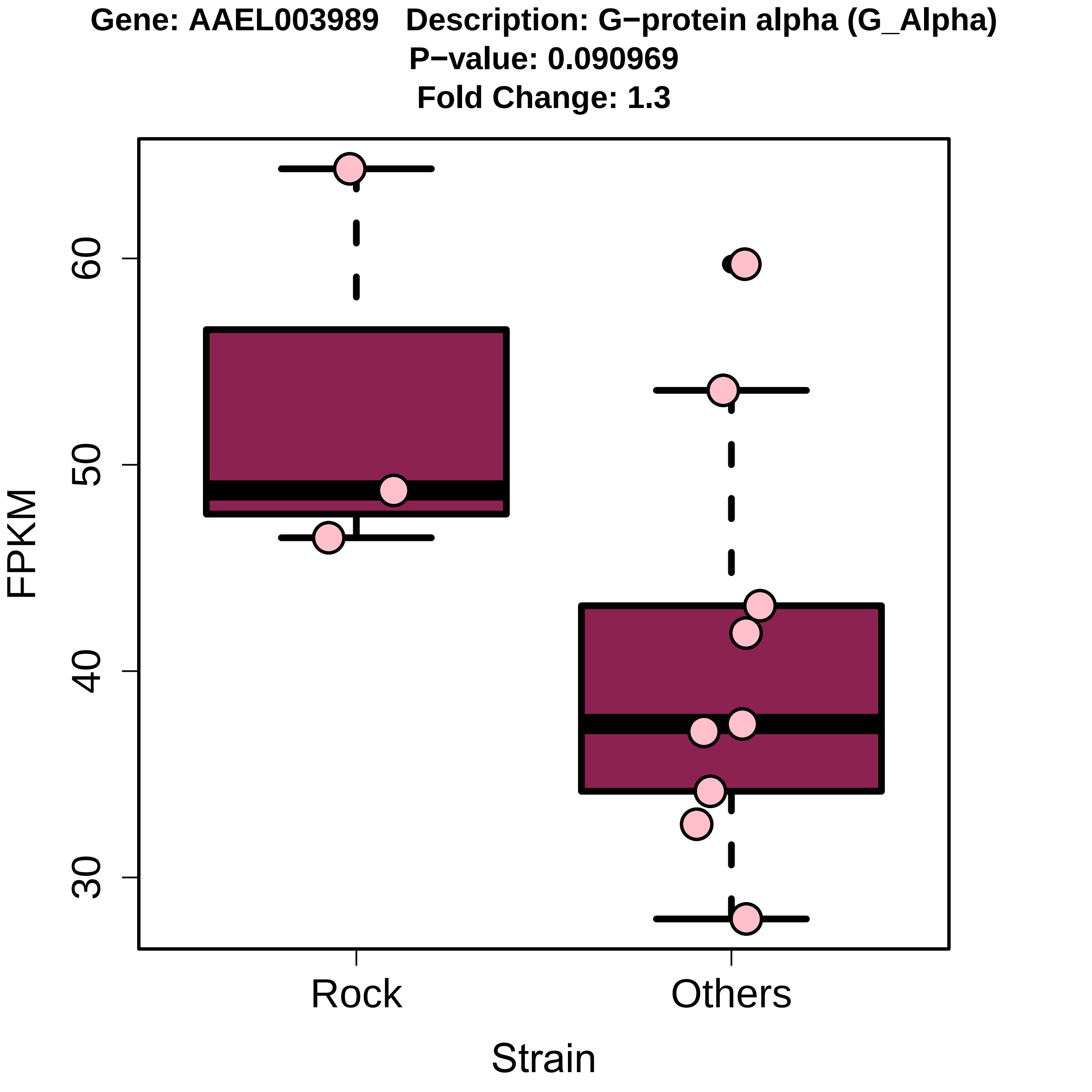
Gene: AAEL003989   Description: G−protein alpha (G_Alpha)
P−value: 0.090969
Fold Change: 1.3
FPKM
60
50
40
30
Rock
Others
Strain
