# Supplementary material for: Olfaction-Related Gene Expression in the Antennae of Female Mosquitoes From Common Aedes aegypti Laboratory Strains
Source: Front Physiol. 2021 Aug 23;12:668236. doi: 10.3389/fphys.2021.668236 (PMC8419471; doi:10.3389/fphys.2021.668236)

Supplemental File 6. Boxplots of top 20 olfaction-associated genes expressed differentially in Liverpool compared to other strains. Boxplots represent interquartile range of expression from Liverpool (n=3) versus the other three strains, whiskers represent first and fourth quartiles, and solid lines in boxes represent median expression values. Circles represent expression values from individual samples. Differential expression was determined using chi-squared goodness of fit tests, and $p<0.05$ represent significantly different genes.

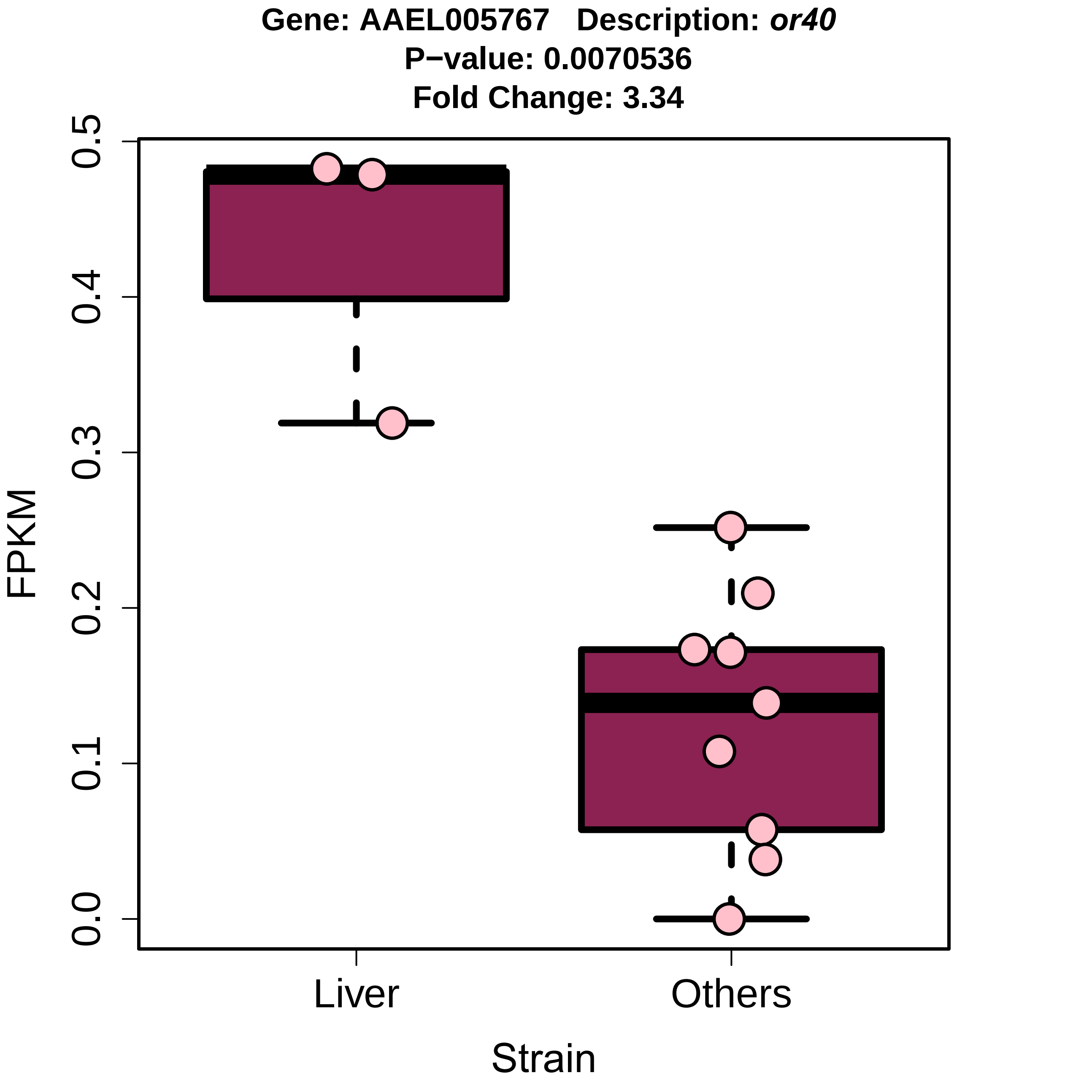
Gene: AAEL005767 Description: *or40*
P−value: 0.0070536
Fold Change: 3.34
FPKM
0.0
0.1
0.2
0.3
0.4
0.5
Liver
Others
Strain

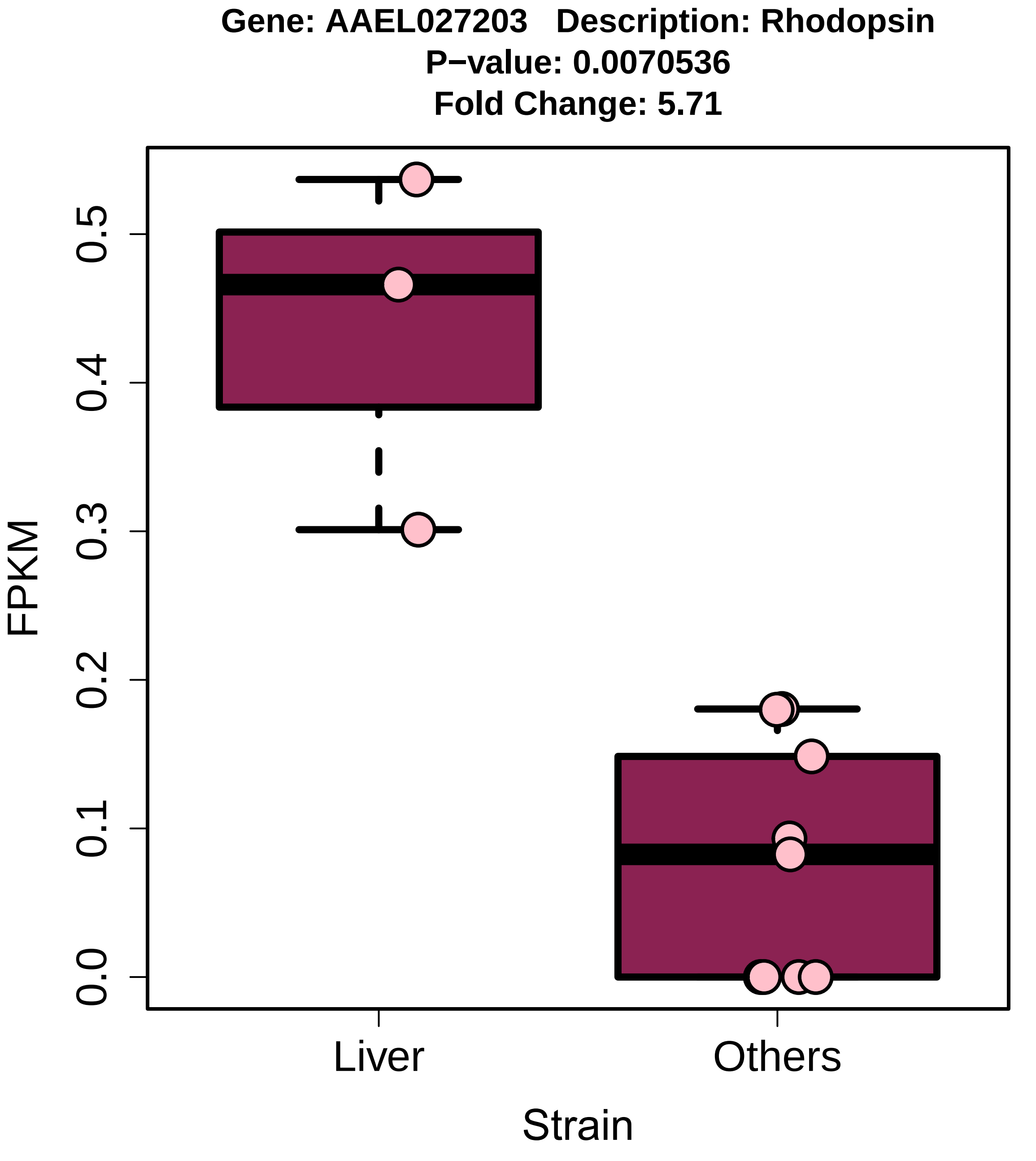

Gene: AAEL027203 Description: Rhodopsin
P−value: 0.0070536
Fold Change: 5.71
FPKM
0.0
0.1
0.2
0.3
0.4
0.5
Liver
Others
Strain

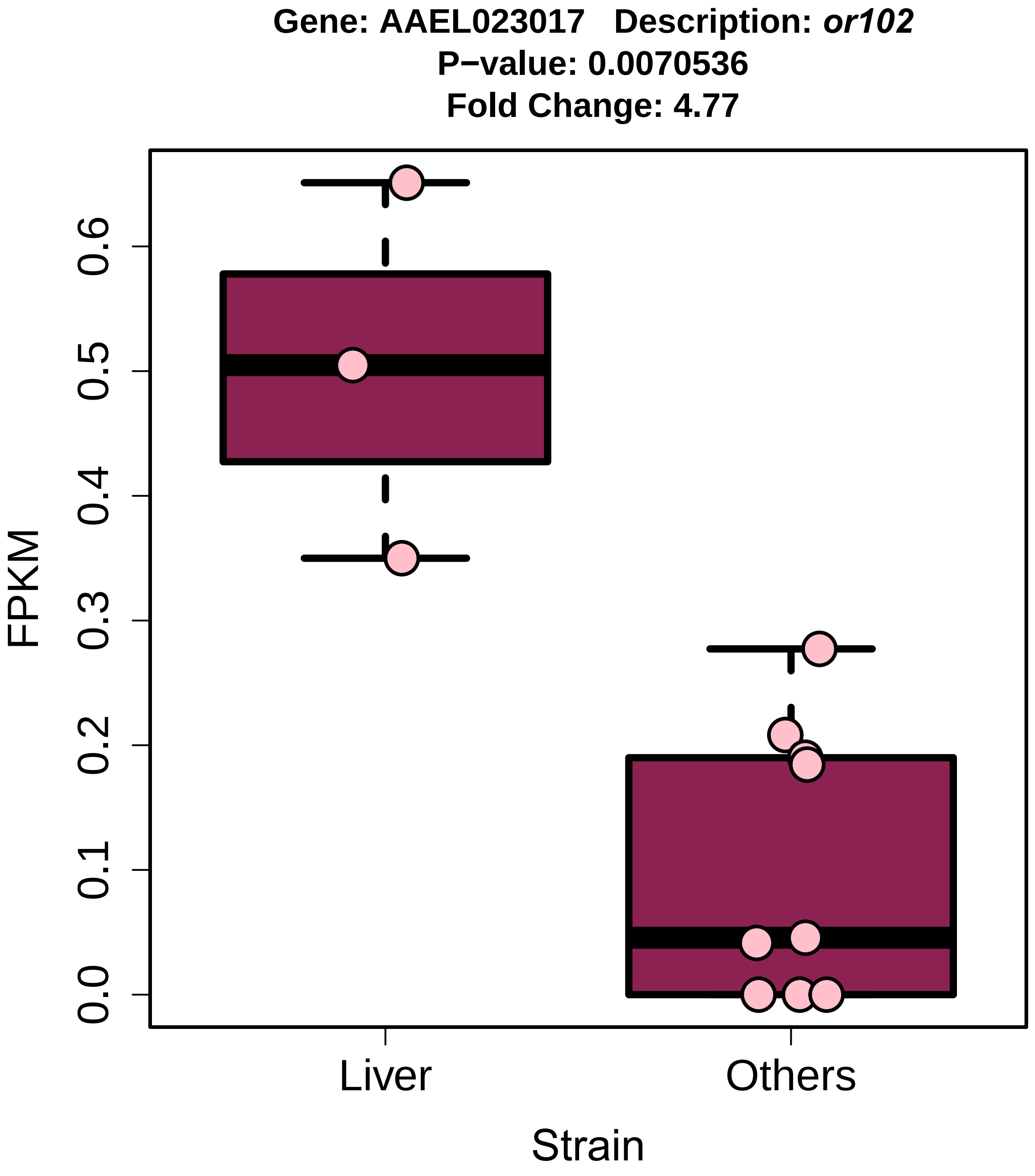

Gene: AAEL023017 Description: *or102*
P−value: 0.0070536
Fold Change: 4.77
FPKM
0.0
0.1
0.2
0.3
0.4
0.5
0.6
Liver
Others
Strain

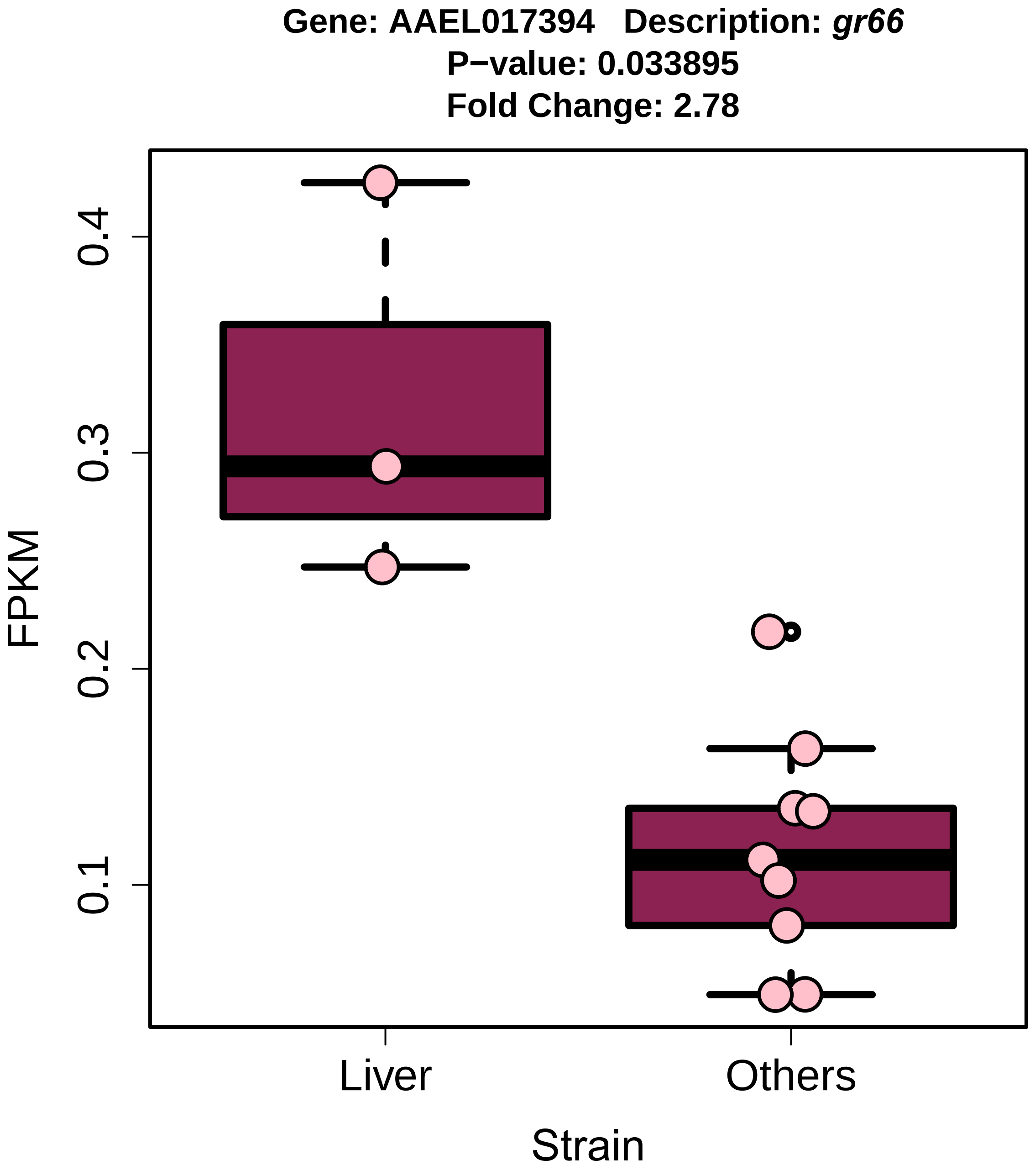

Gene: AAEL017394 Description: *gr66*
P−value: 0.033895
Fold Change: 2.78
FPKM
0.1
0.2
0.3
0.4
Liver
Others
Strain

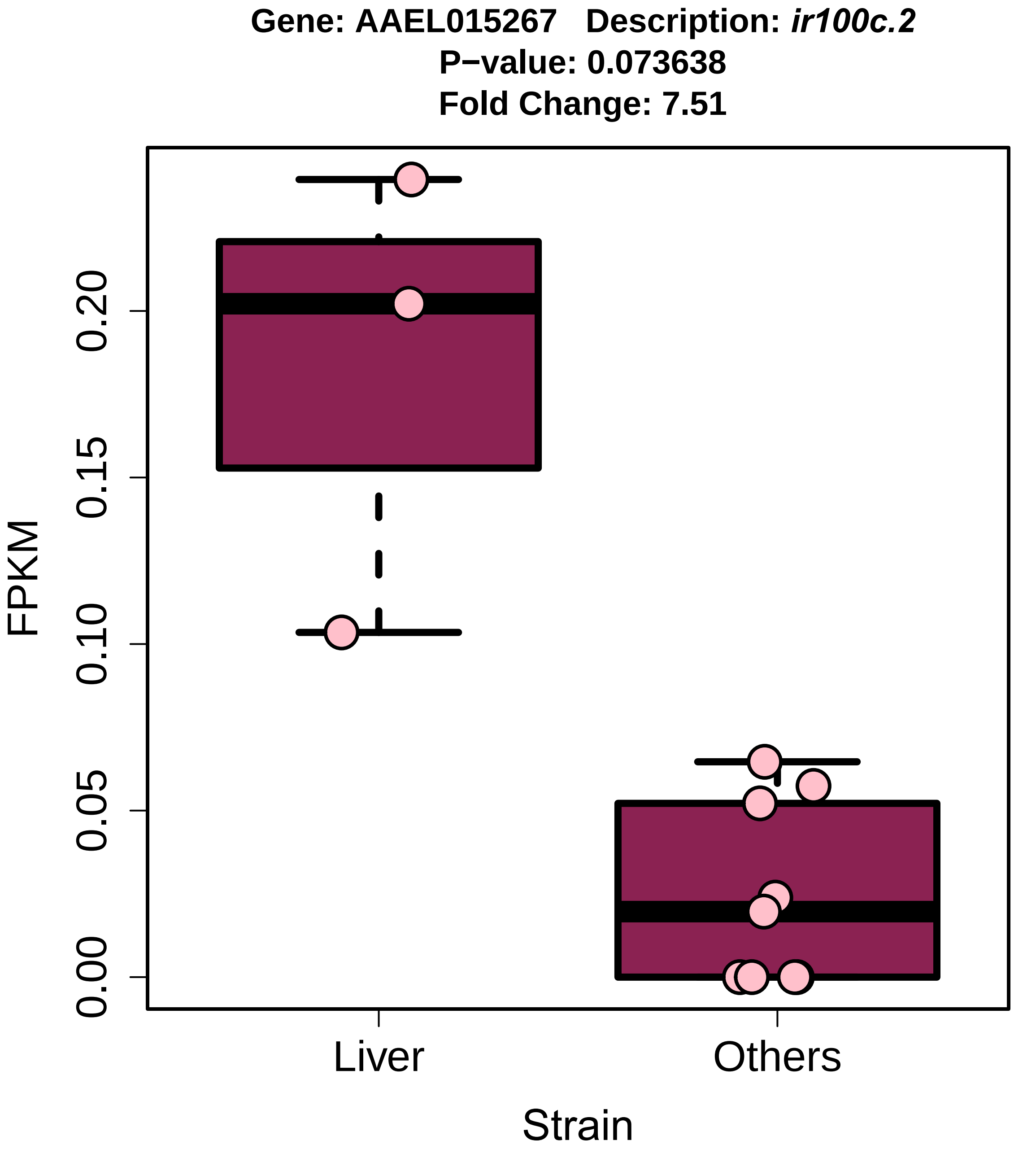
Gene: AAEL015267 Description: *ir100c.2*
P−value: 0.073638
Fold Change: 7.51
FPKM
0.00
0.05
0.10
0.15
0.20
Liver
Others
Strain

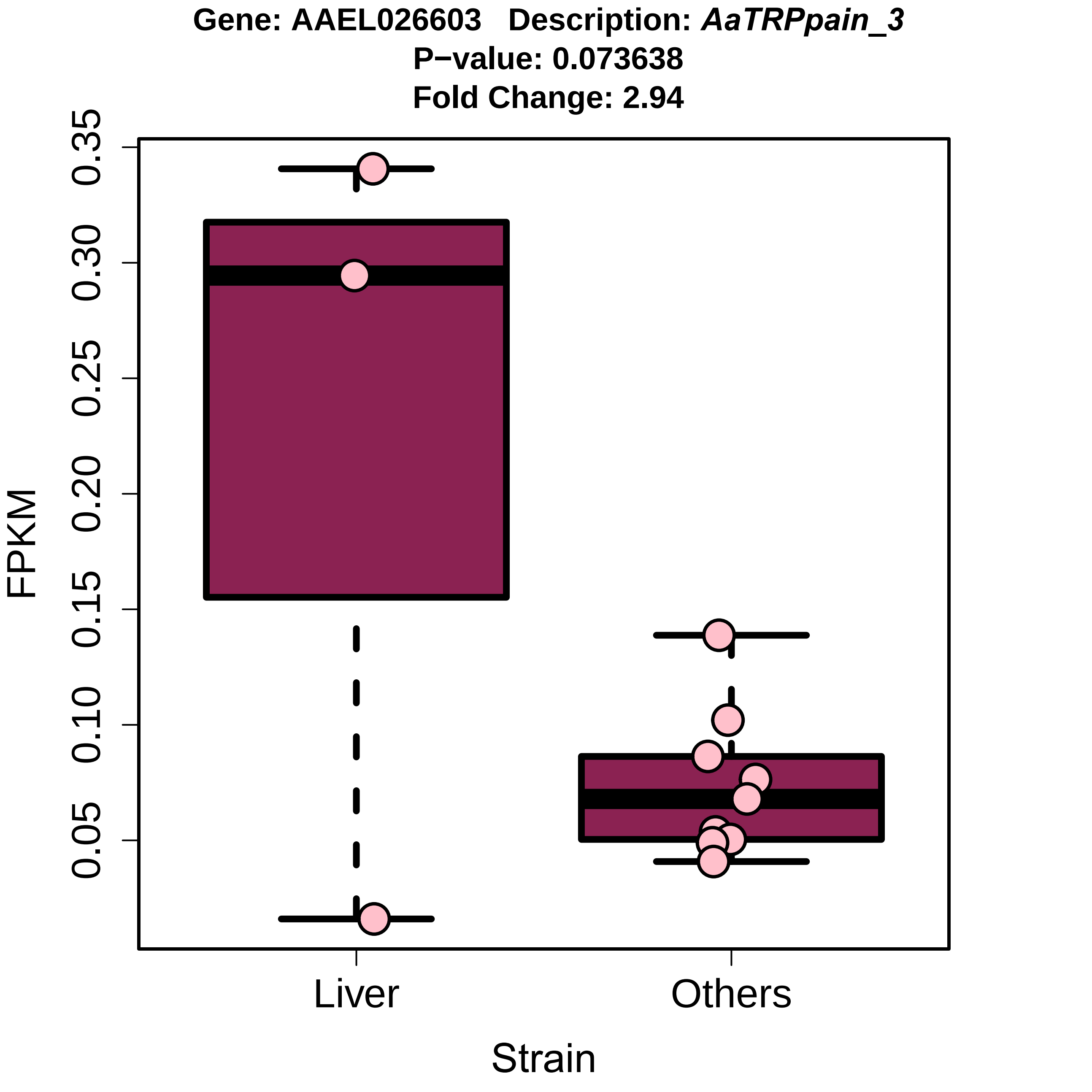

Gene: AAEL026603 Description: *AaTRPpain_3*
P−value: 0.073638
Fold Change: 2.94
FPKM
0.05
0.10
0.15
0.20
0.25
0.30
0.35
Liver
Others
Strain

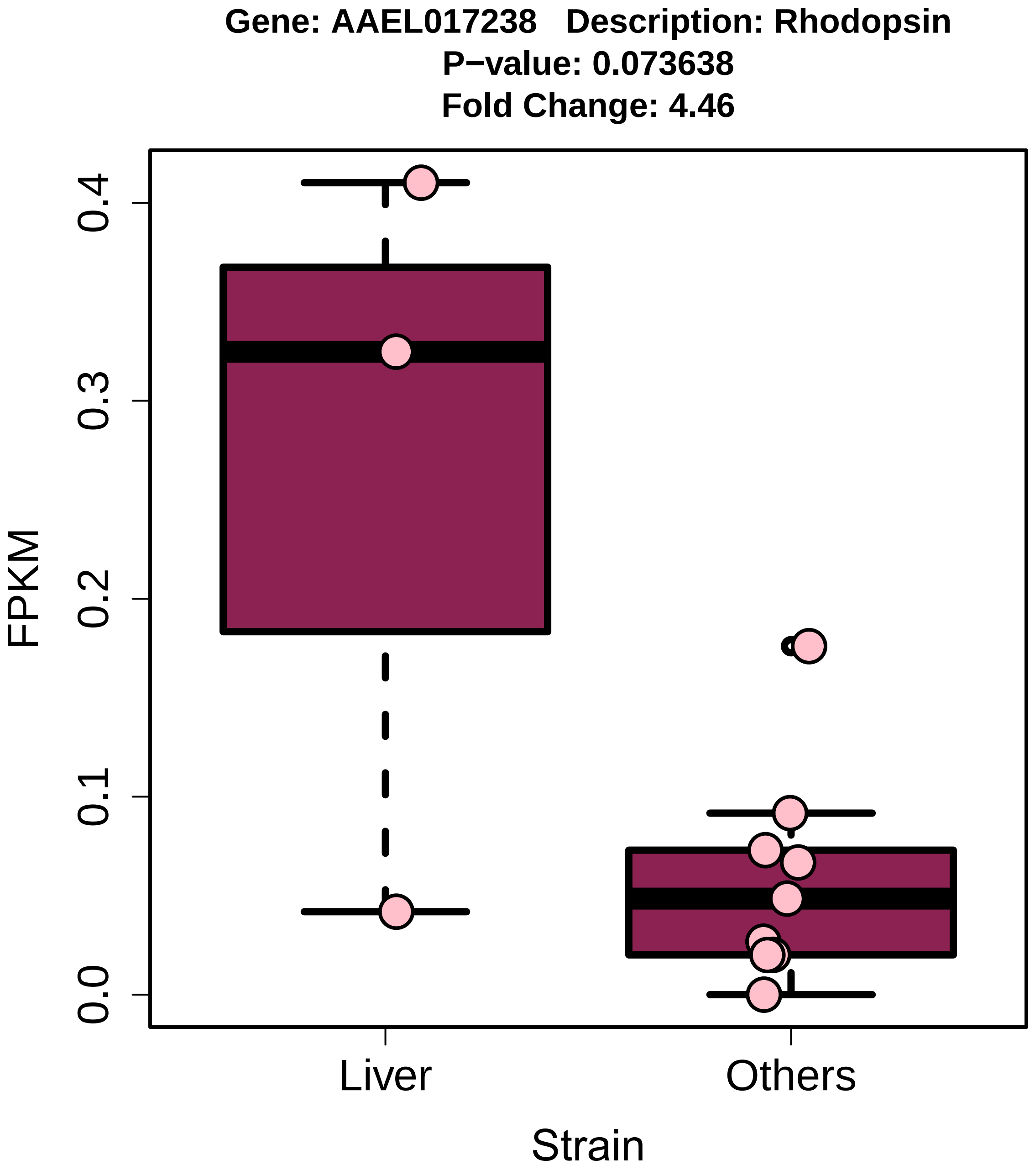
Gene: AAEL017238 Description: Rhodopsin
P−value: 0.073638
Fold Change: 4.46
FPKM
0.0
0.1
0.2
0.3
0.4
Liver
Others
Strain

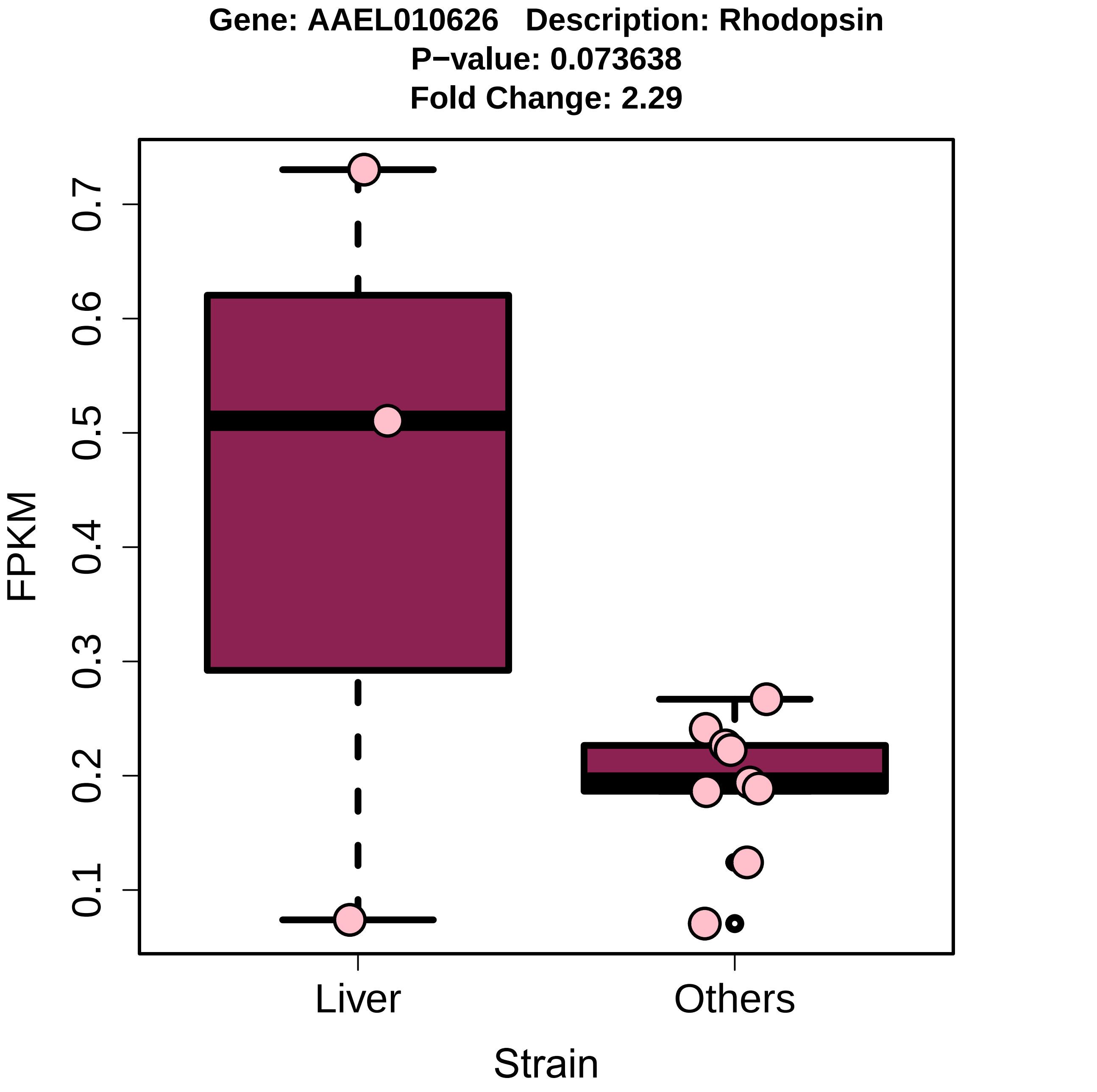
Gene: AAEL010626   Description: Rhodopsin
P−value: 0.073638
Fold Change: 2.29
FPKM
0.1
0.2
0.3
0.4
0.5
0.6
0.7
Liver
Others
Strain

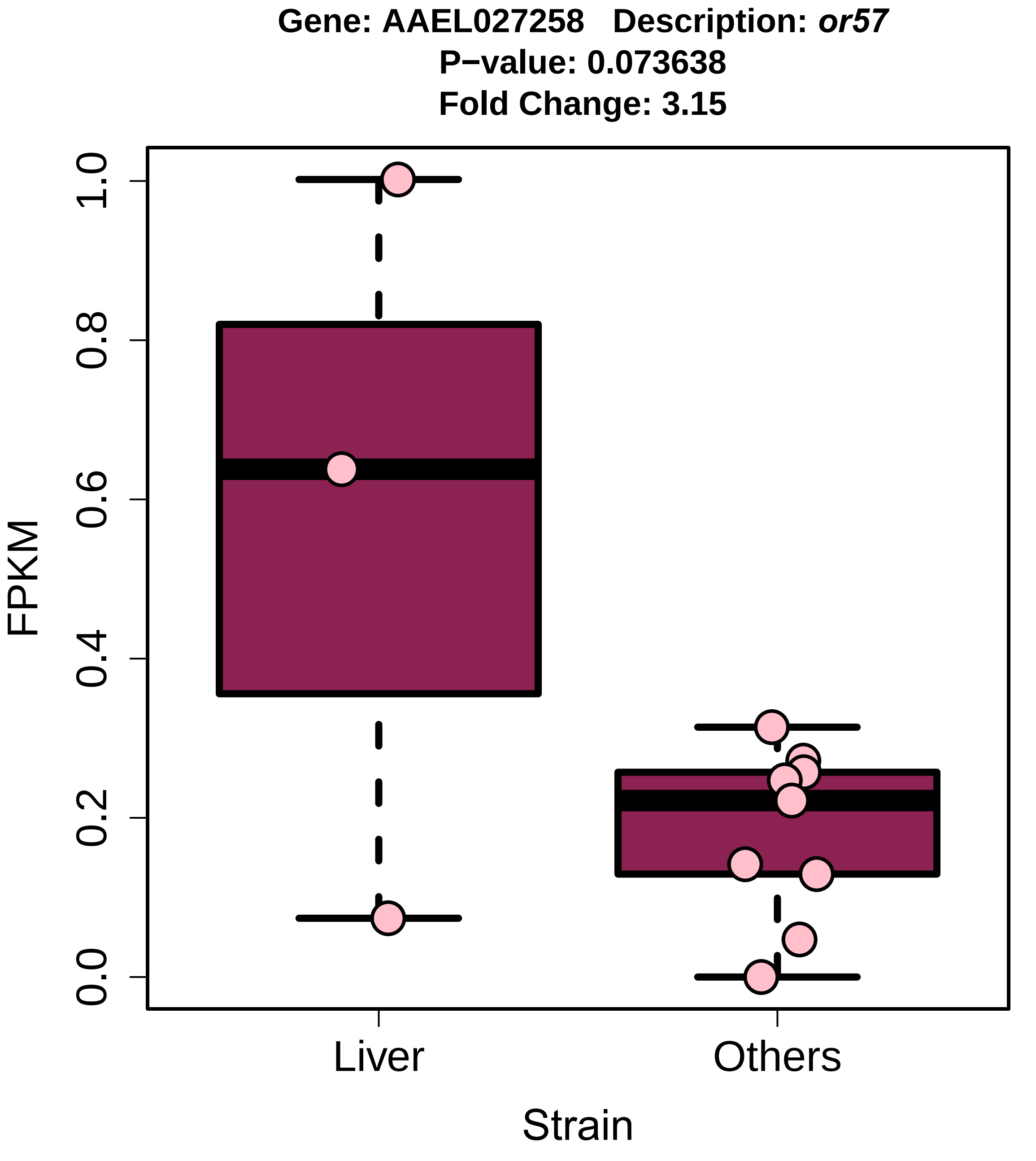
Gene: AAEL027258 Description: *or57*
P−value: 0.073638
Fold Change: 3.15
FPKM
0.0
0.2
0.4
0.6
0.8
1.0
Liver
Others
Strain

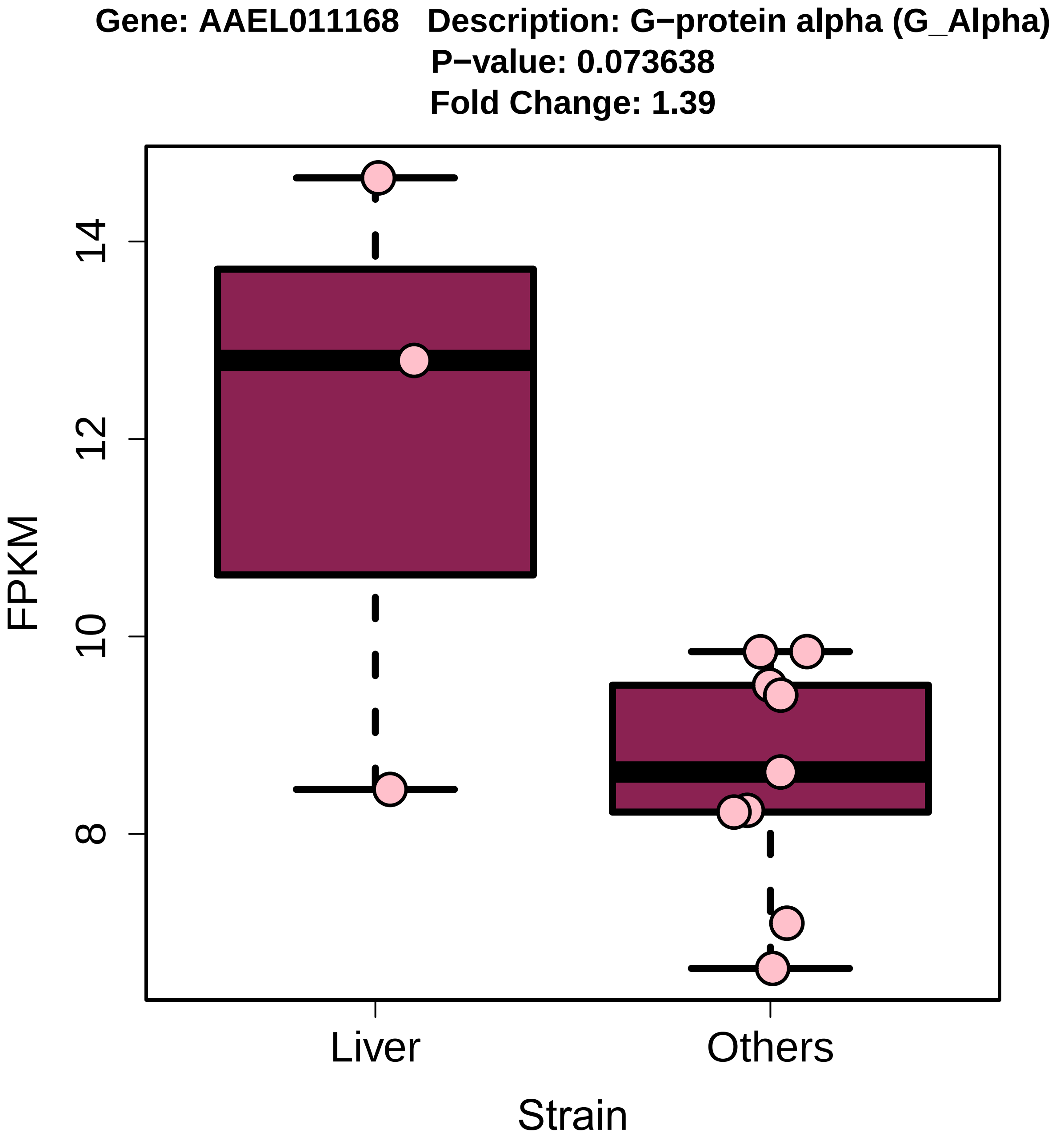
Gene: AAEL011168 Description: G−protein alpha (G_Alpha)
P−value: 0.073638
Fold Change: 1.39
FPKM
14
12
10
8
Liver
Others
Strain

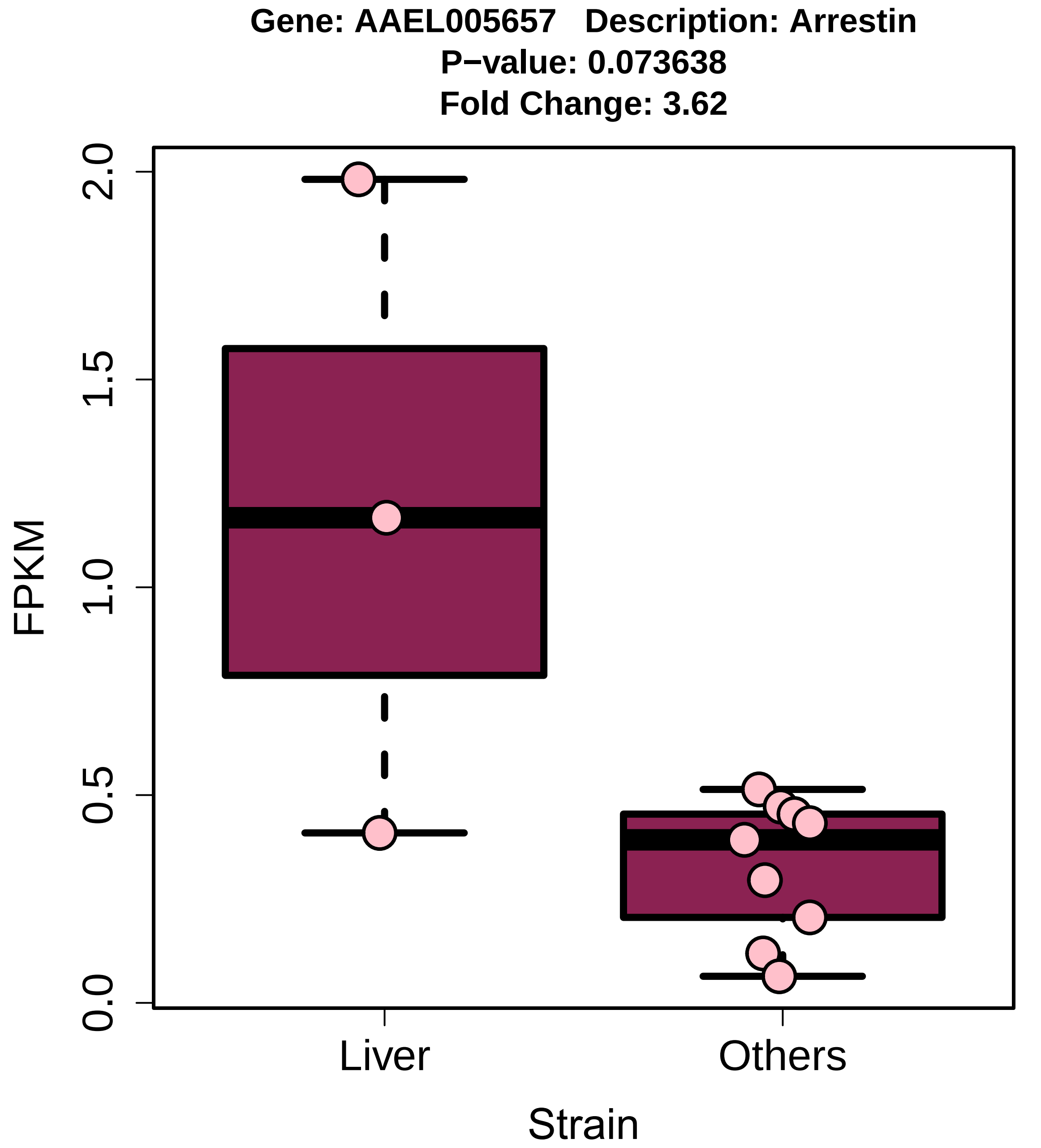

Gene: AAEL005657 Description: Arrestin
P−value: 0.073638
Fold Change: 3.62
FPKM
0.0
0.5
1.0
1.5
2.0
Liver
Others
Strain

Gene: AAEL006005 Description: *or9*
P−value: 0.073638
Fold Change: 4.76

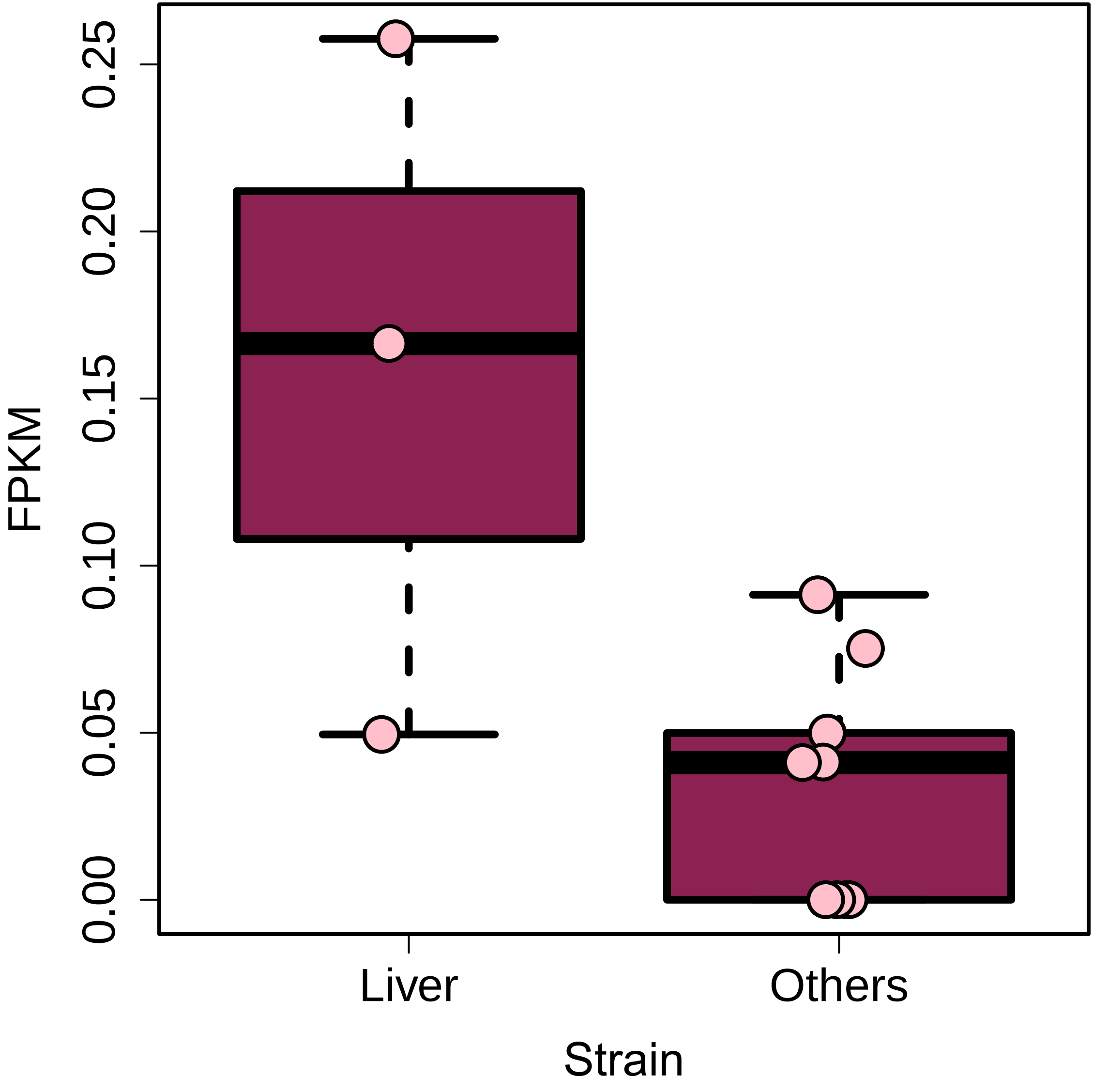

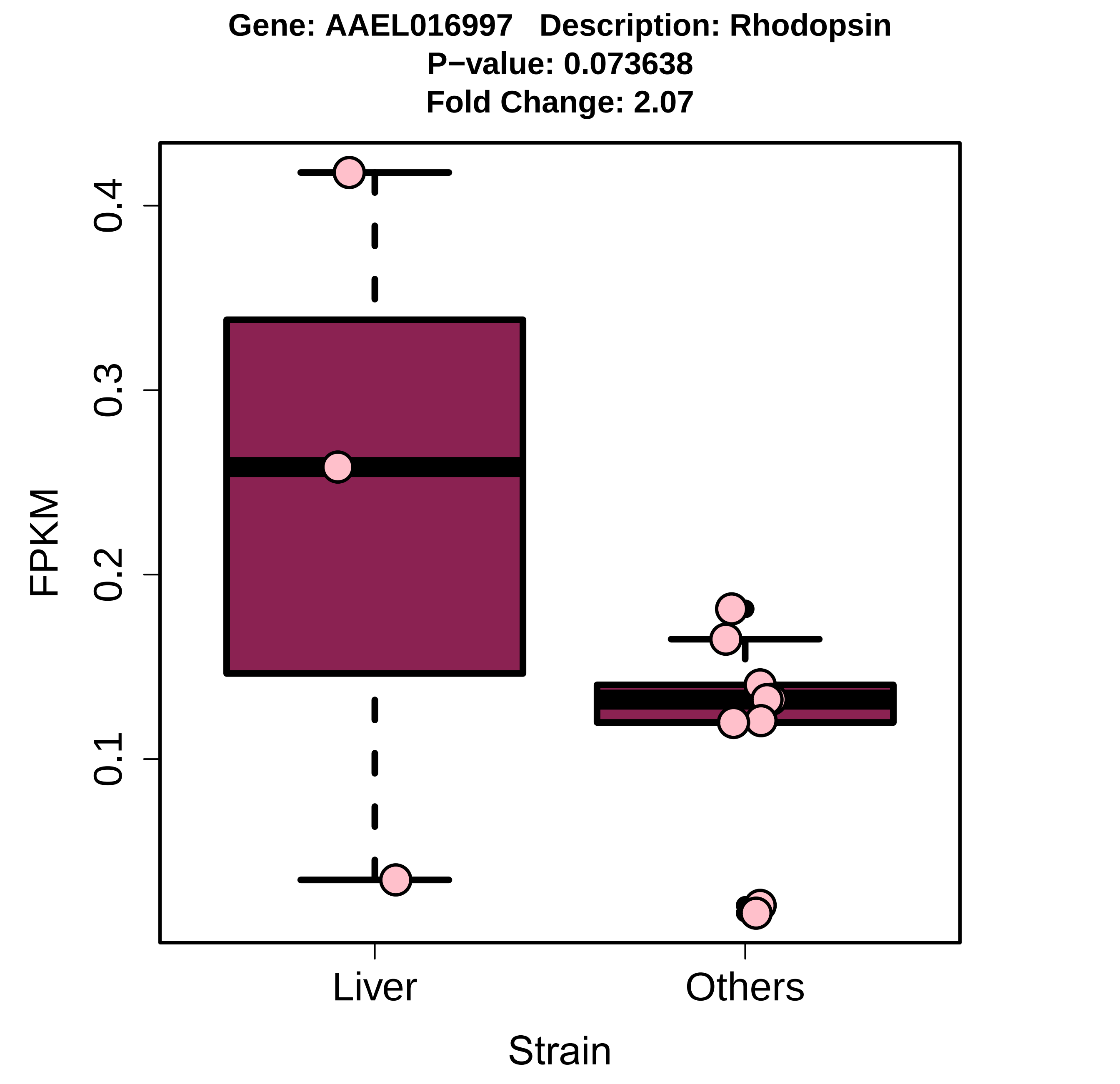
Gene: AAEL016997 Description: Rhodopsin
P−value: 0.073638
Fold Change: 2.07
FPKM
0.1
0.2
0.3
0.4
Liver
Others
Strain

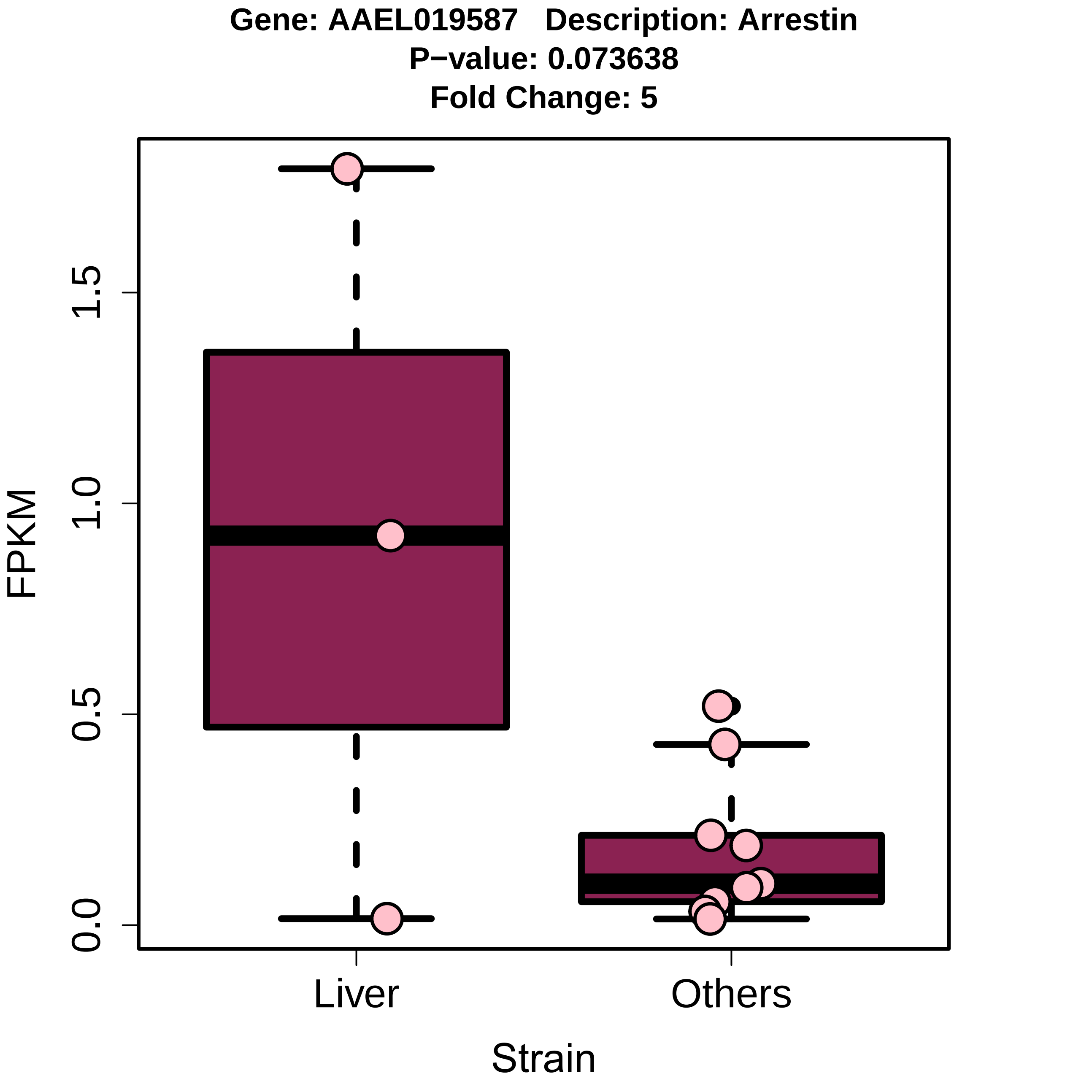
Gene: AAEL019587   Description: Arrestin
P−value: 0.073638
Fold Change: 5
FPKM
0.0
0.5
1.0
1.5
Liver
Others
Strain

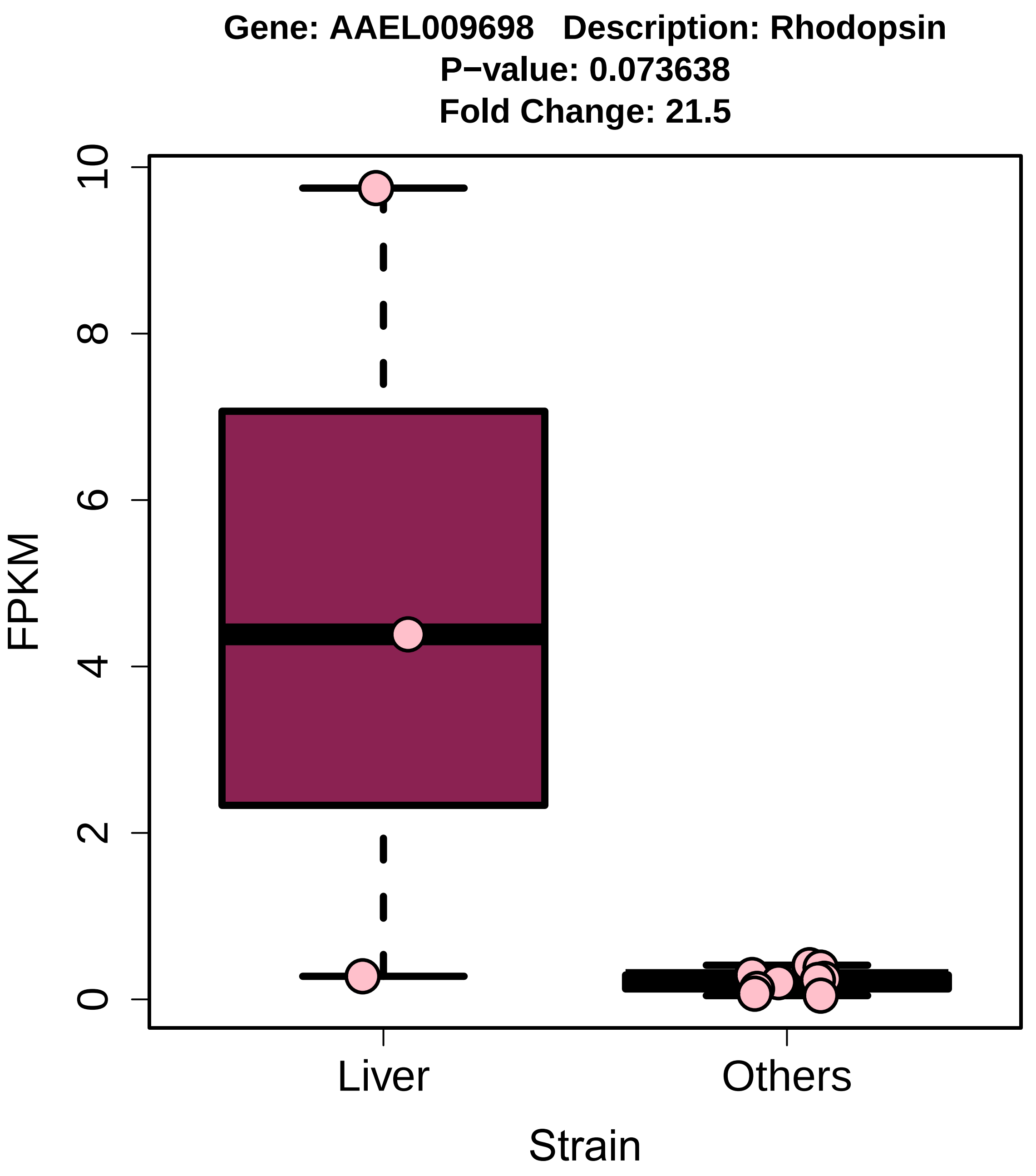

Gene: AAEL009698 Description: Rhodopsin
P−value: 0.073638
Fold Change: 21.5
FPKM
0
2
4
6
8
10
Liver
Others
Strain

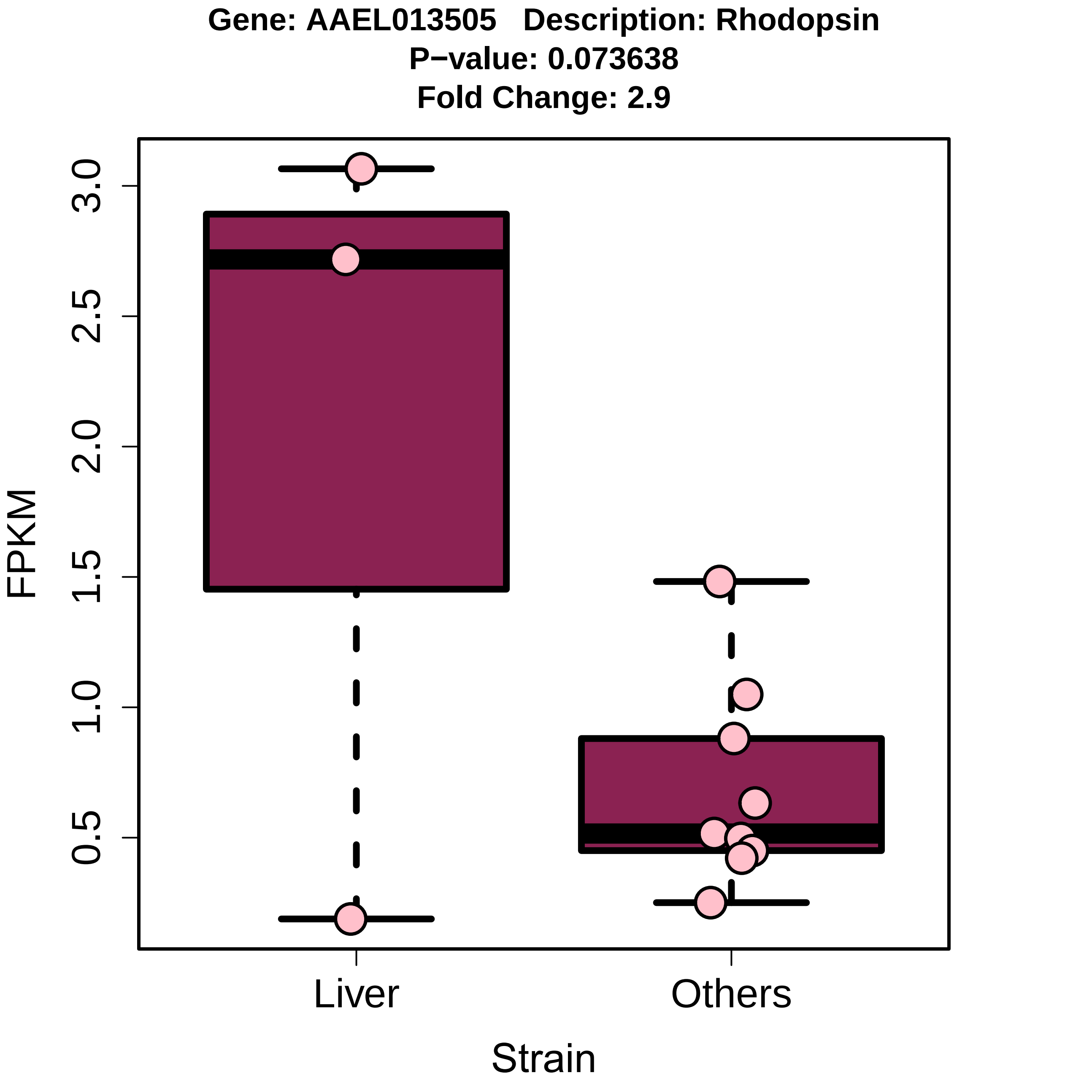
Gene: AAEL013505 Description: Rhodopsin
P−value: 0.073638
Fold Change: 2.9
FPKM
3.0
2.5
2.0
1.5
1.0
0.5
Liver
Others
Strain

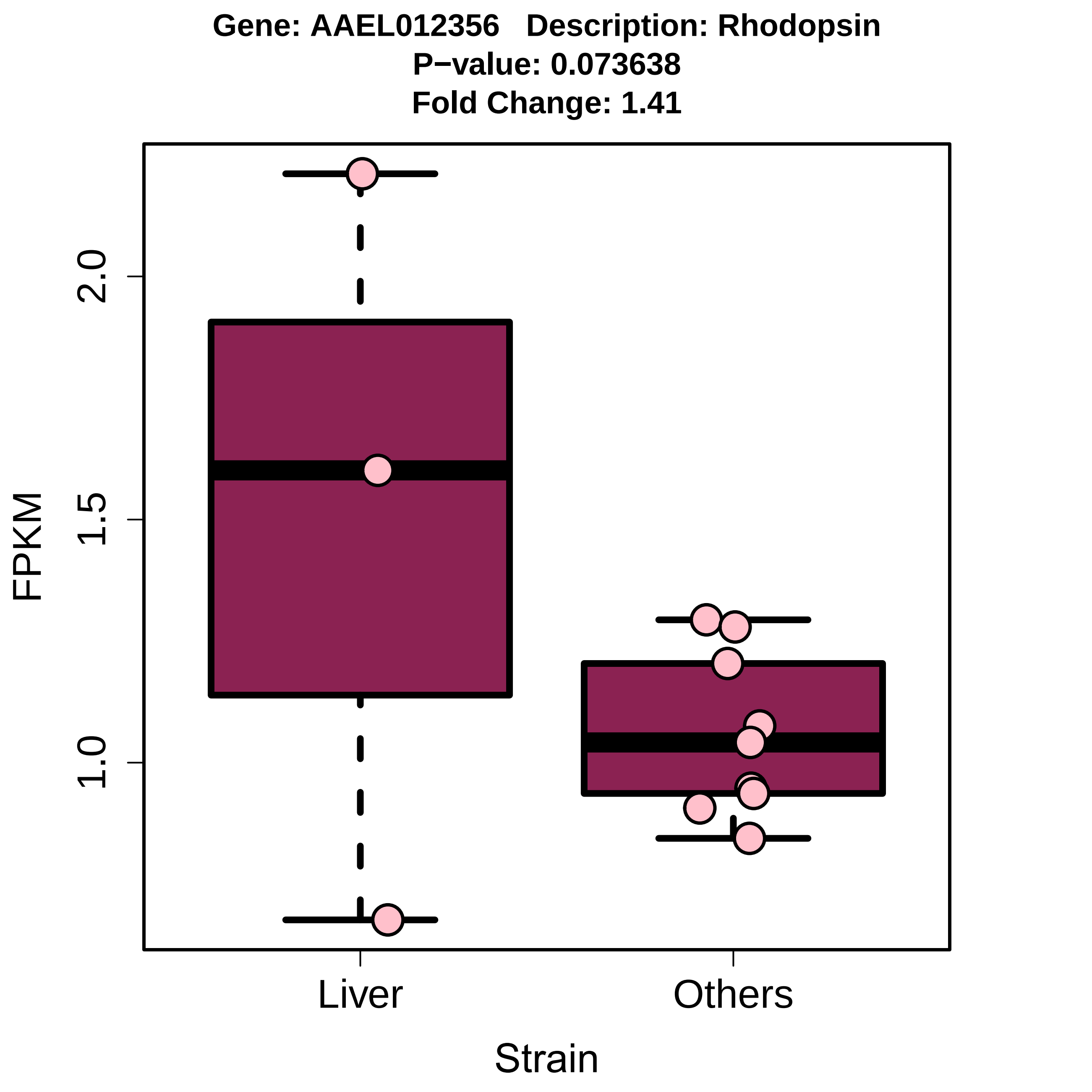

Gene: AAEL012356 Description: Rhodopsin
P−value: 0.073638
Fold Change: 1.41
FPKM
1.0
1.5
2.0
Liver
Others
Strain

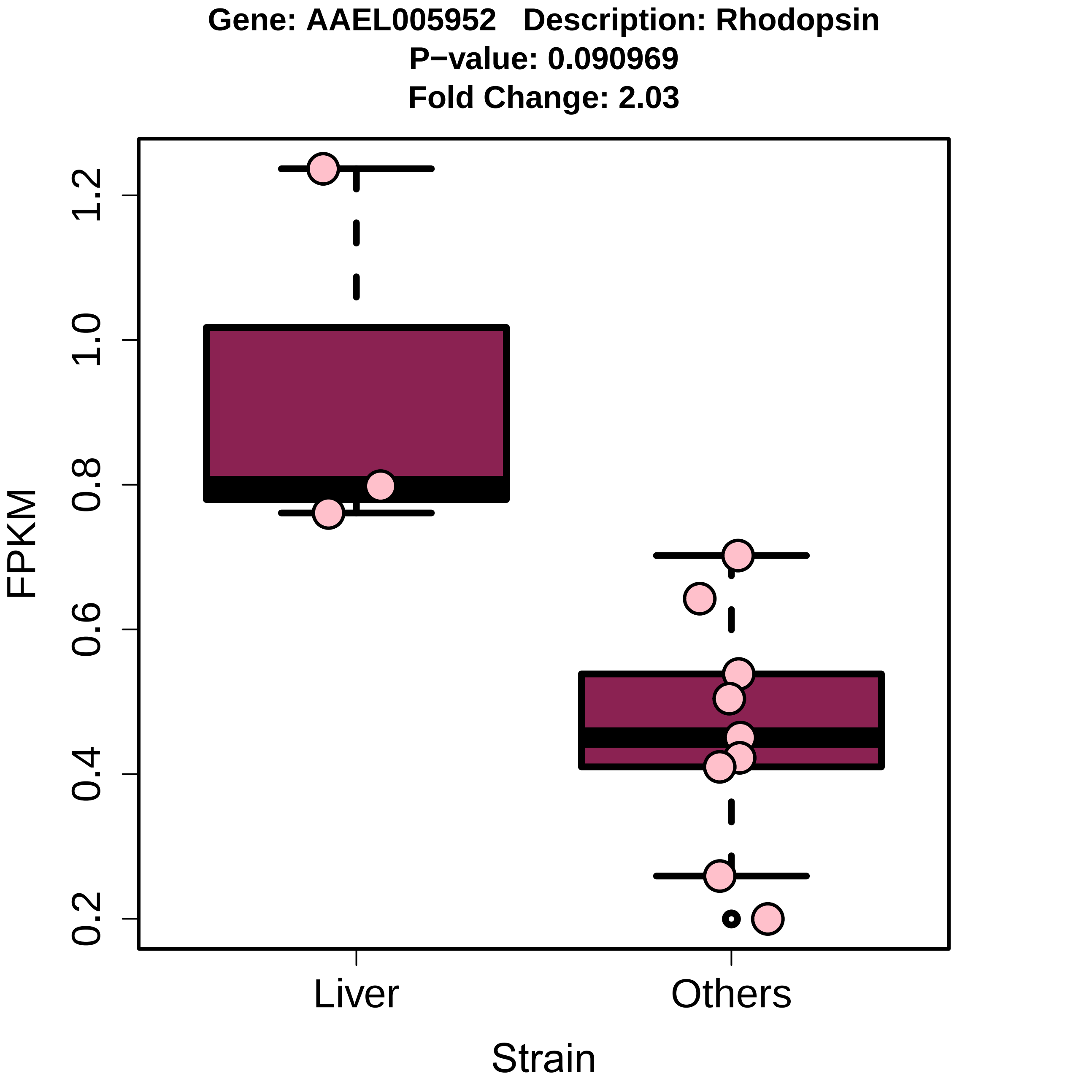

Gene: AAEL005952 Description: Rhodopsin
P−value: 0.090969
Fold Change: 2.03
FPKM
0.2
0.4
0.6
0.8
1.0
1.2
Liver
Others
Strain

Gene: AAEL006283   Description: Rhodopsin
P−value: 0.090969
Fold Change: 1.41

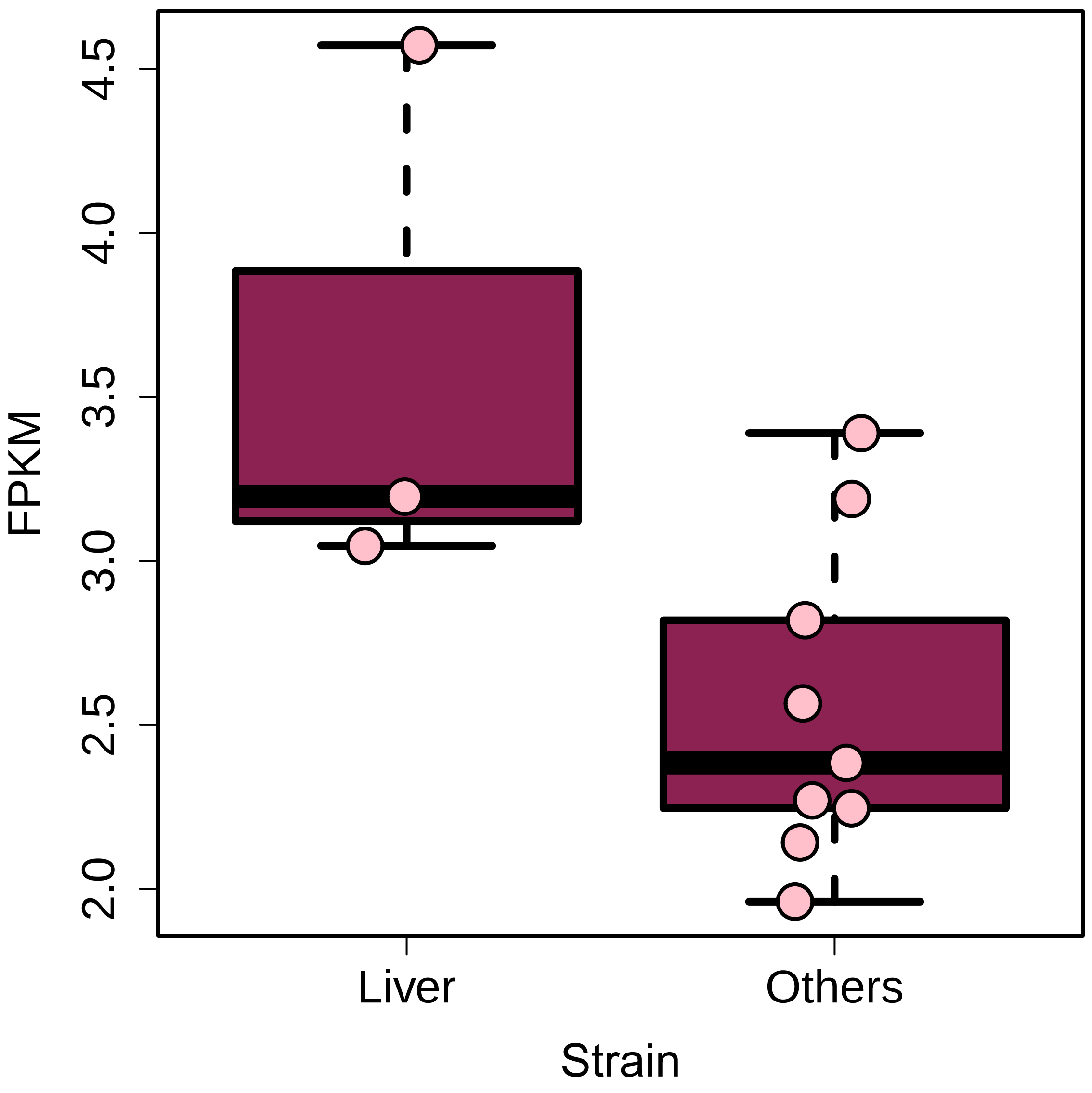

Gene: AAEL013198 Description: *ir75i*
P−value: 0.090969
Fold Change: 1.82

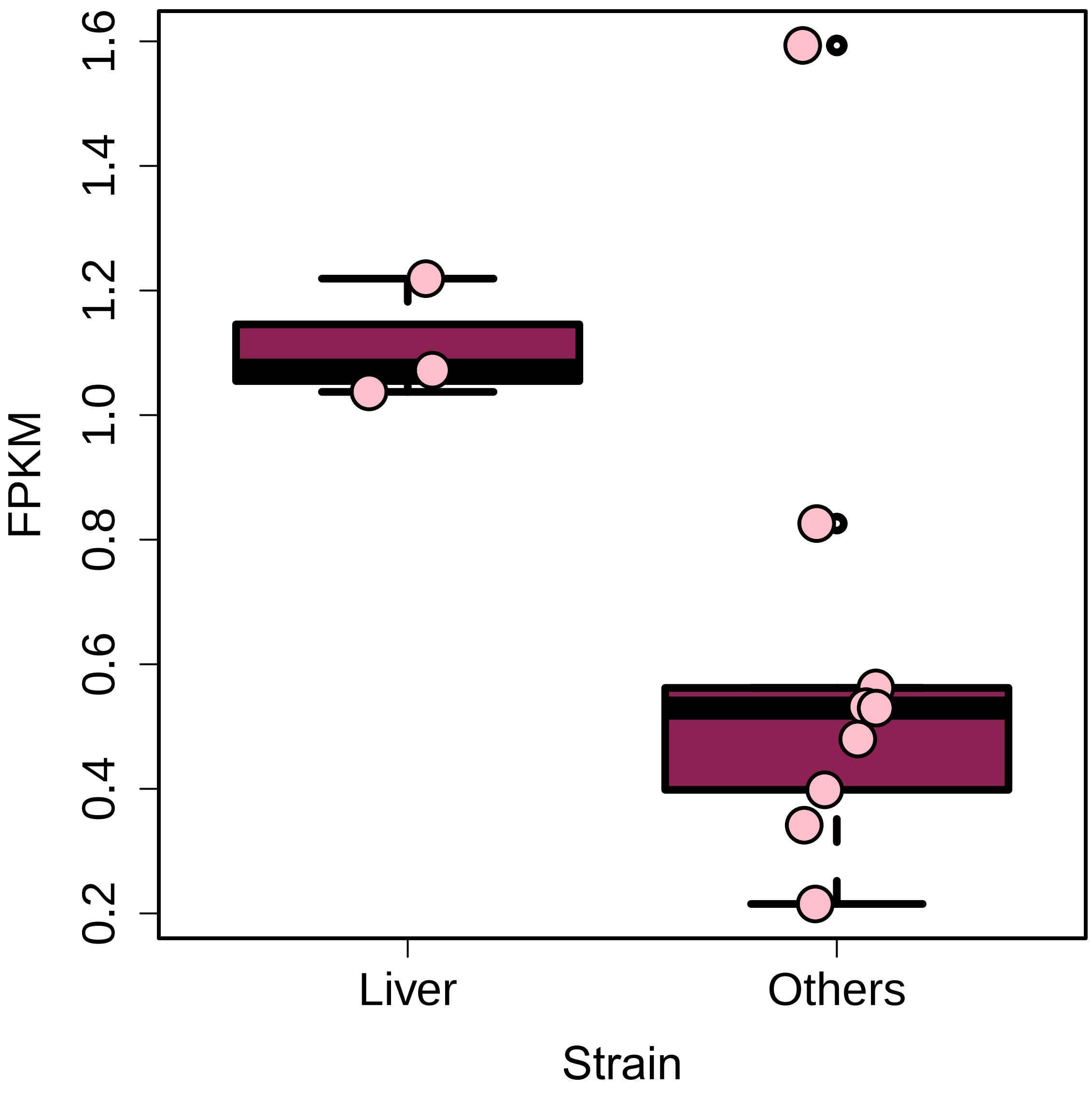

Supplement: Supplementary file 5 [file Data_Sheet_2.pdf]
