# Supplementary material for: Olfaction-Related Gene Expression in the Antennae of Female Mosquitoes From Common Aedes aegypti Laboratory Strains
Source: Front Physiol. 2021 Aug 23;12:668236. doi: 10.3389/fphys.2021.668236 (PMC8419471; doi:10.3389/fphys.2021.668236)
Supplement: Supplementary file 6 [file Data_Sheet_3.pdf]

Supplemental File 7. Boxplots of top 20 olfaction-associated genes expressed differentially in CR compared to other strains. Boxplots represent interquartile range of expression from CR (n=3) versus the other three strains, whiskers represent first and fourth quartiles, and solid lines in boxes represent median expression values. Circles represent expression values from individual samples. Differential expression was determined using chi-squared goodness of fit tests, and $p<0.05$ represent significantly different genes.

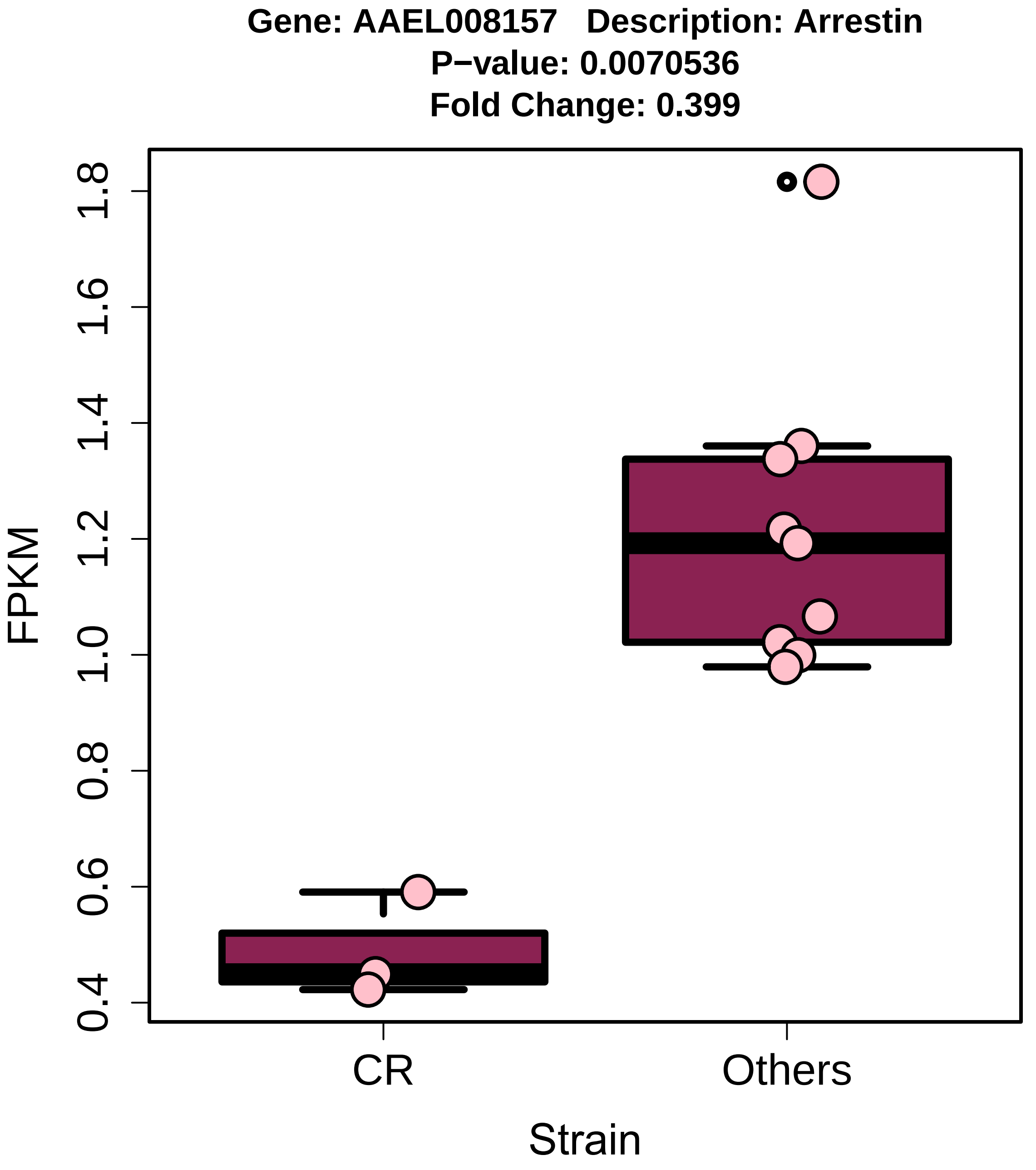

Gene: AAEL008157 Description: Arrestin
P−value: 0.0070536
Fold Change: 0.399
FPKM
0.4
0.6
0.8
1.0
1.2
1.4
1.6
1.8
CR
Others
Strain

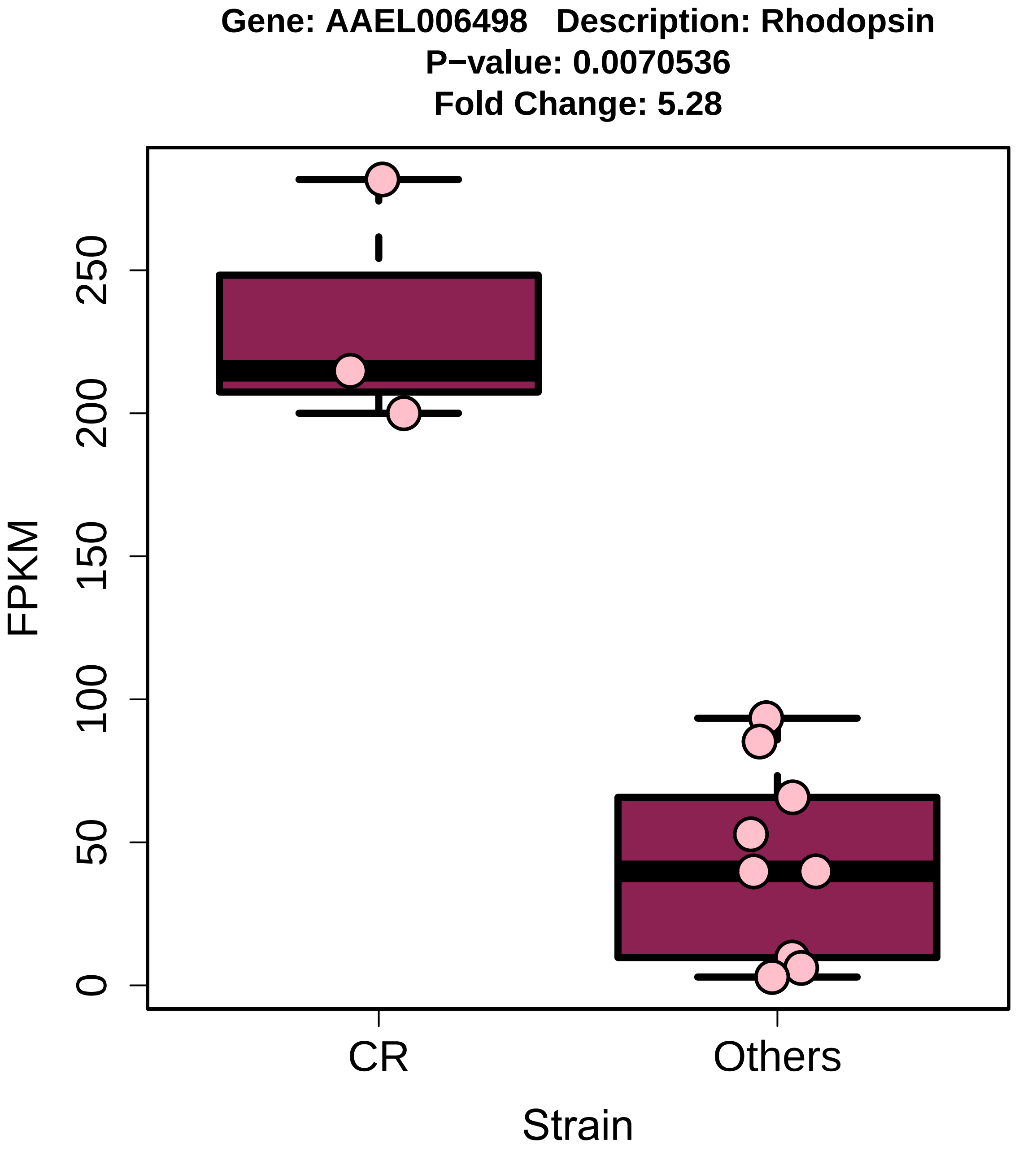

Gene: AAEL006498   Description: Rhodopsin
P−value: 0.0070536
Fold Change: 5.28
FPKM
0
50
100
150
200
250
CR
Others
Strain

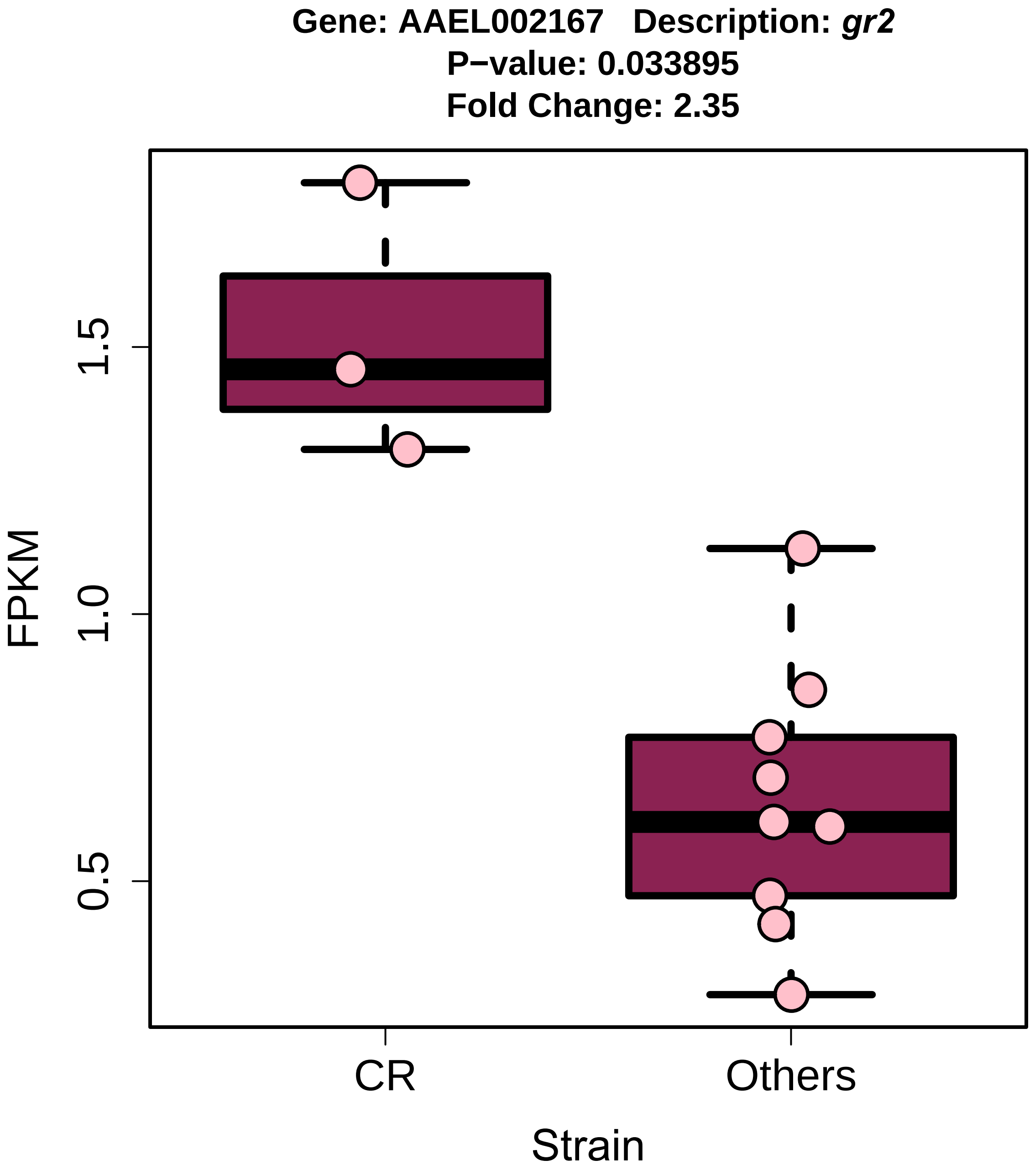

Gene: AAEL002167 Description: *gr2*
P−value: 0.033895
Fold Change: 2.35
FPKM
1.5
1.0
0.5
CR
Others
Strain

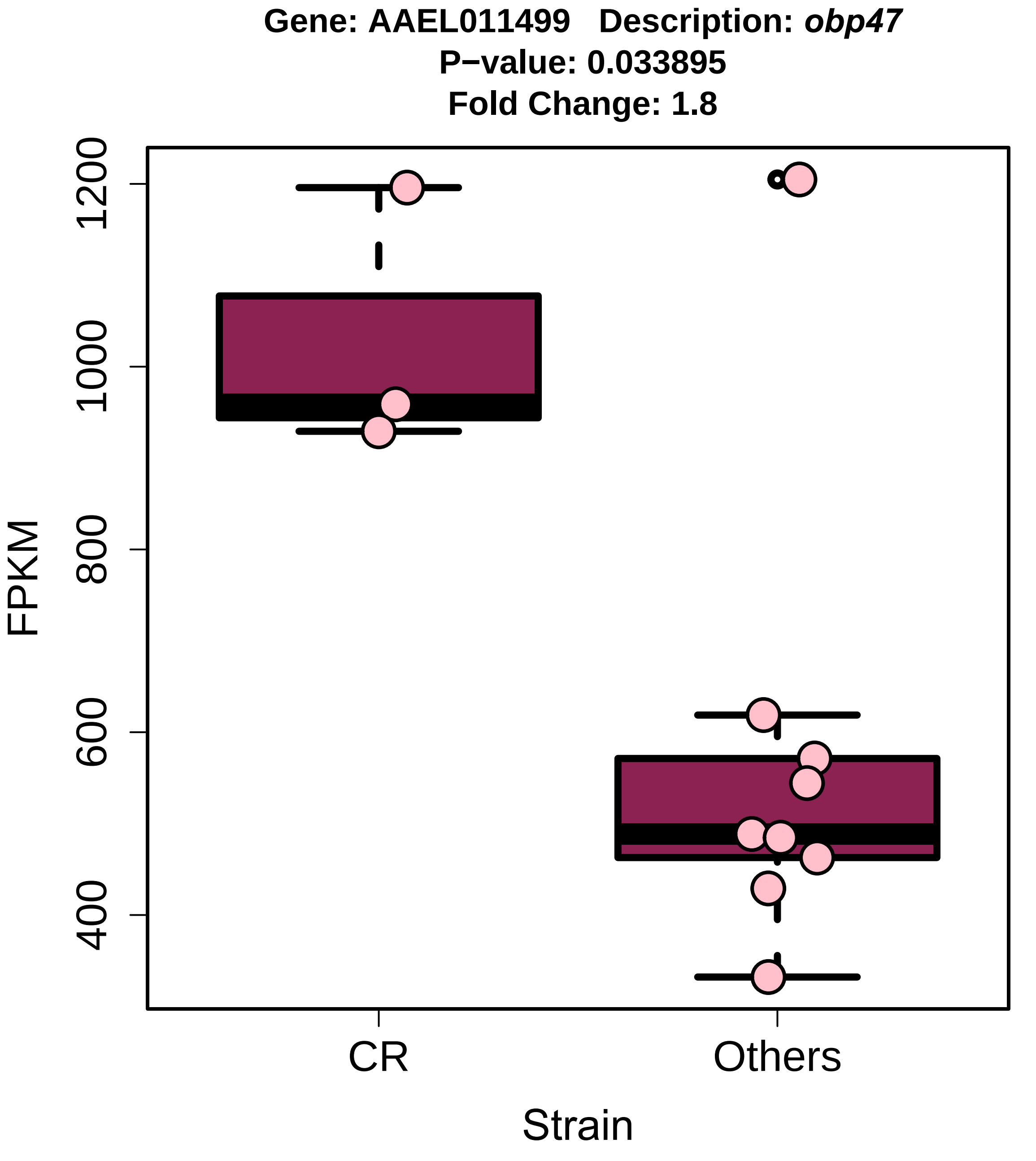

Gene: AAEL011499 Description: *obp47*
P−value: 0.033895
Fold Change: 1.8
1200
1000
800
600
400
FPKM
CR
Others
Strain

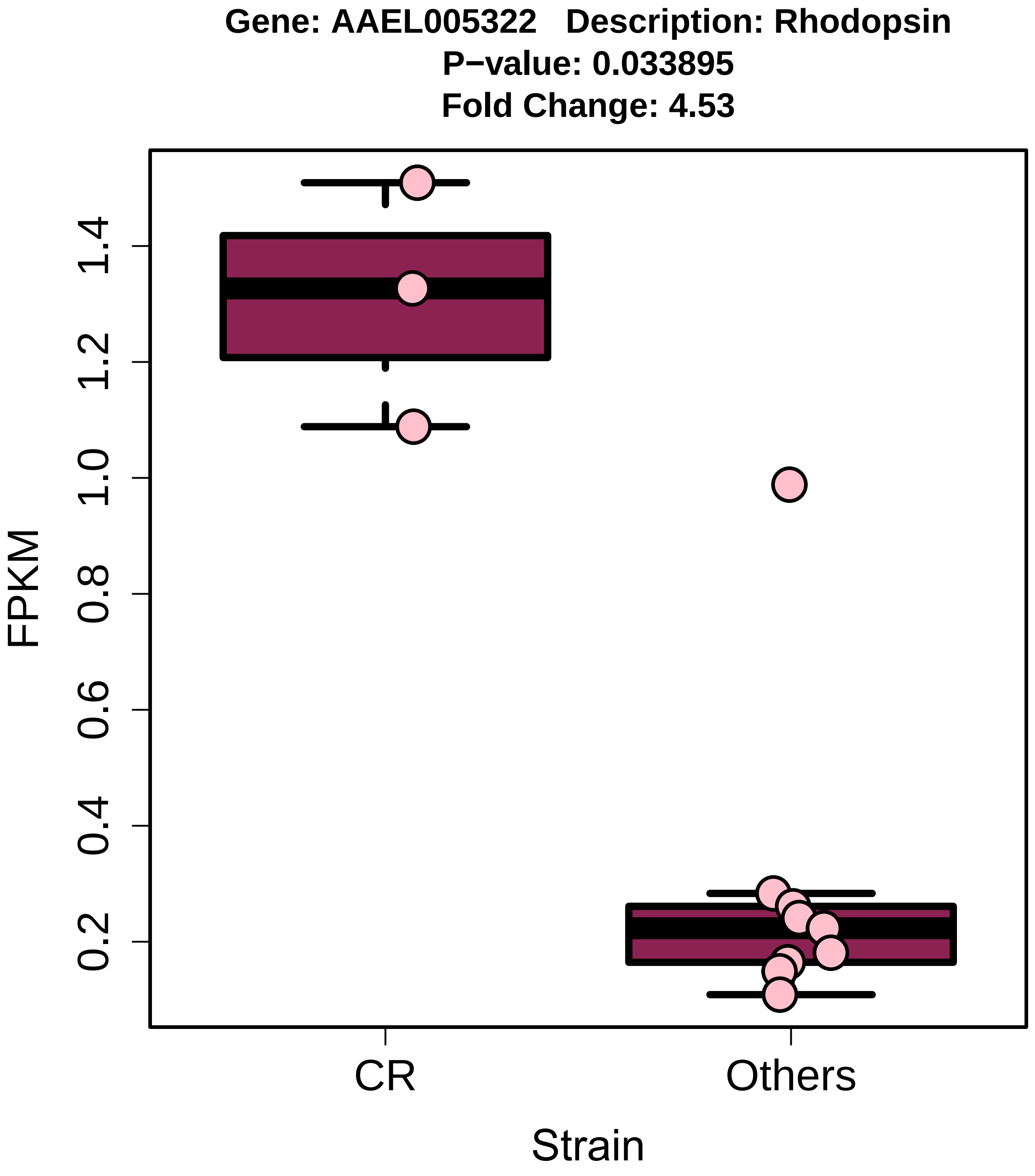
Gene: AAEL005322   Description: Rhodopsin
P−value: 0.033895
Fold Change: 4.53
FPKM
0.2
0.4
0.6
0.8
1.0
1.2
1.4
CR
Others
Strain

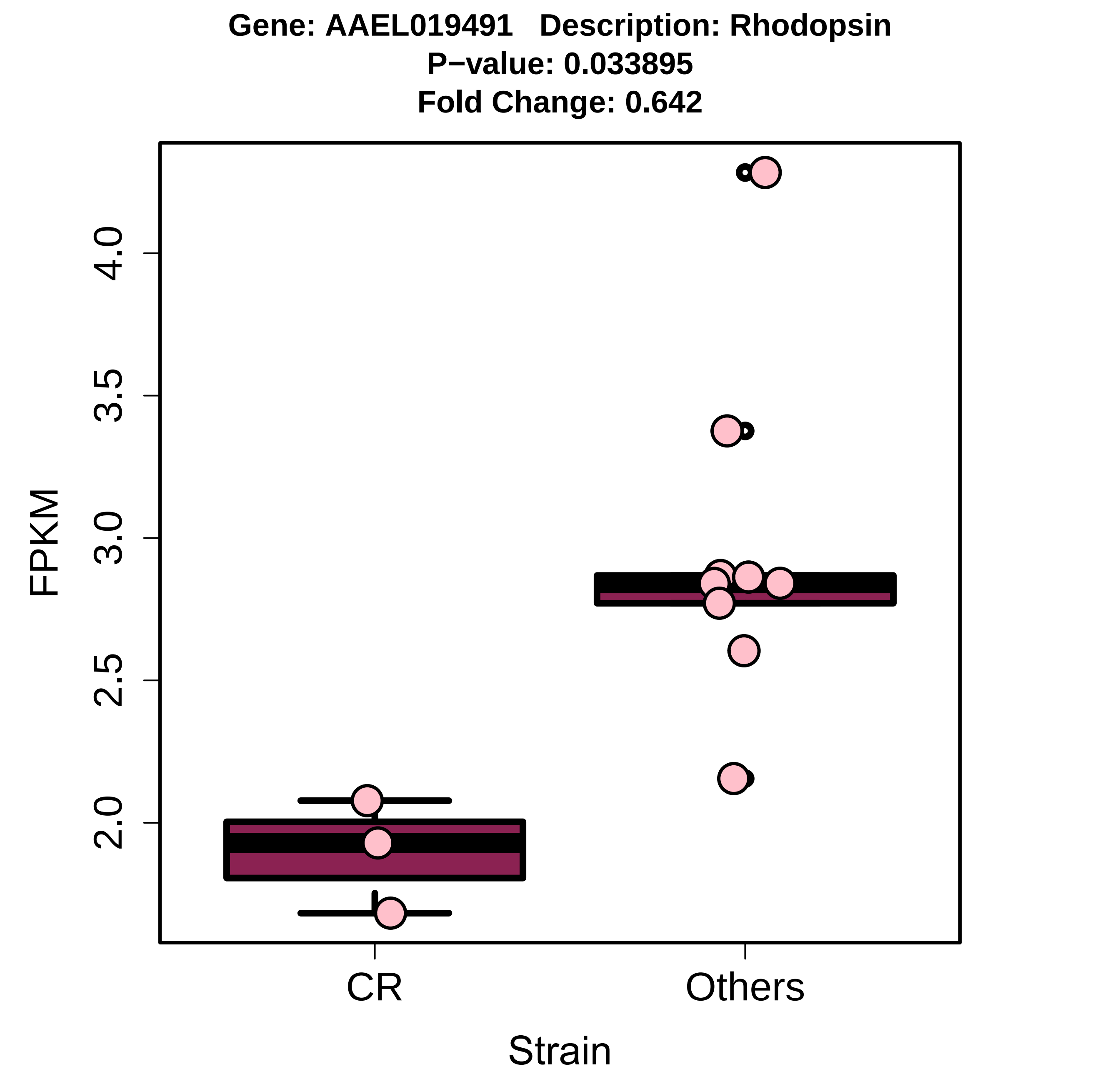
Gene: AAEL019491   Description: Rhodopsin
P−value: 0.033895
Fold Change: 0.642
FPKM
4.0
3.5
3.0
2.5
2.0
CR
Others
Strain

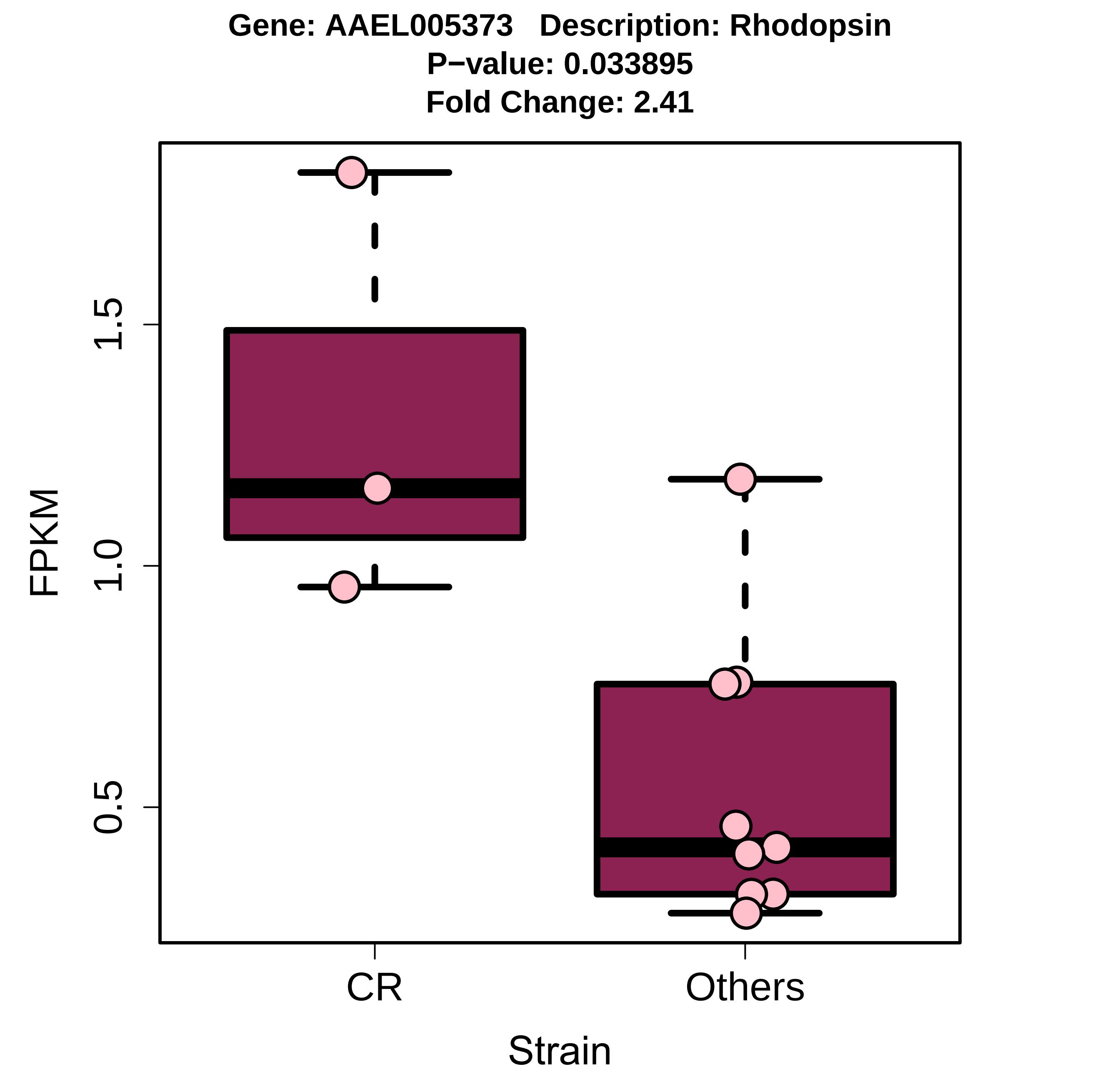
Gene: AAEL005373 Description: Rhodopsin
P−value: 0.033895
Fold Change: 2.41
FPKM
1.5
1.0
0.5
CR
Others
Strain

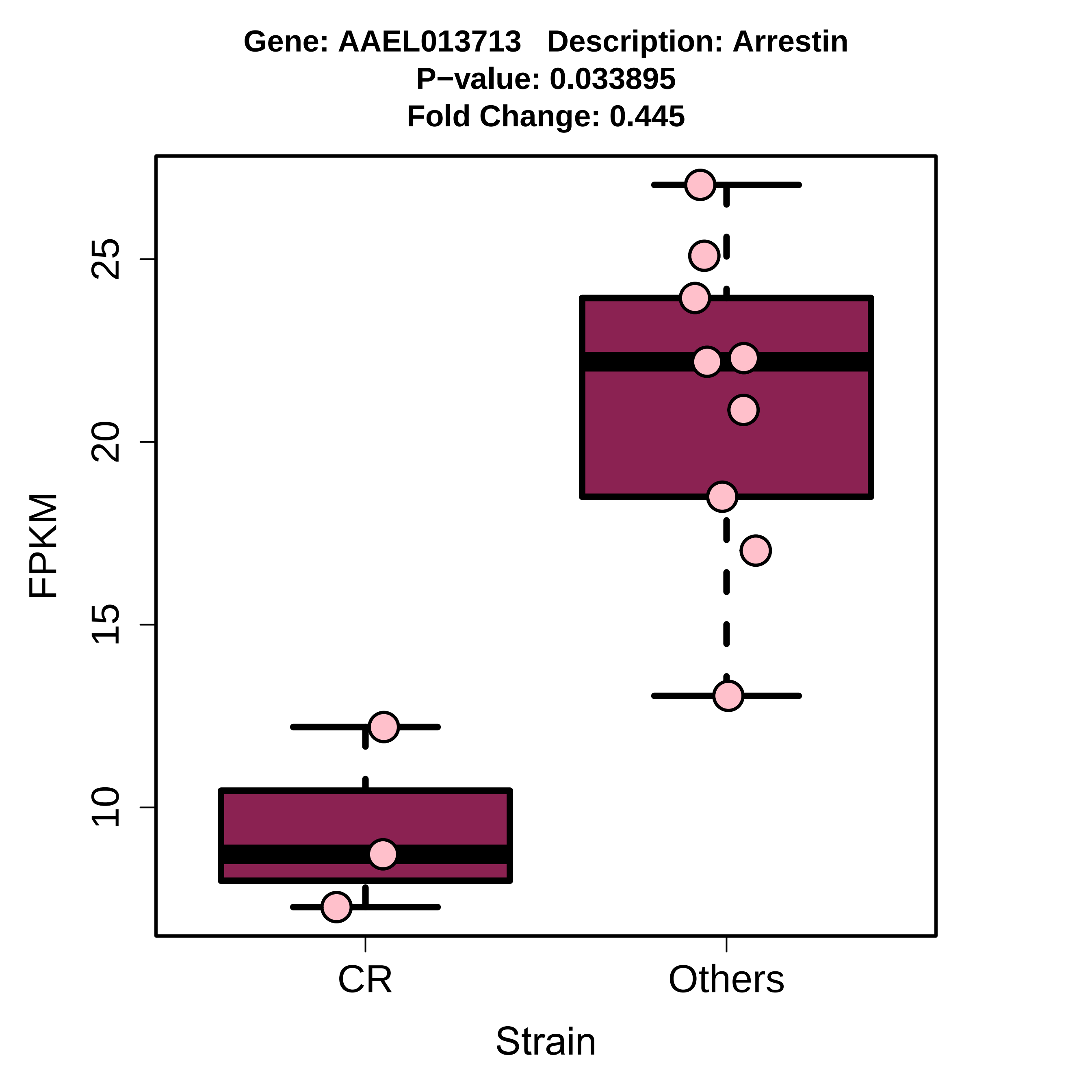

Gene: AAEL013713   Description: Arrestin
P−value: 0.033895
Fold Change: 0.445
FPKM
25
20
15
10
CR
Others
Strain

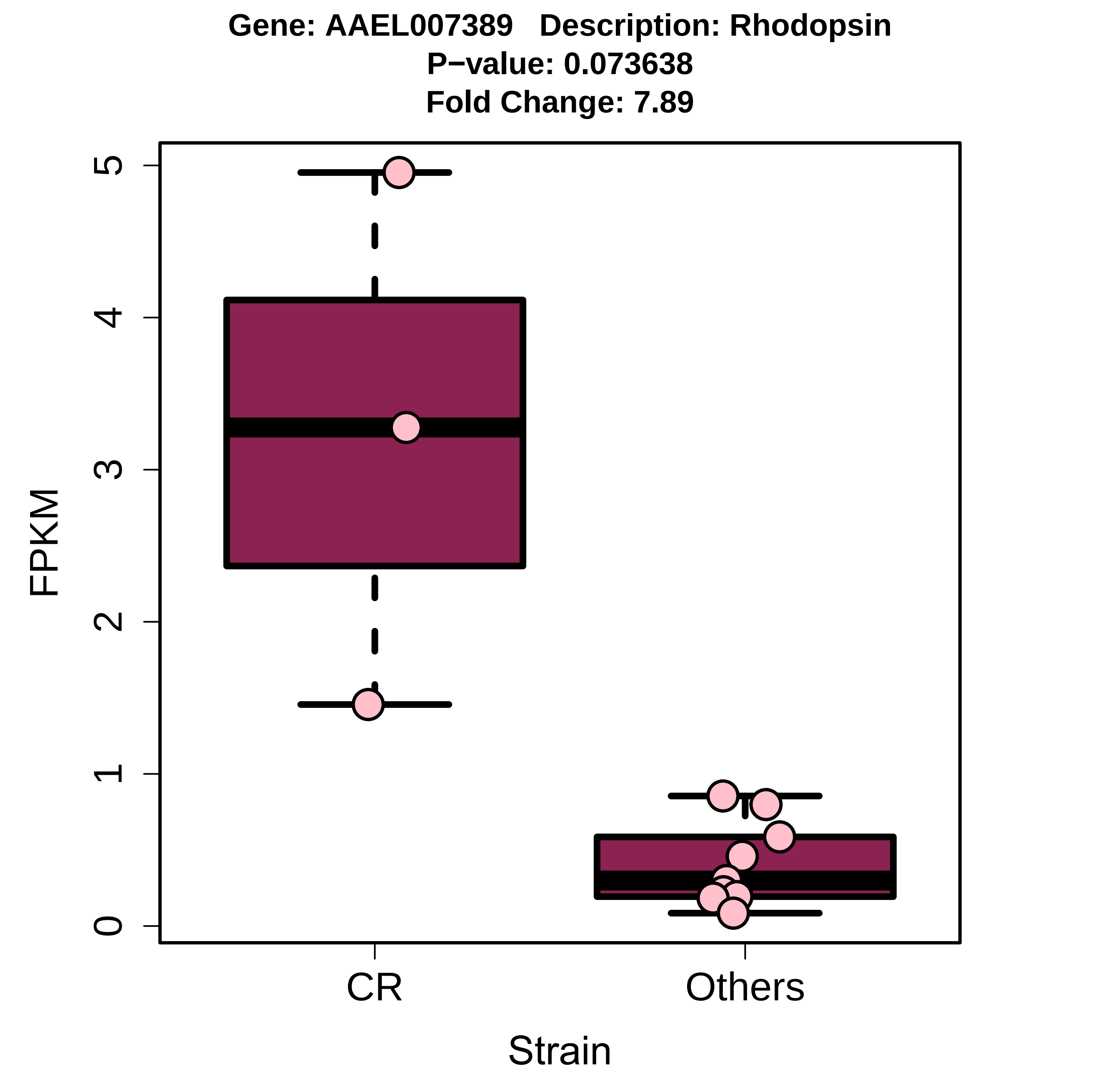

Gene: AAEL007389 Description: Rhodopsin
P−value: 0.073638
Fold Change: 7.89
FPKM
0
1
2
3
4
5
CR
Others
Strain

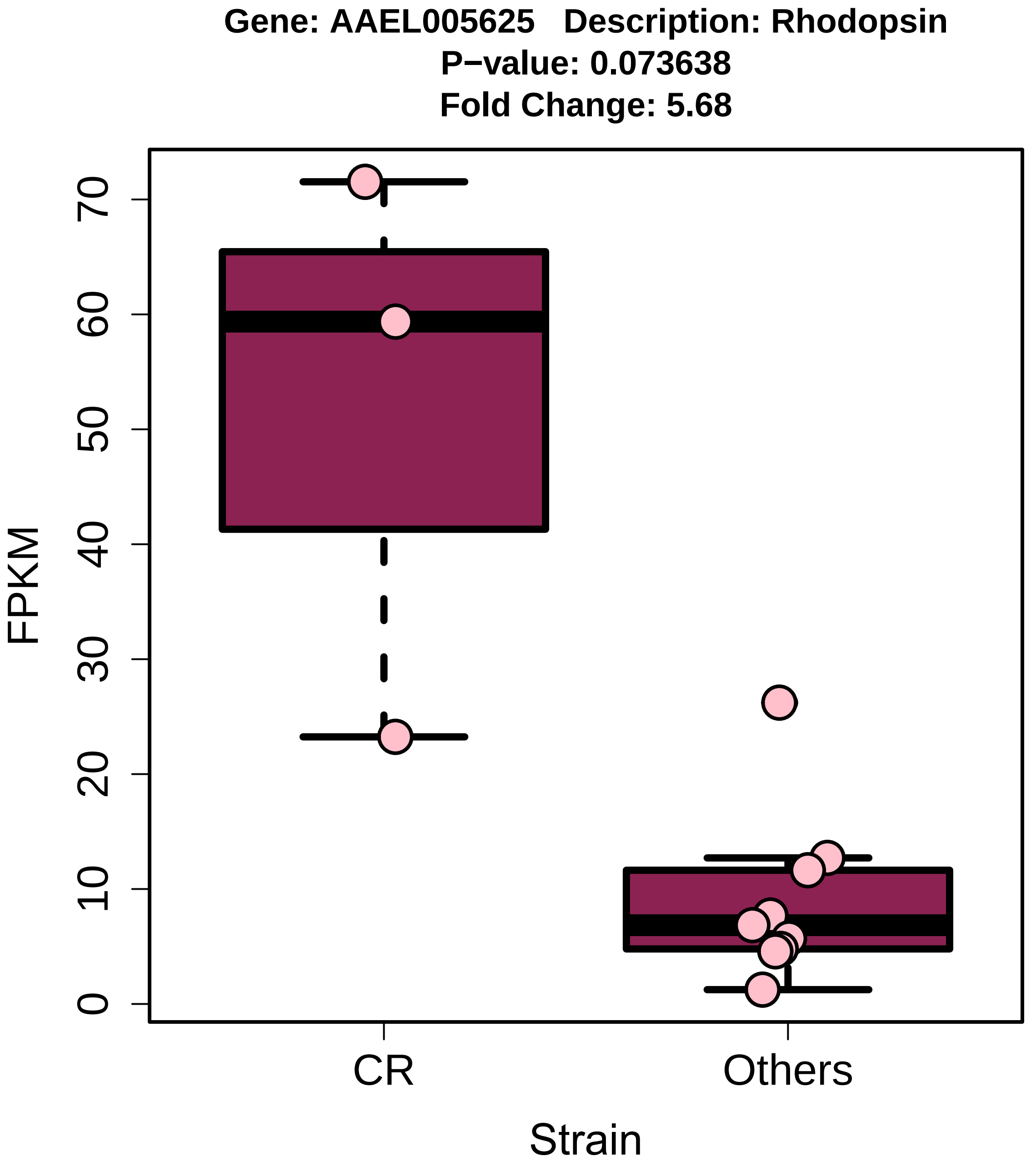
Gene: AAEL005625 Description: Rhodopsin
P−value: 0.073638
Fold Change: 5.68
FPKM
0
10
20
30
40
50
60
70
CR
Others
Strain

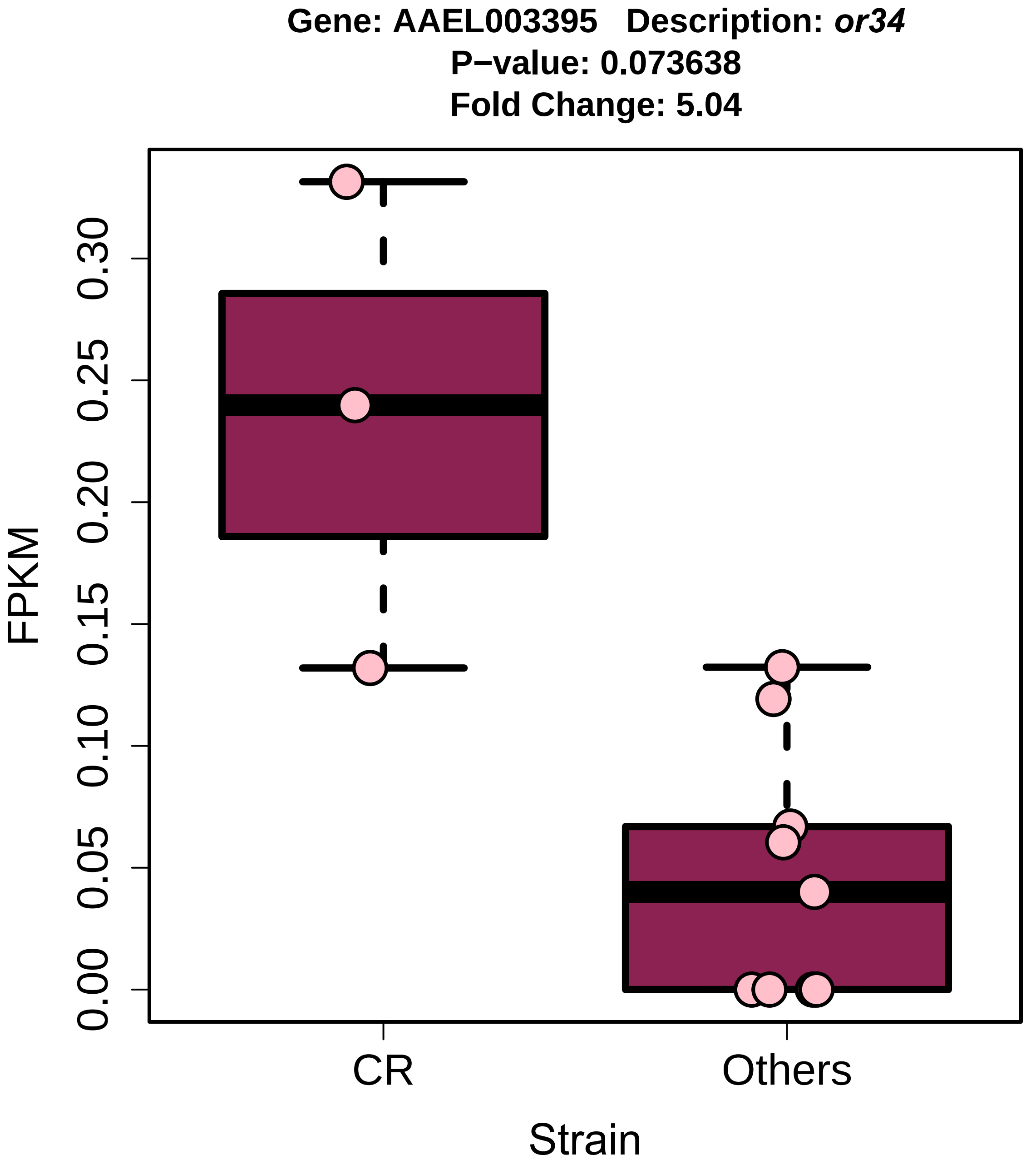
Gene: AAEL003395   Description: or34
P−value: 0.073638
Fold Change: 5.04
FPKM
0.00
0.05
0.10
0.15
0.20
0.25
0.30
CR
Others
Strain

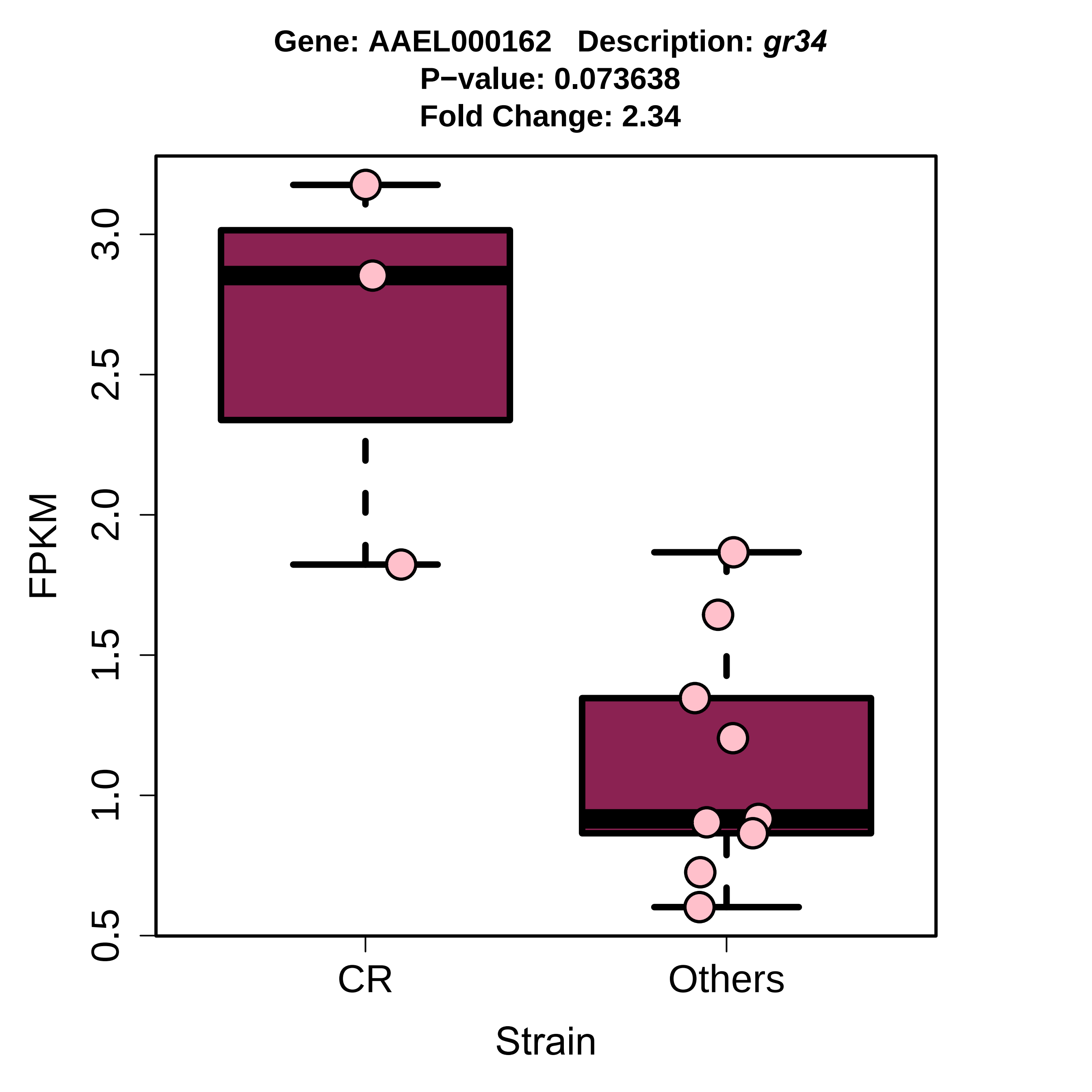
Gene: AAEL000162 Description: *gr34*
P−value: 0.073638
Fold Change: 2.34
FPKM
3.0
2.5
2.0
1.5
1.0
0.5
CR
Others
Strain

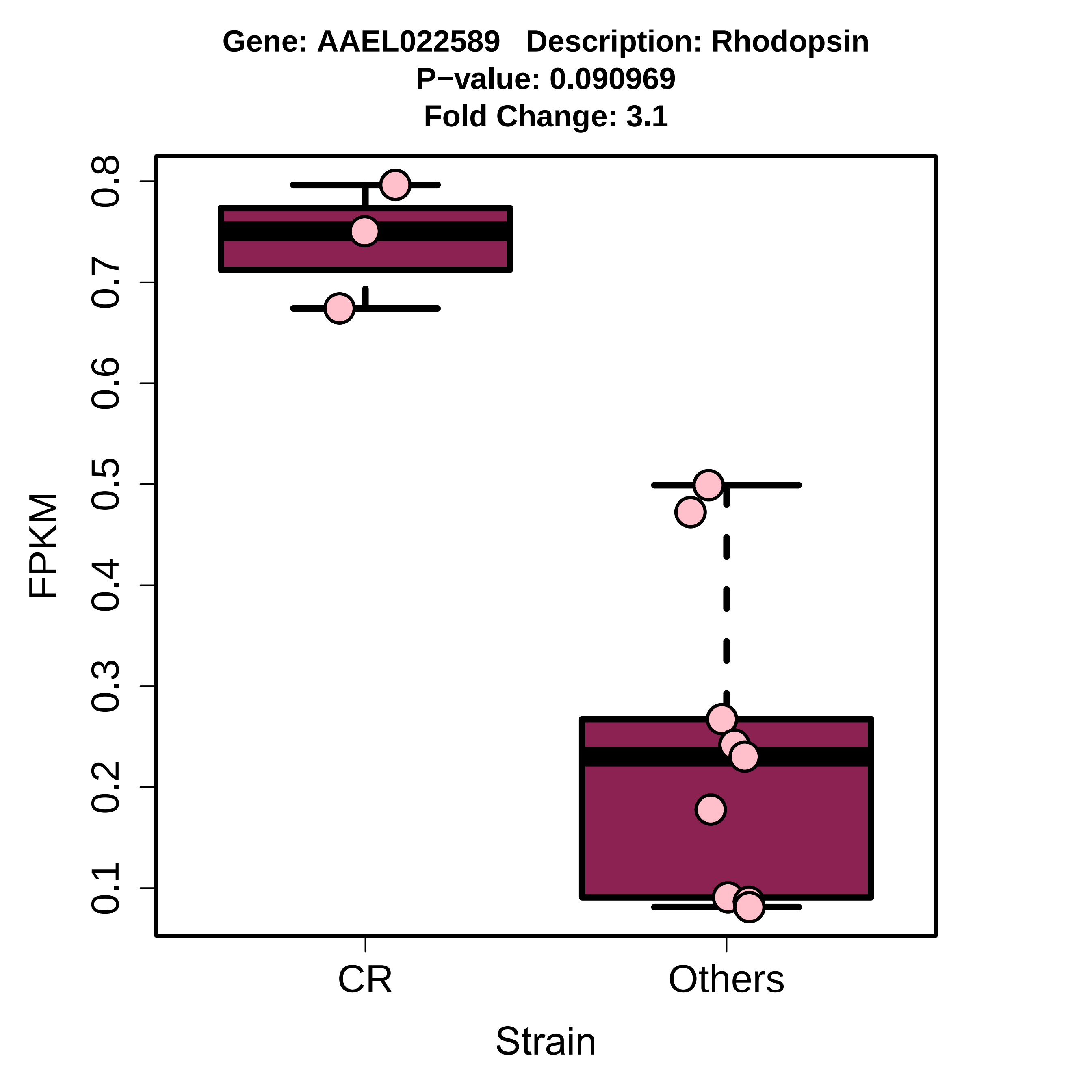
Gene: AAEL022589 Description: Rhodopsin
P−value: 0.090969
Fold Change: 3.1
FPKM
0.1
0.2
0.3
0.4
0.5
0.6
0.7
0.8
CR
Others
Strain

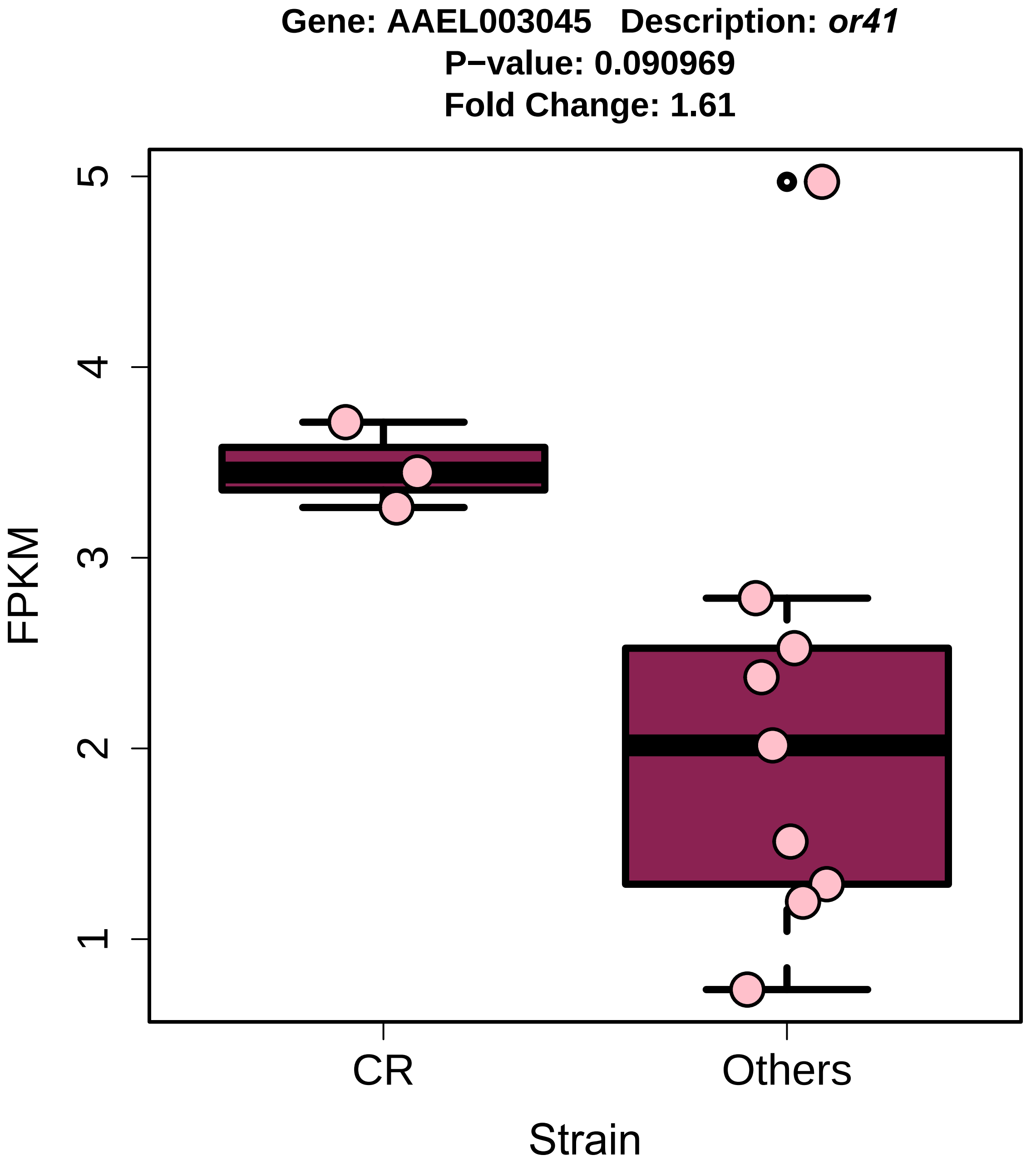

Gene: AAEL003045 Description: *or41*
P−value: 0.090969
Fold Change: 1.61
FPKM
1
2
3
4
5
CR
Others
Strain

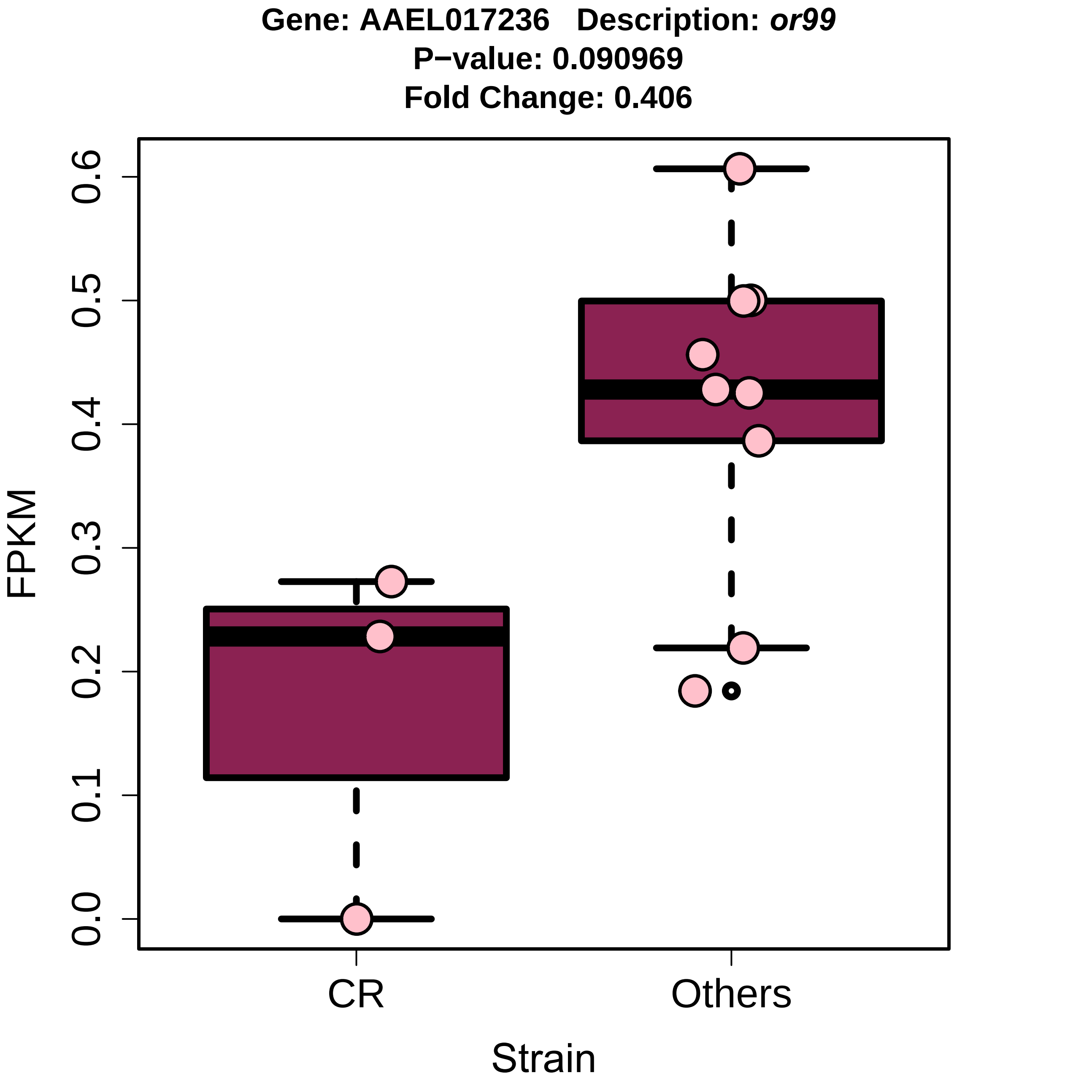

Gene: AAEL017236 Description: *or99*
P−value: 0.090969
Fold Change: 0.406
FPKM
0.0
0.1
0.2
0.3
0.4
0.5
0.6
CR
Others
Strain

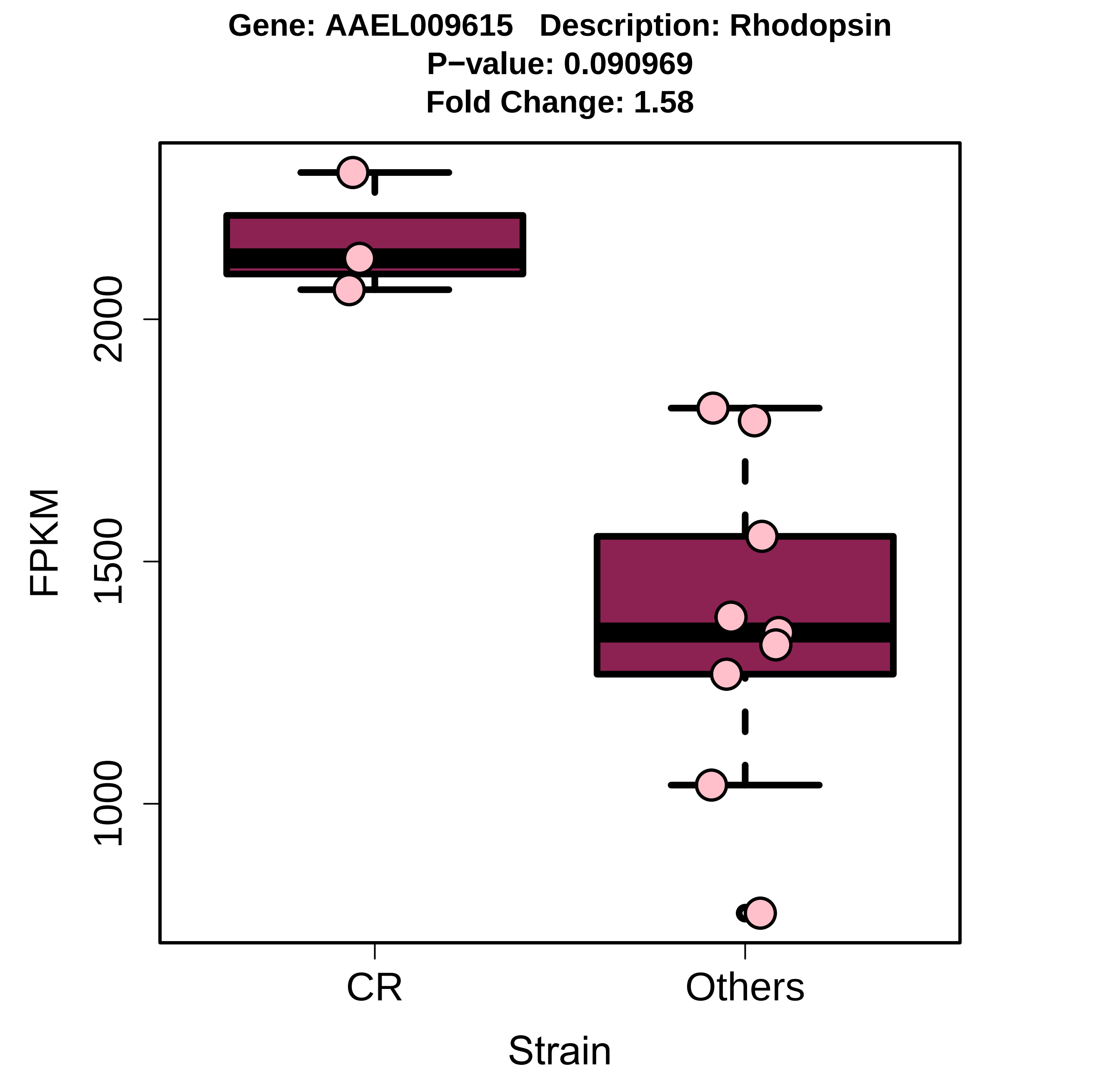

Gene: AAEL009615   Description: Rhodopsin
P−value: 0.090969
Fold Change: 1.58
FPKM
1000
1500
2000
CR
Others
Strain

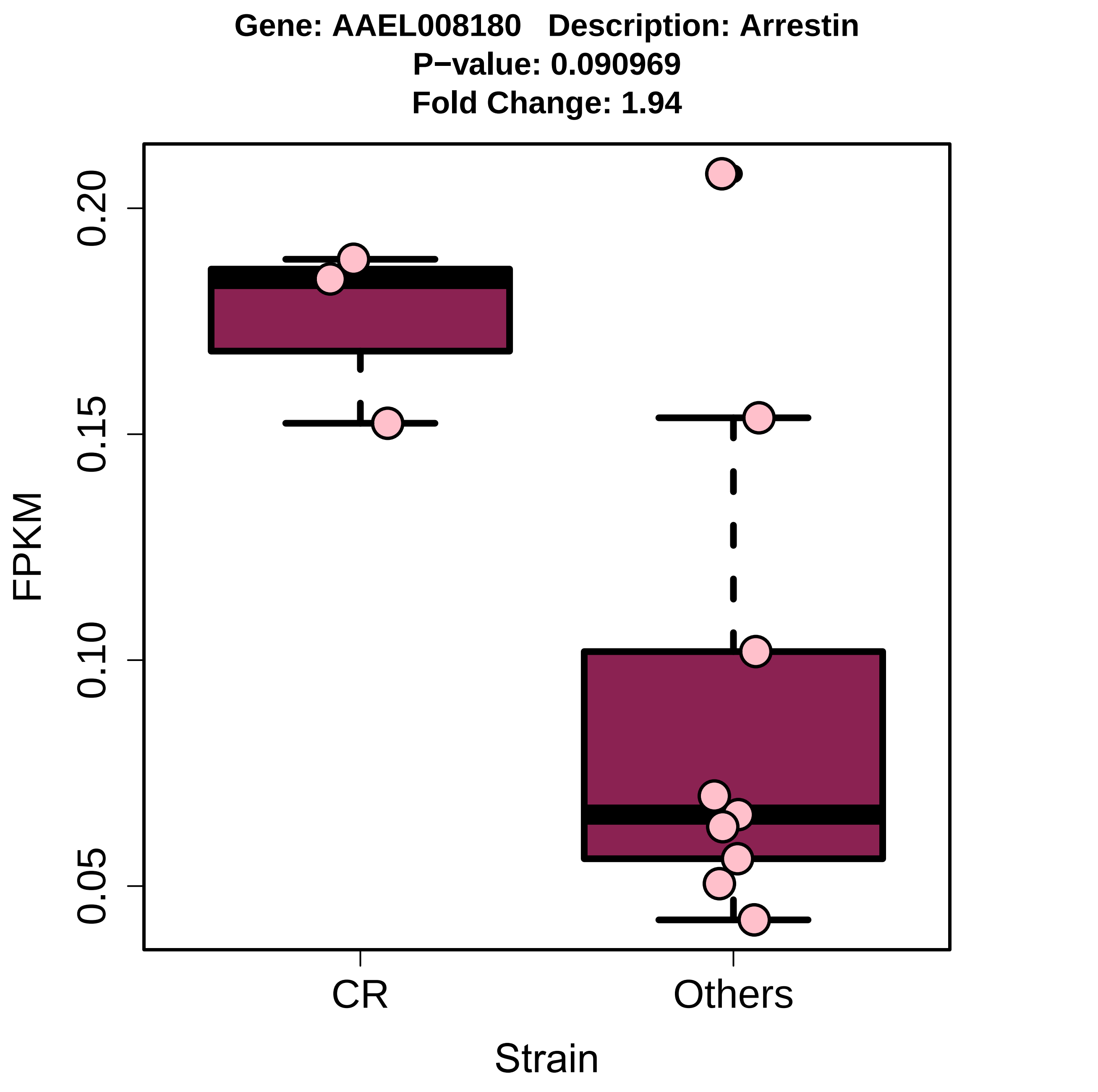
Gene: AAEL008180 Description: Arrestin
P−value: 0.090969
Fold Change: 1.94
0.20
0.15
0.10
0.05
FPKM
CR
Others
Strain

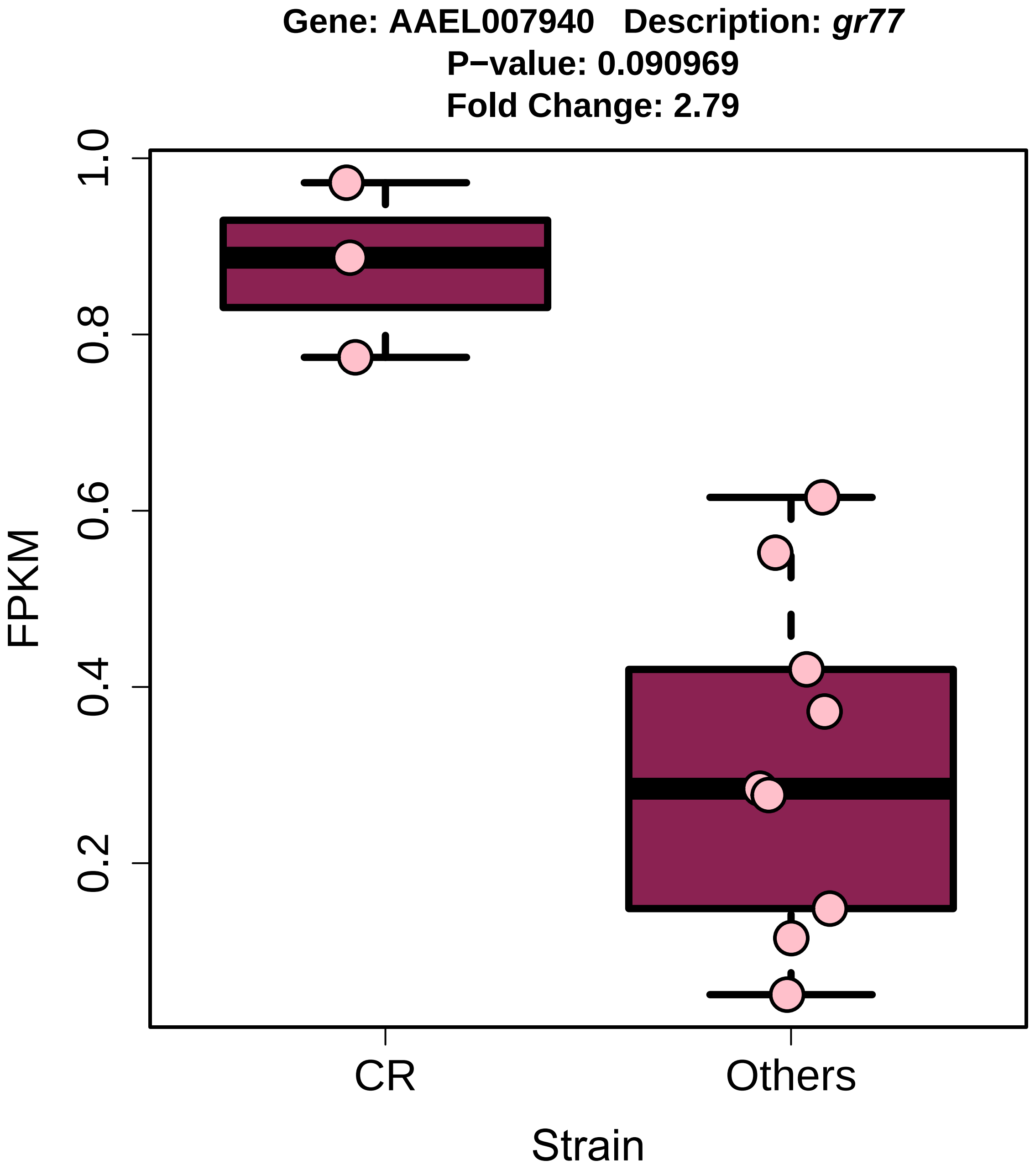
Gene: AAEL007940 Description: *gr77*
P−value: 0.090969
Fold Change: 2.79
1.0
0.8
0.6
0.4
0.2
FPKM
CR
Others
Strain

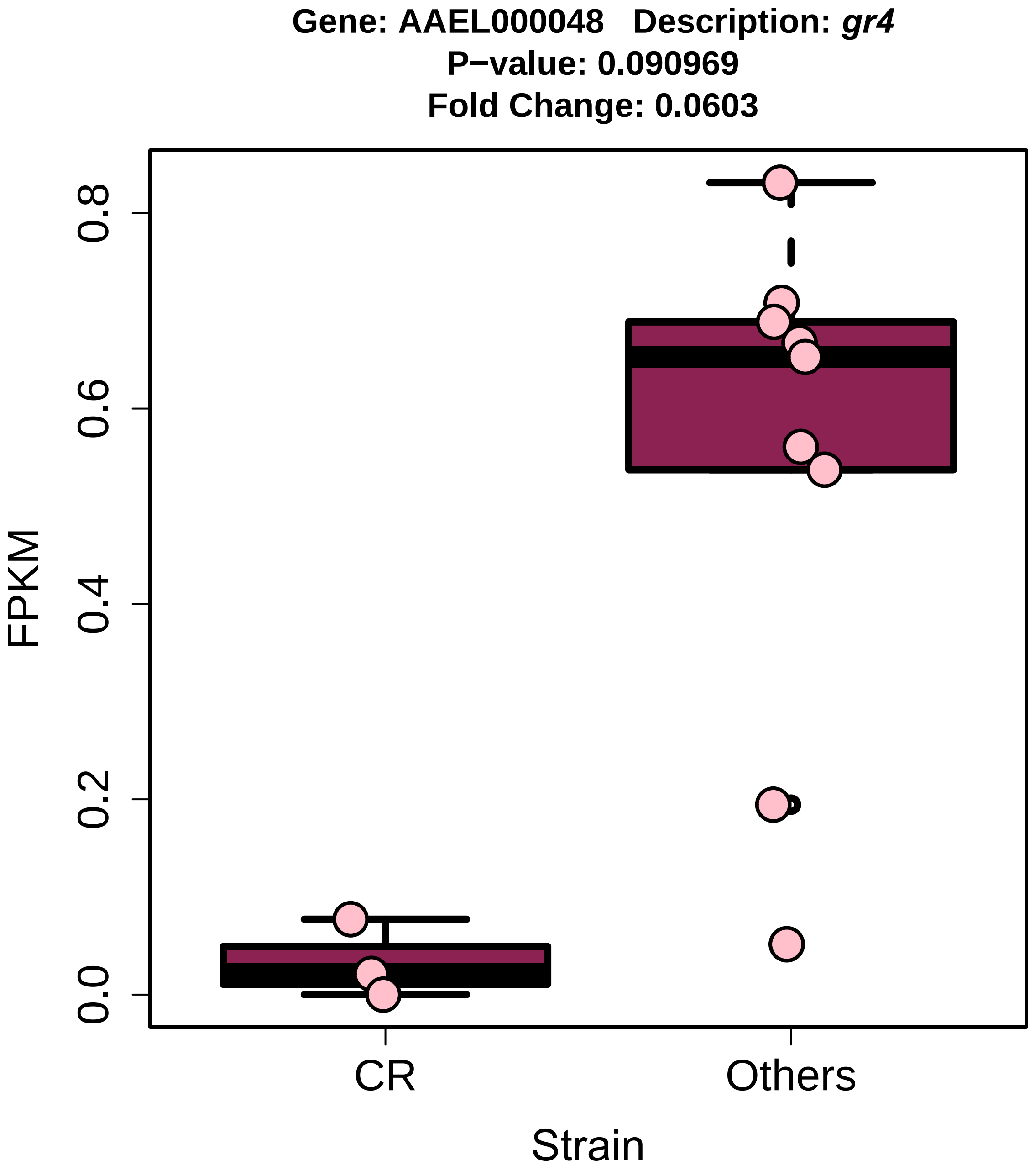
Gene: AAEL000048 Description: *gr4*
P−value: 0.090969
Fold Change: 0.0603
FPKM
0.0
0.2
0.4
0.6
0.8
CR
Others
Strain

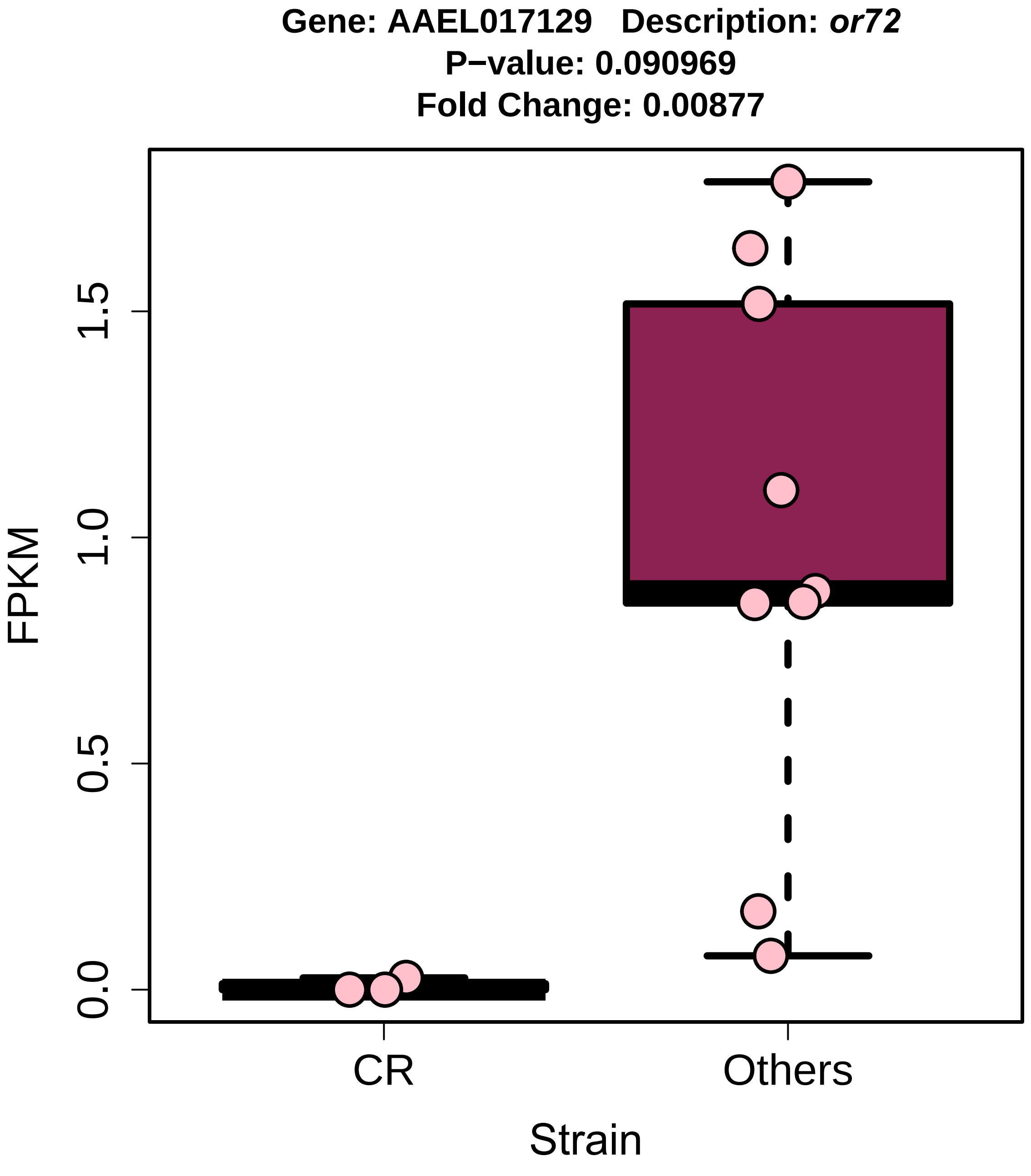

Gene: AAEL017129 Description: *or72*
P−value: 0.090969
Fold Change: 0.00877
FPKM
0.0
0.5
1.0
1.5
CR
Others
Strain
