# Supplementary material for: Olfaction-Related Gene Expression in the Antennae of Female Mosquitoes From Common Aedes aegypti Laboratory Strains
Source: Front Physiol. 2021 Aug 23;12:668236. doi: 10.3389/fphys.2021.668236 (PMC8419471; doi:10.3389/fphys.2021.668236)
Supplement: Supplementary file 7 [file Data_Sheet_4.pdf]

Supplemental File 8. Boxplots of top 20 olfaction-associated genes expressed differentially in PR compared to other strains. Boxplots represent interquartile range of expression from PR (n=3) versus the other three strains, whiskers represent first and fourth quartiles, and solid lines in boxes represent median expression values. Circles represent expression values from individual samples. Differential expression was determined using chi-squared goodness of fit tests, and $p<0.05$ represent significantly different genes.

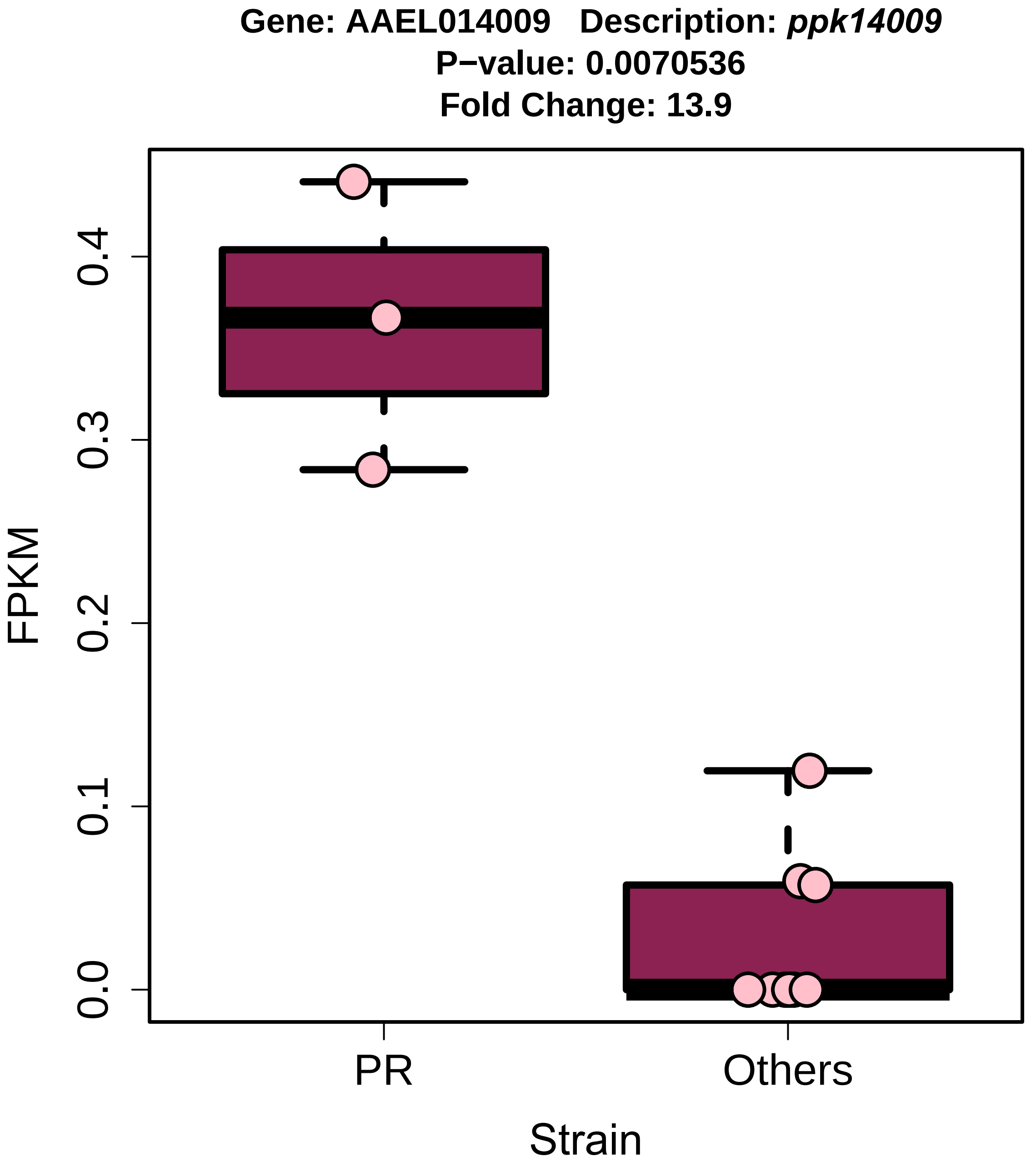

Gene: AAEL014009 Description: ppk14009
P−value: 0.0070536
Fold Change: 13.9
FPKM
0.0
0.1
0.2
0.3
0.4
PR
Others
Strain

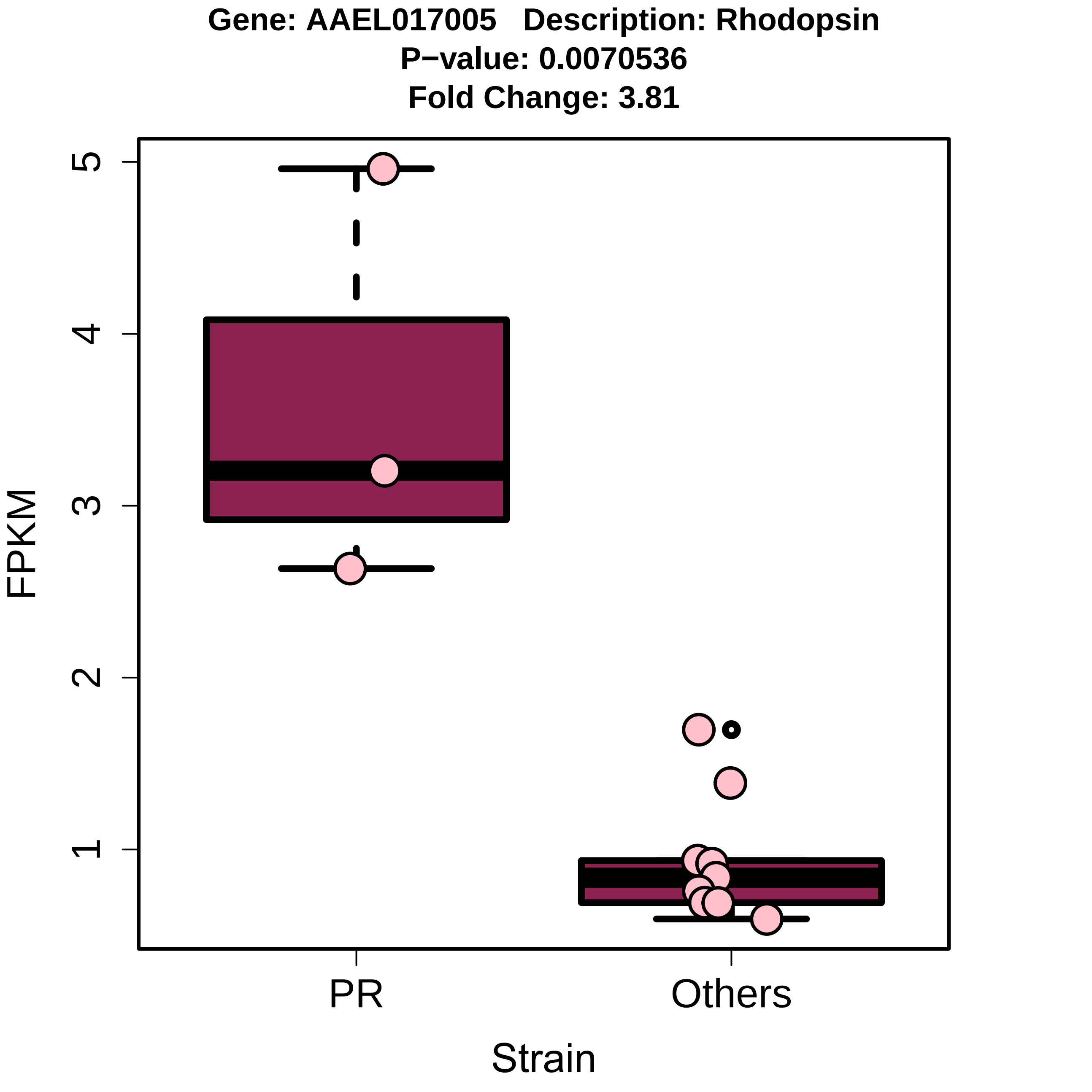
Gene: AAEL017005 Description: Rhodopsin
P−value: 0.0070536
Fold Change: 3.81
FPKM
5
4
3
2
1
PR
Others
Strain

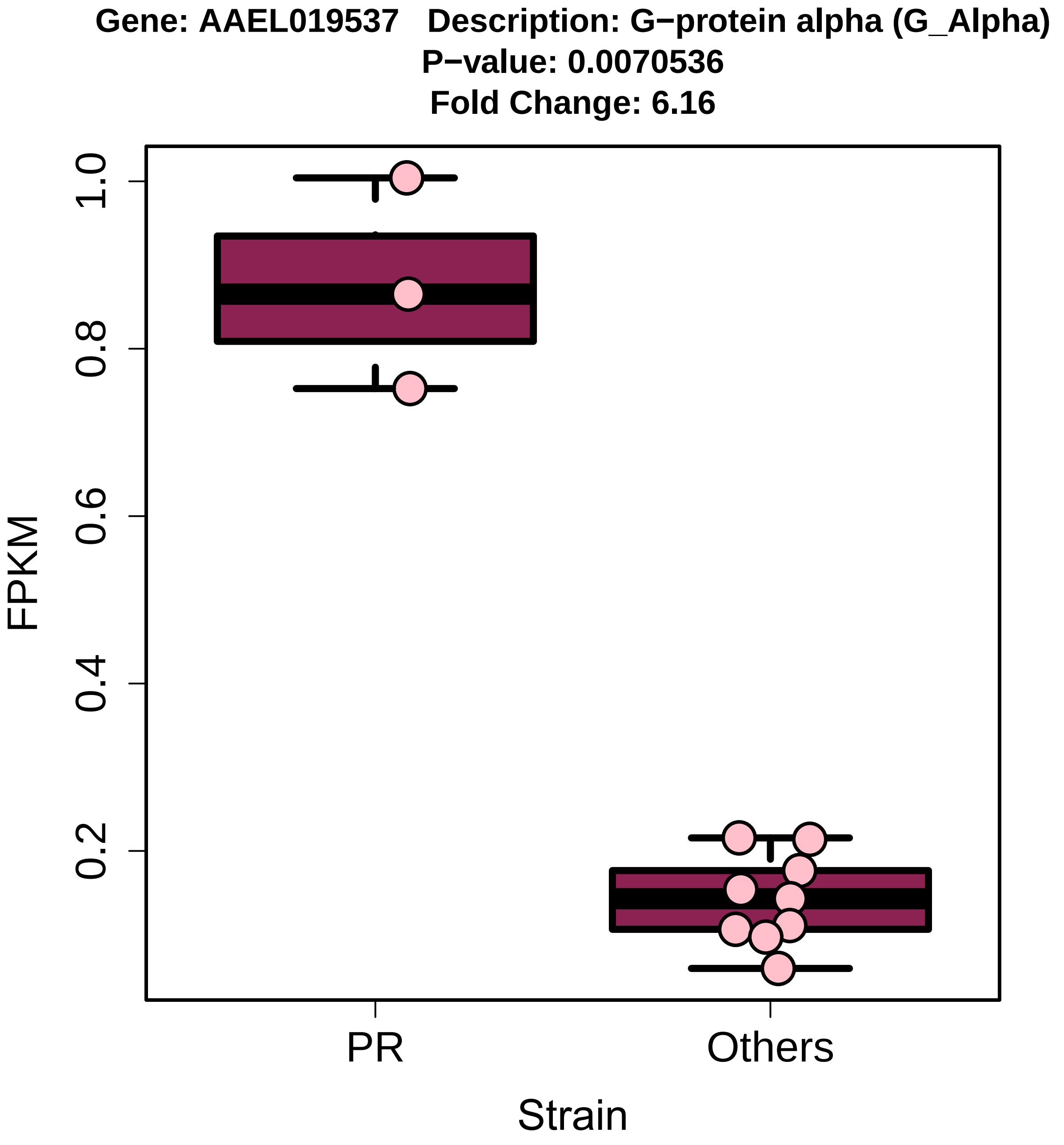
Gene: AAEL019537   Description: G−protein alpha (G_Alpha)
P−value: 0.0070536
Fold Change: 6.16
FPKM
1.0
0.8
0.6
0.4
0.2
PR
Others
Strain

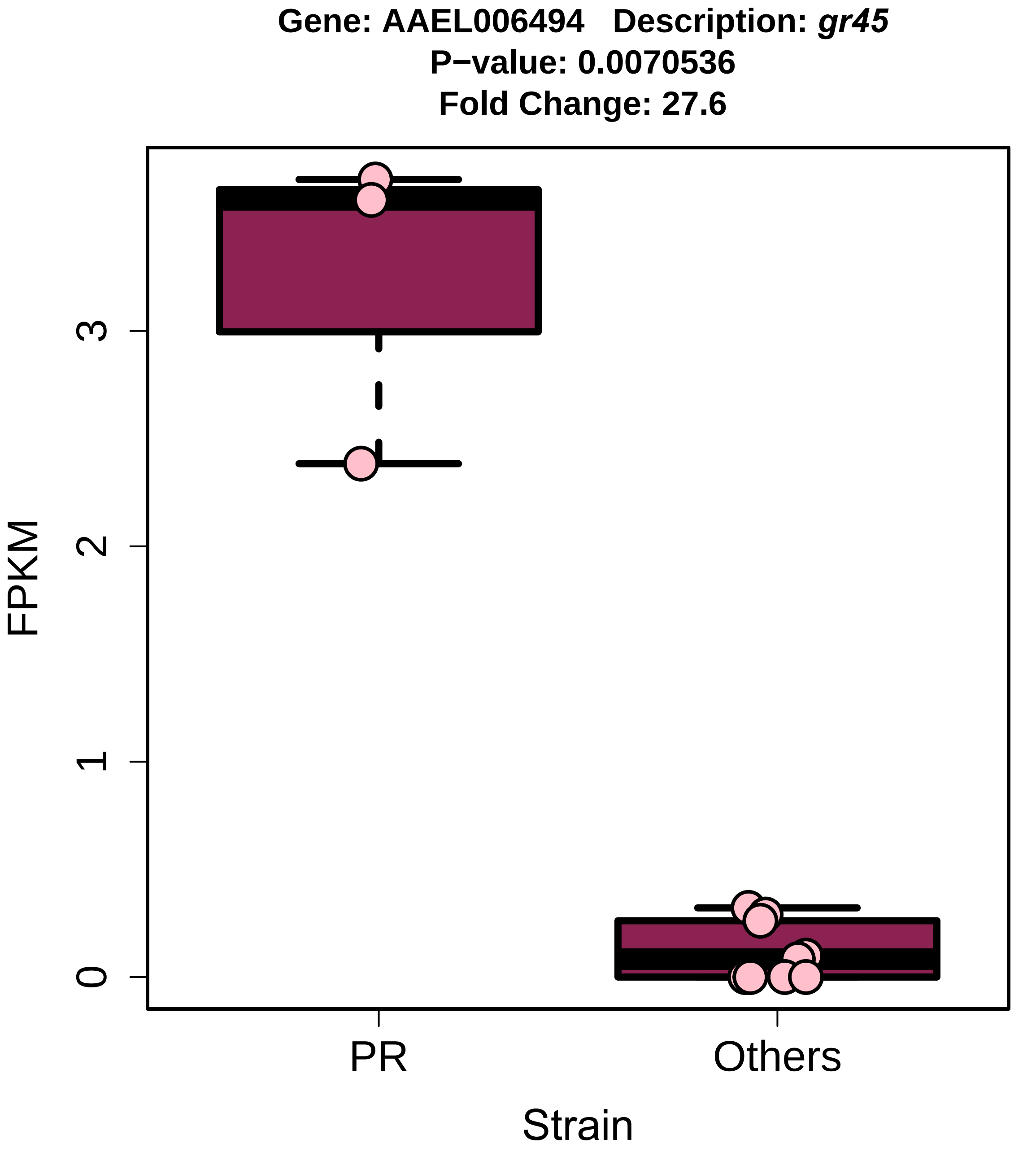

Gene: AAEL006494 Description: *gr45*
P−value: 0.0070536
Fold Change: 27.6
FPKM
0
1
2
3
PR
Others
Strain

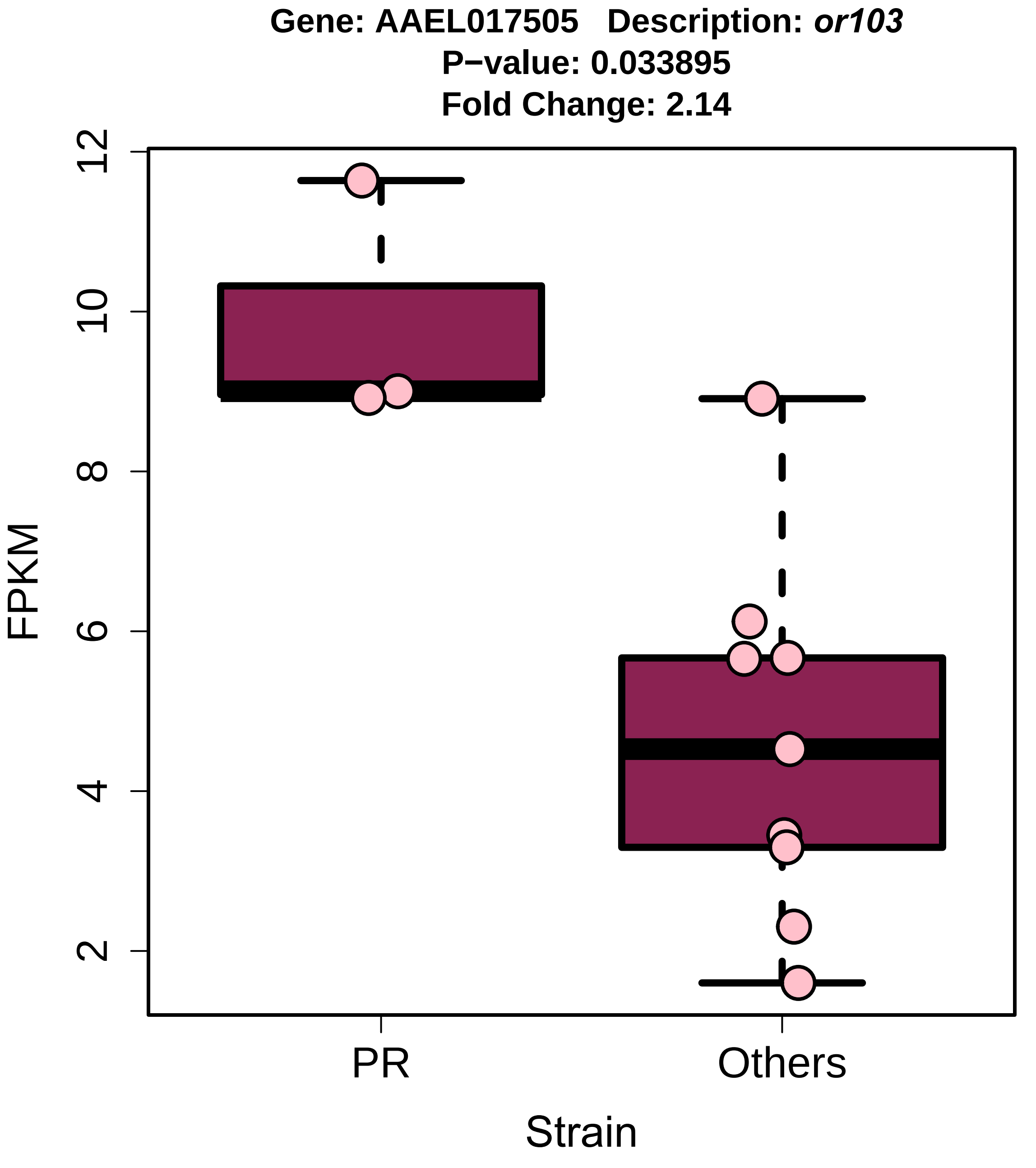
Gene: AAEL017505 Description: *or103*
P−value: 0.033895
Fold Change: 2.14
FPKM
12
10
8
6
4
2
PR
Others
Strain

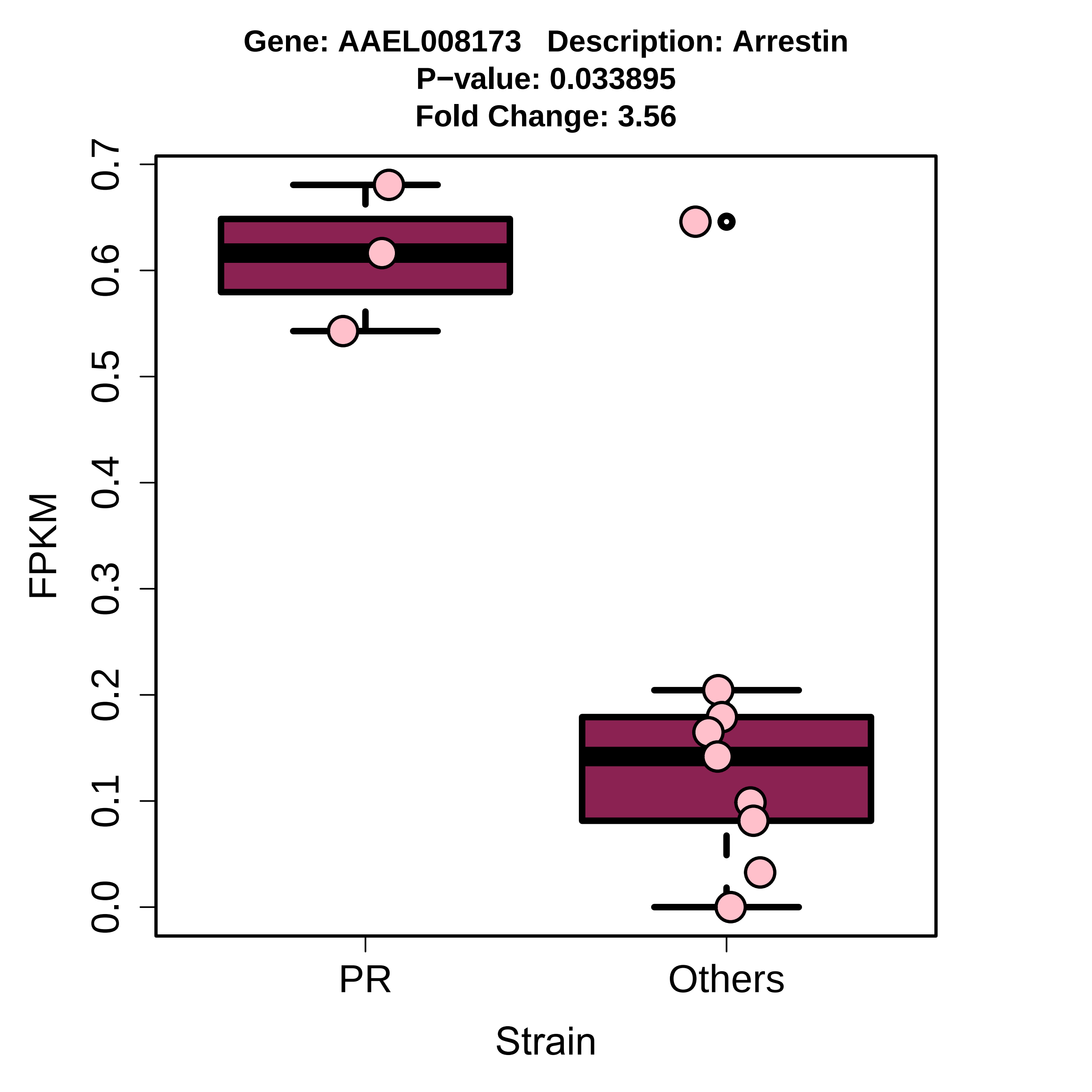
Gene: AAEL008173 Description: Arrestin
P−value: 0.033895
Fold Change: 3.56
FPKM
0.0
0.1
0.2
0.3
0.4
0.5
0.6
0.7
PR
Others
Strain

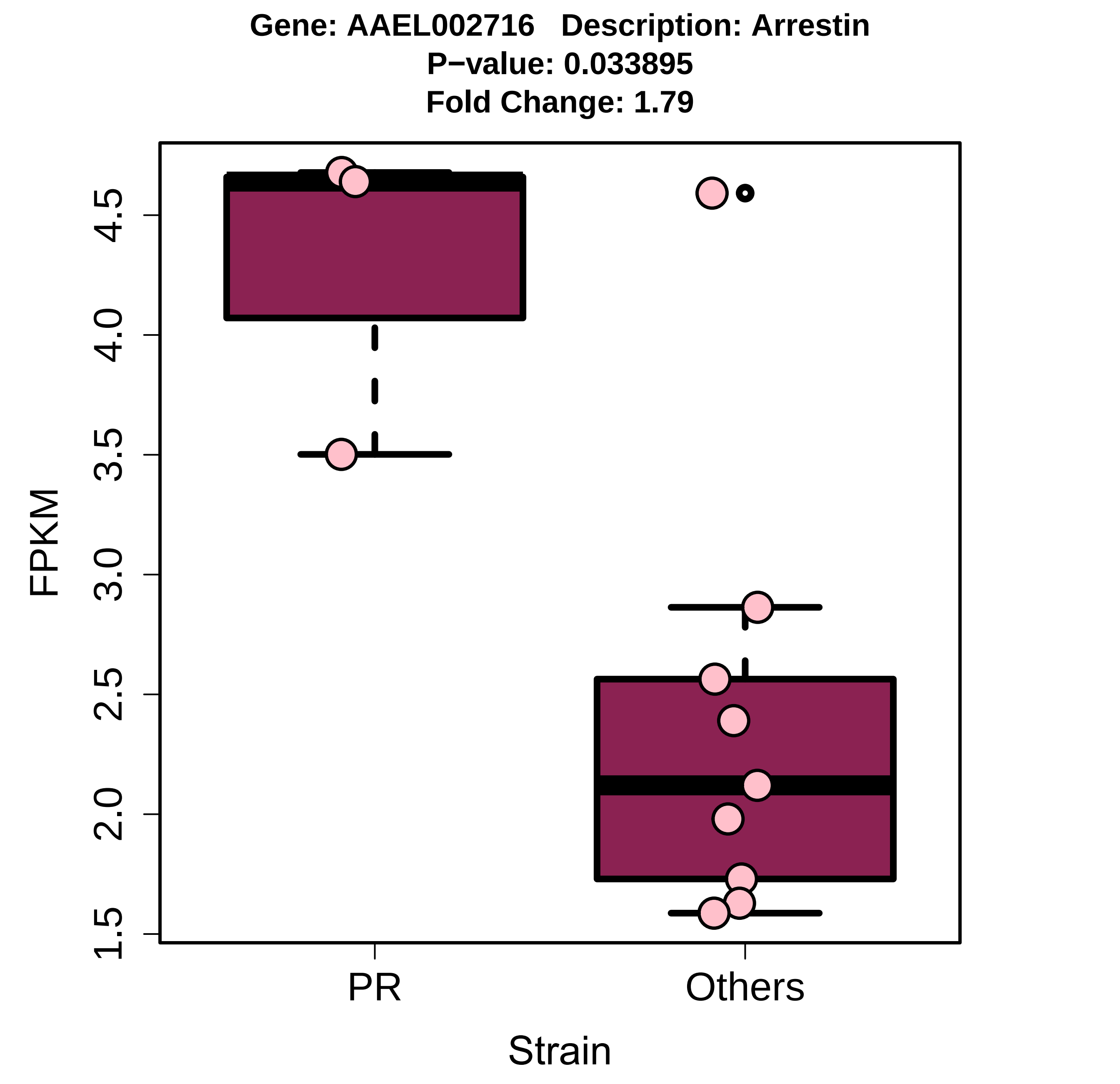

Gene: AAEL002716 Description: Arrestin
P−value: 0.033895
Fold Change: 1.79
FPKM
4.5
4.0
3.5
3.0
2.5
2.0
1.5
PR
Others
Strain

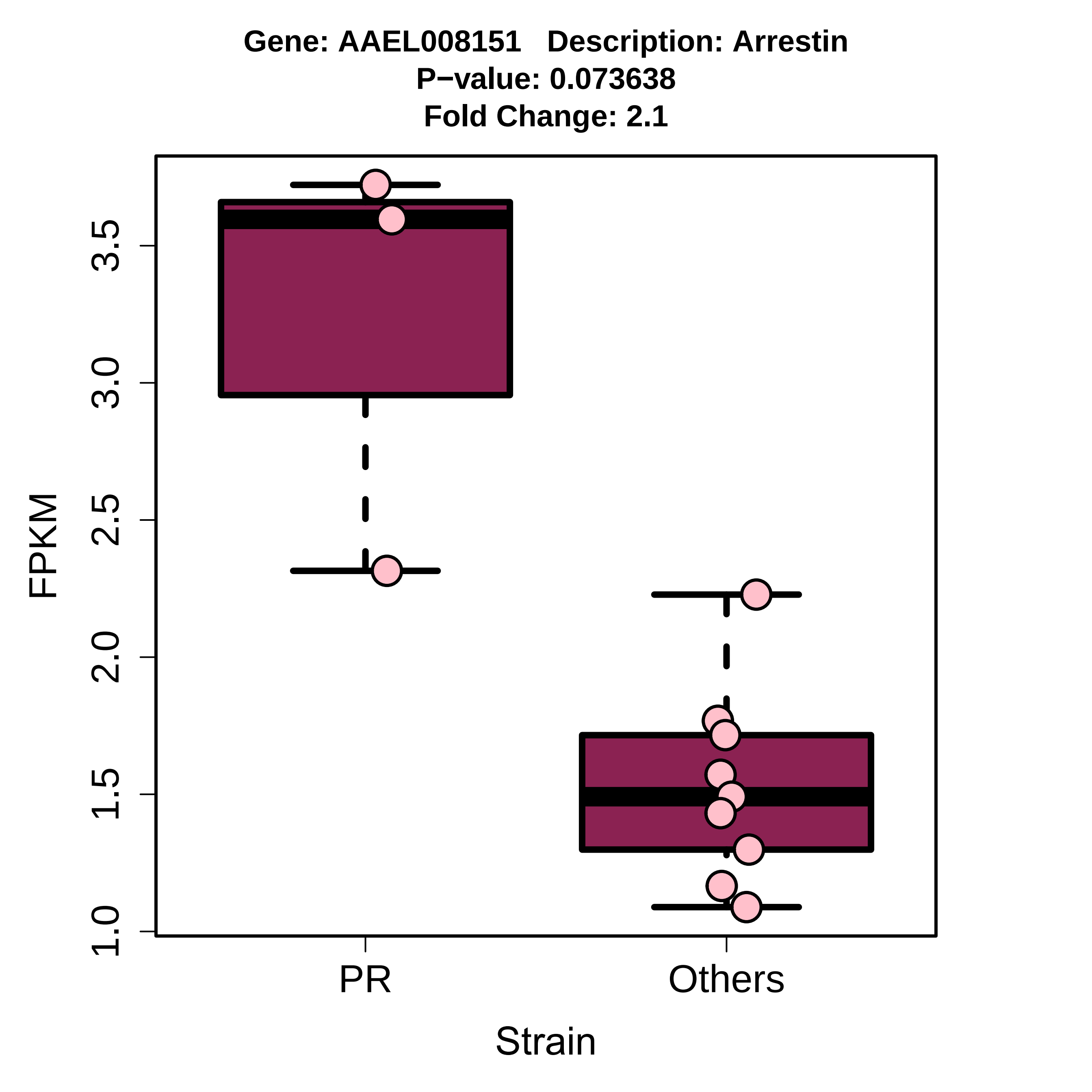

Gene: AAEL008151 Description: Arrestin
P−value: 0.073638
Fold Change: 2.1
FPKM
1.0
1.5
2.0
2.5
3.0
3.5
PR
Others
Strain

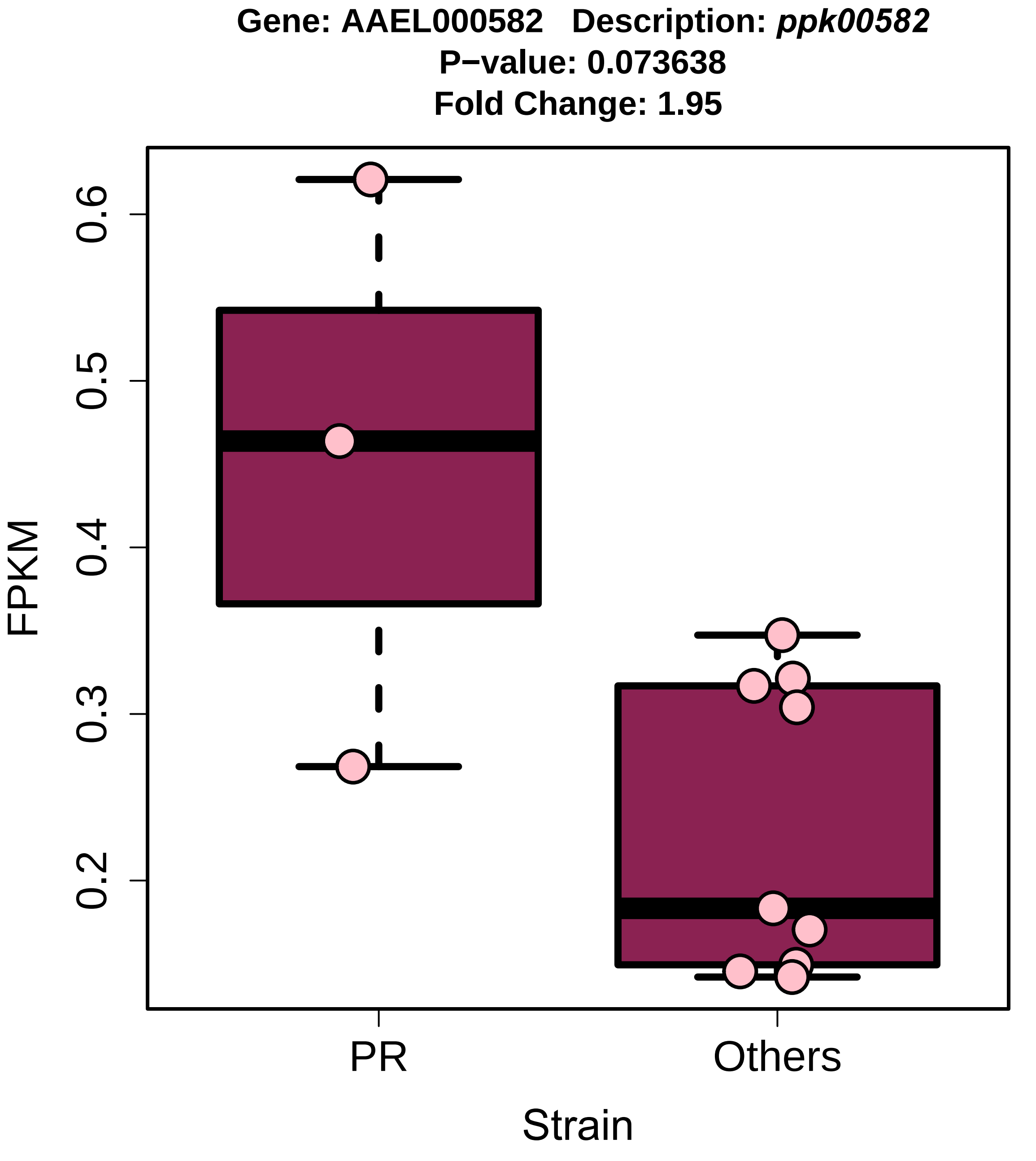

Gene: AAEL000582 Description: *ppk00582*
P−value: 0.073638
Fold Change: 1.95
FPKM
0.2
0.3
0.4
0.5
0.6
PR
Others
Strain

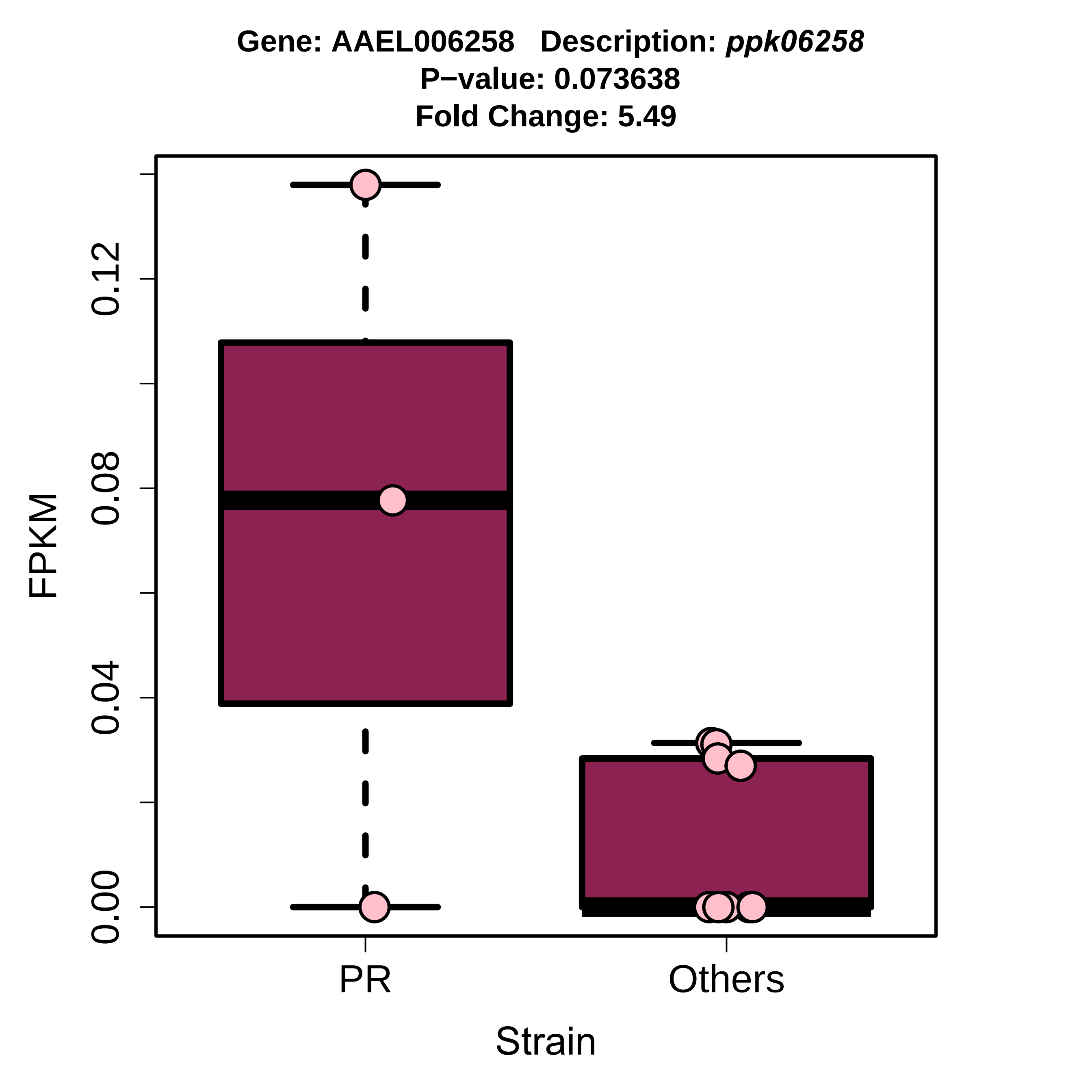
Gene: AAEL006258 Description: *ppk06258*
P−value: 0.073638
Fold Change: 5.49
FPKM
0.00
0.04
0.08
0.12
PR
Others
Strain

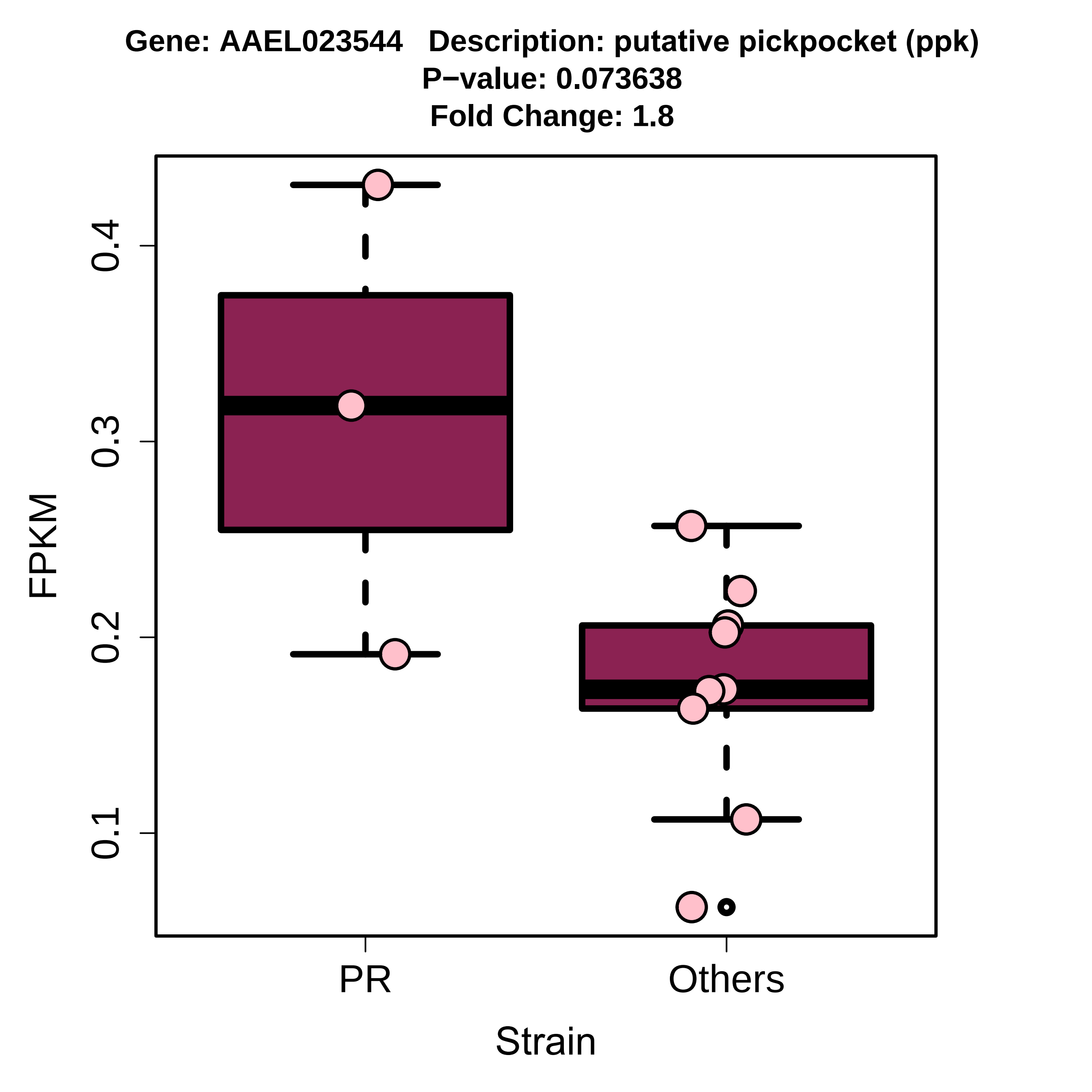
Gene: AAEL023544   Description: putative pickpocket (ppk)
P−value: 0.073638
Fold Change: 1.8
FPKM
0.1
0.2
0.3
0.4
PR
Others
Strain

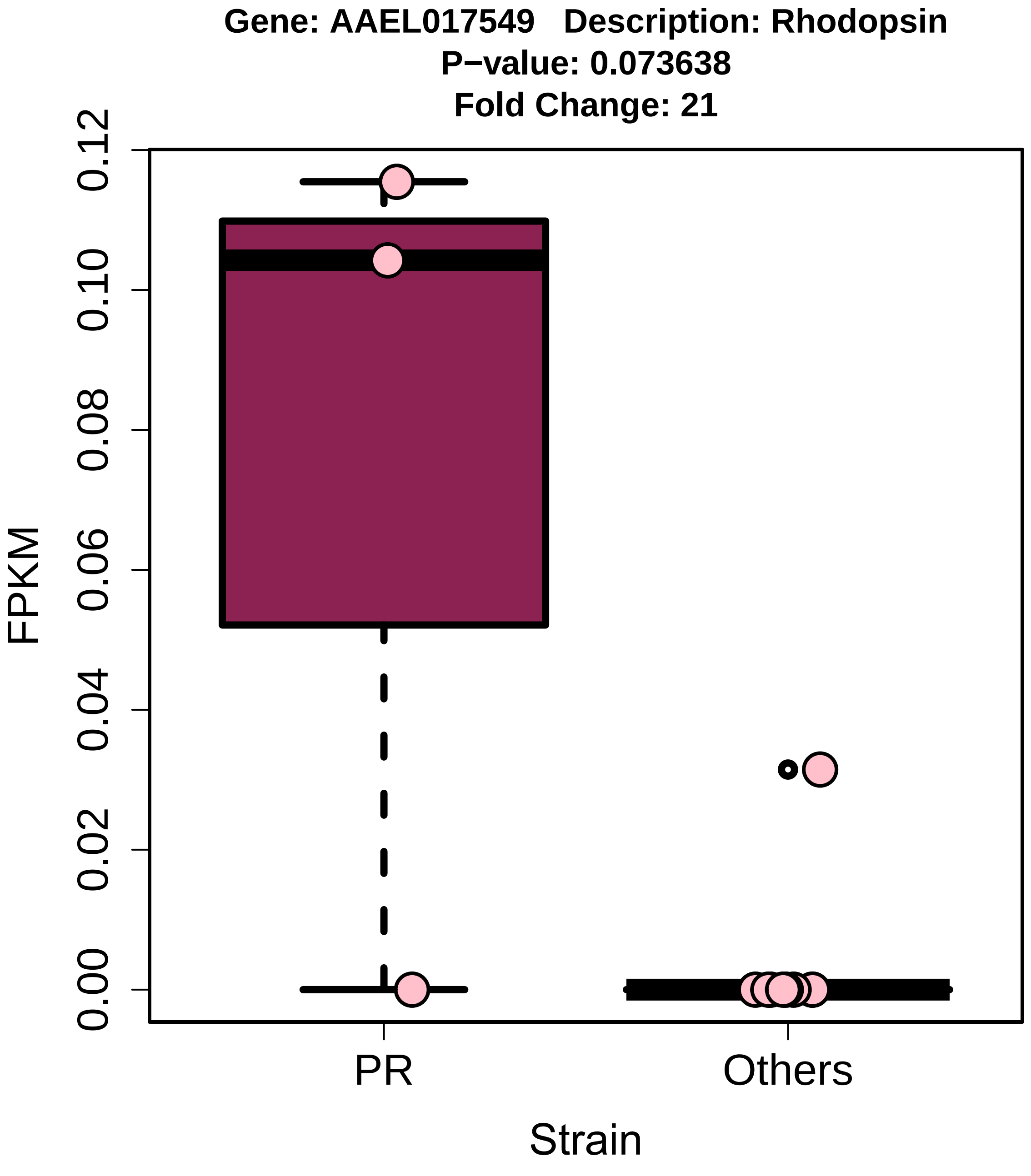
Gene: AAEL017549 Description: Rhodopsin
P−value: 0.073638
Fold Change: 21
FPKM
0.00
0.02
0.04
0.06
0.08
0.10
0.12
PR
Others
Strain

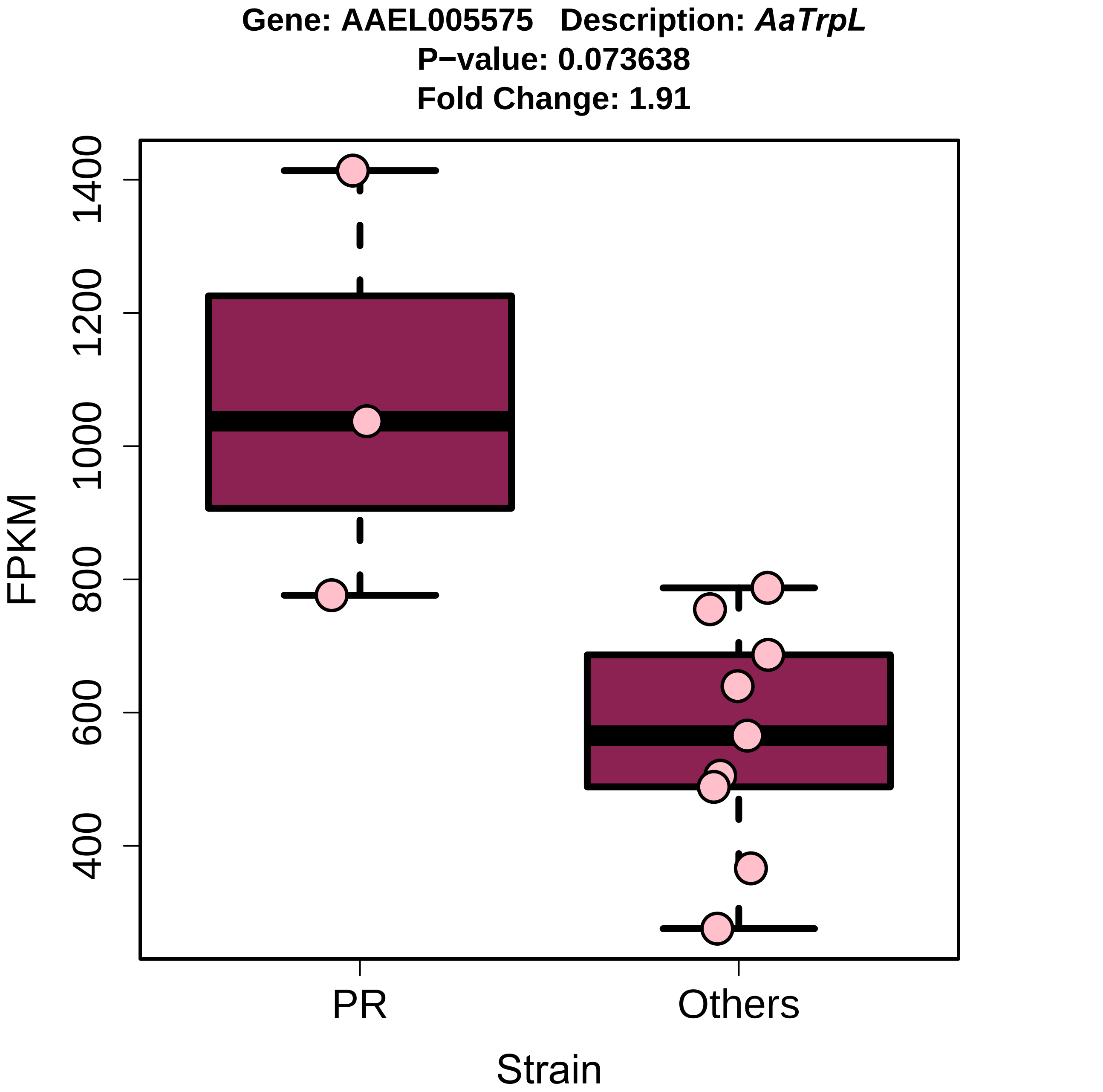
Gene: AAEL005575 Description: *AaTrpL*
P−value: 0.073638
Fold Change: 1.91
FPKM
400
600
800
1000
1200
1400
PR
Others
Strain

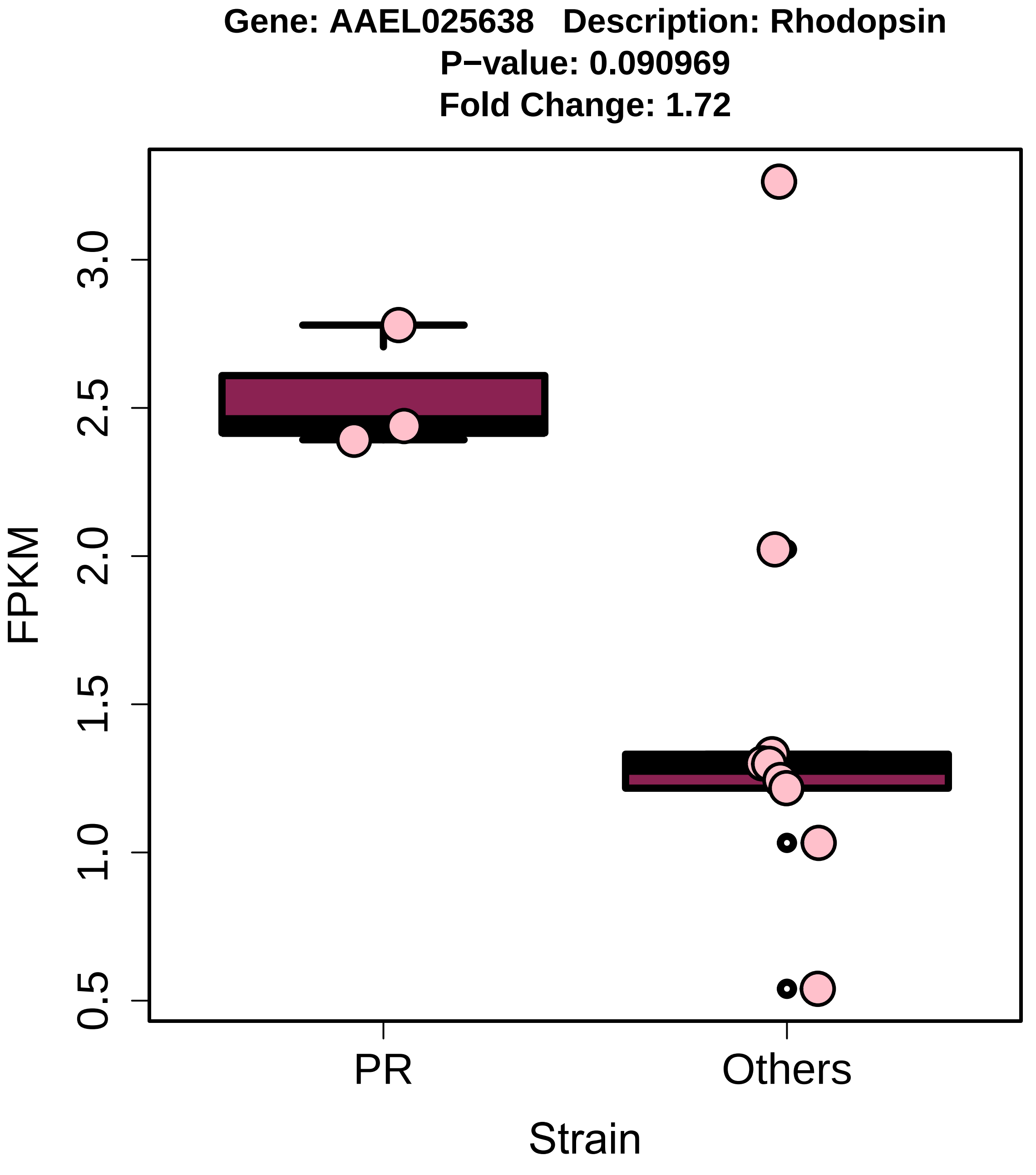
Gene: AAEL025638 Description: Rhodopsin
P−value: 0.090969
Fold Change: 1.72
FPKM
3.0
2.5
2.0
1.5
1.0
0.5
PR
Others
Strain

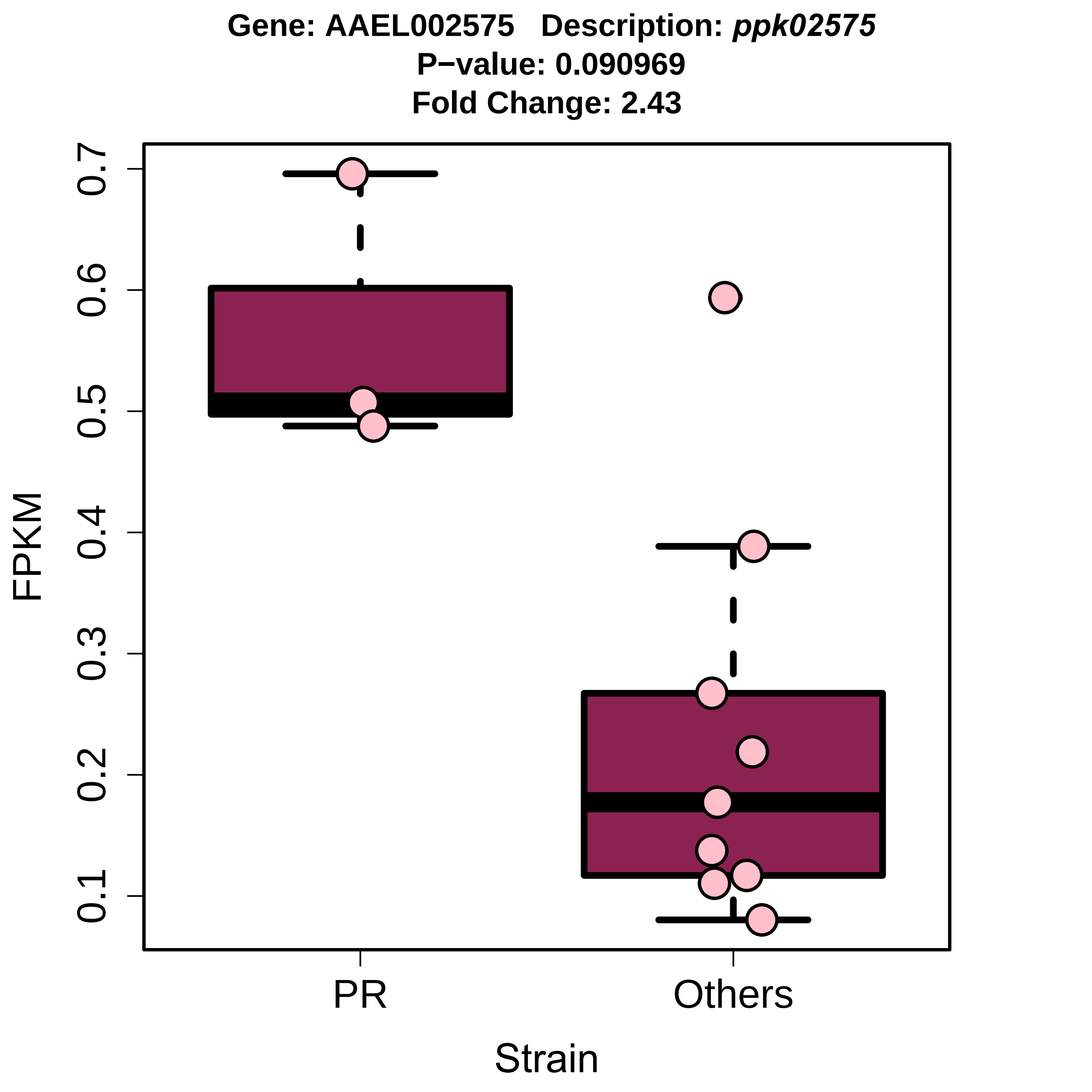
Gene: AAEL002575 Description: *ppk02575*
P−value: 0.090969
Fold Change: 2.43
FPKM
0.1
0.2
0.3
0.4
0.5
0.6
0.7
PR
Others
Strain

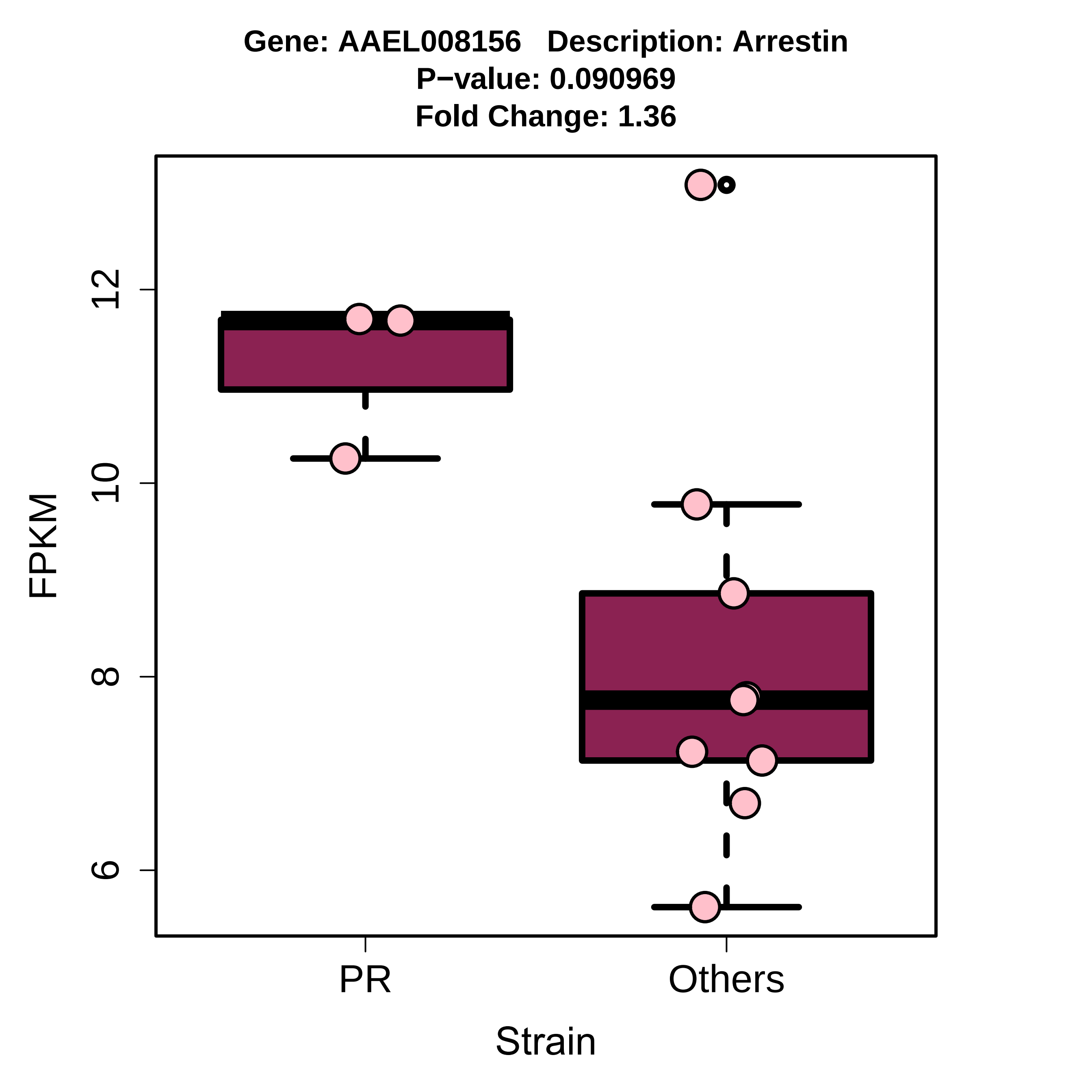

Gene: AAEL008156 Description: Arrestin
P−value: 0.090969
Fold Change: 1.36
FPKM
6
8
10
12
PR
Others
Strain

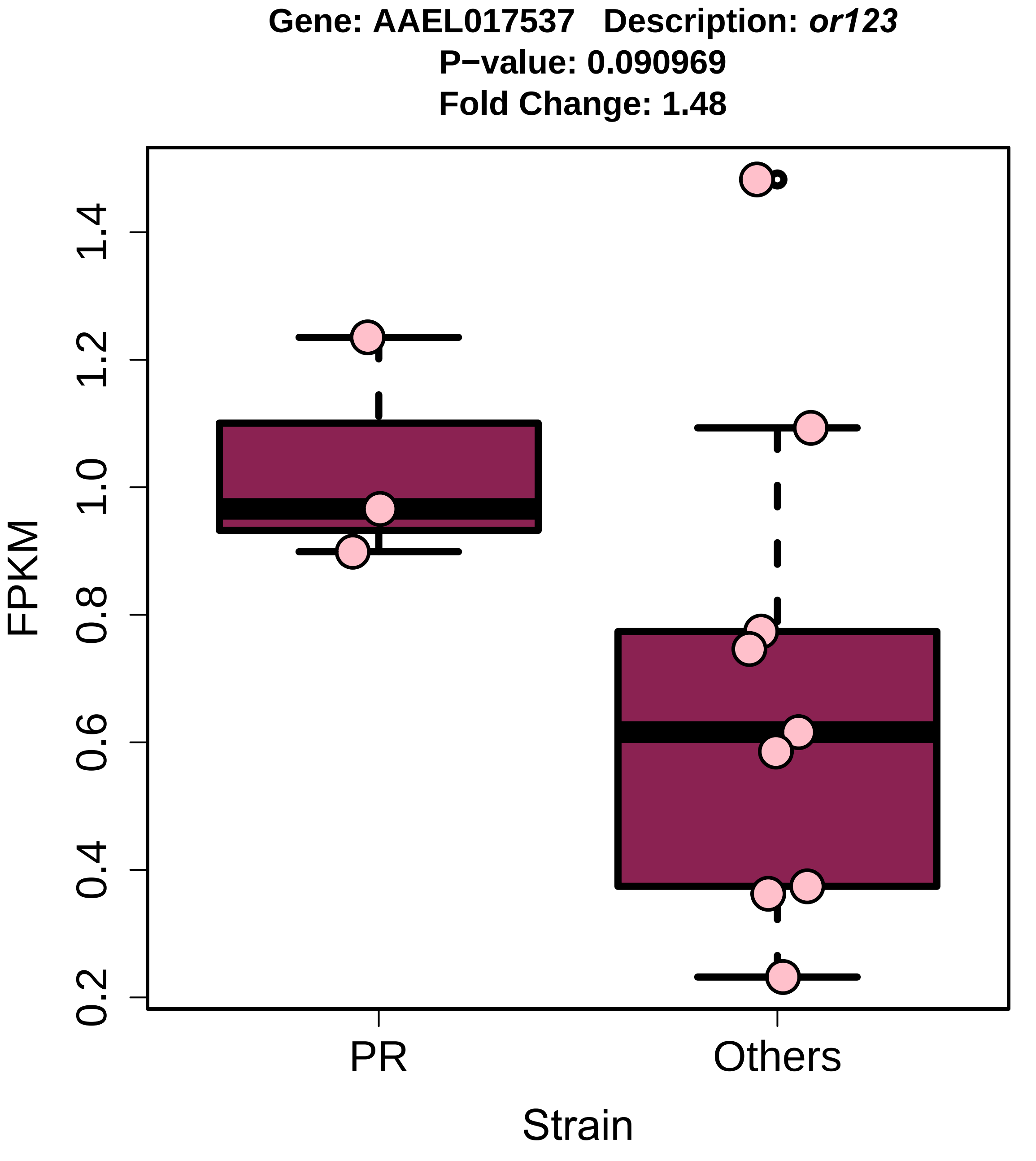
Gene: AAEL017537 Description: *or123*
P−value: 0.090969
Fold Change: 1.48
FPKM
0.2
0.4
0.6
0.8
1.0
1.2
1.4
PR
Others
Strain

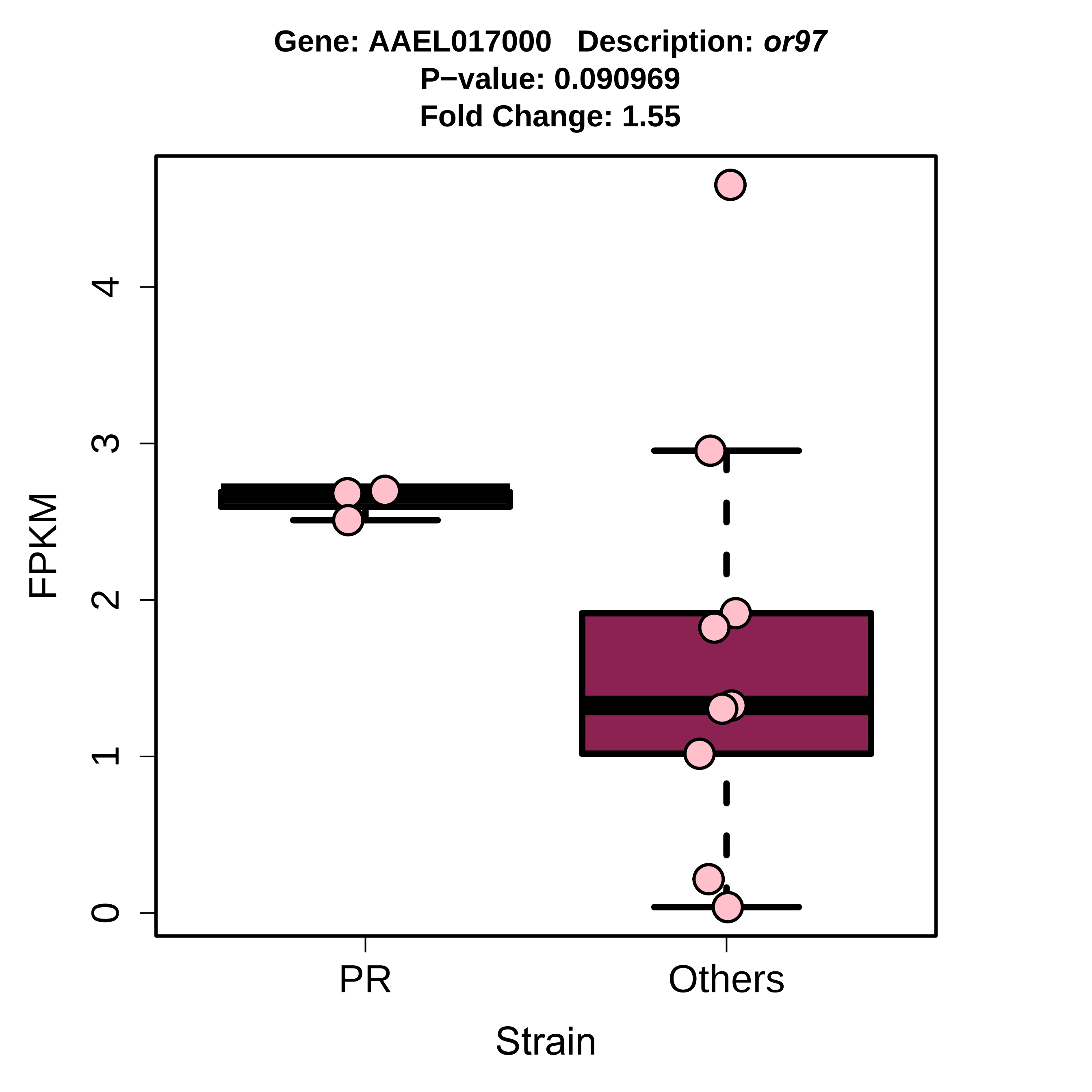

Gene: AAEL017000 Description: *or97*
P−value: 0.090969
Fold Change: 1.55
FPKM
0
1
2
3
4
PR
Others
Strain

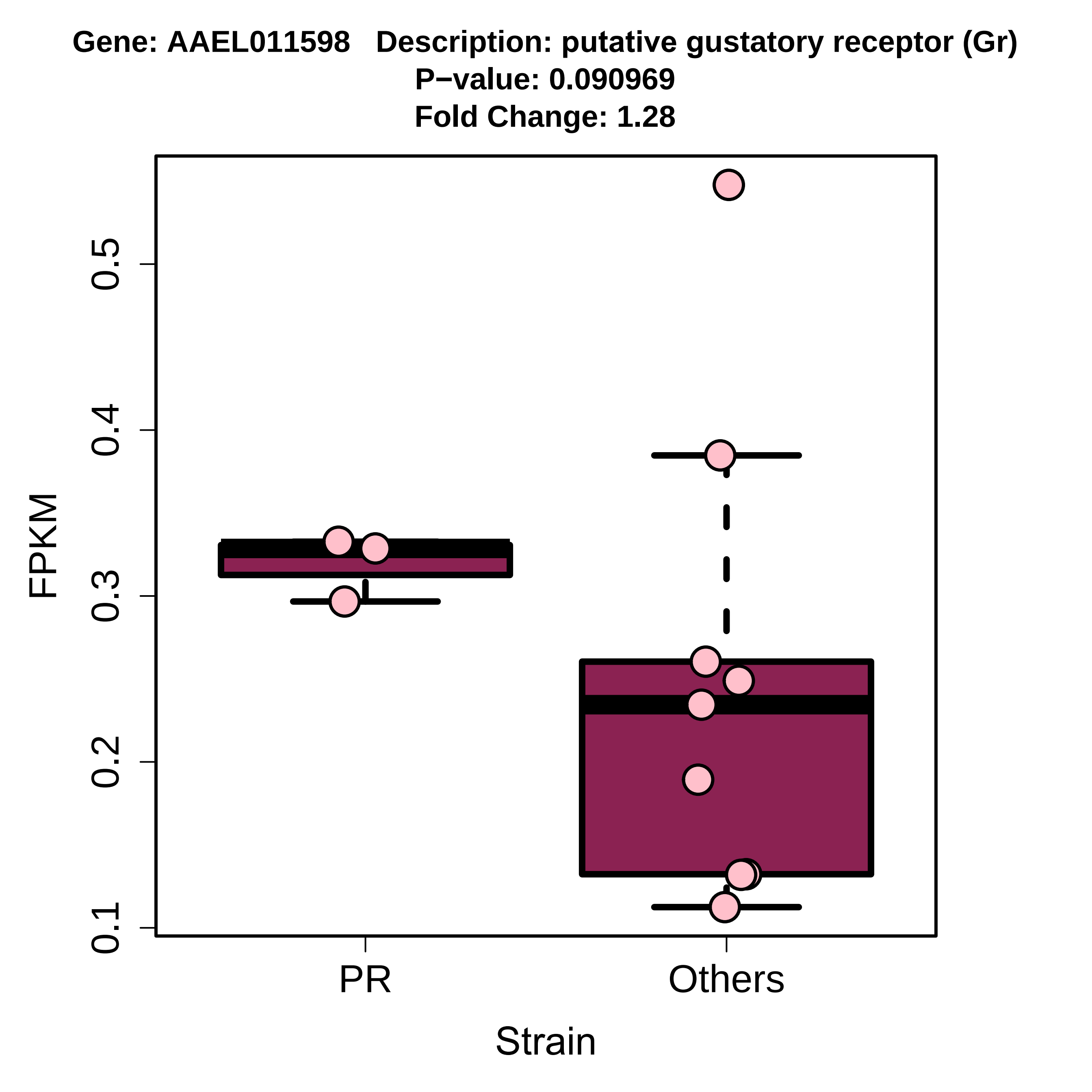
Gene: AAEL011598 Description: putative gustatory receptor (Gr)
P−value: 0.090969
Fold Change: 1.28
FPKM
0.1
0.2
0.3
0.4
0.5
PR
Others
Strain

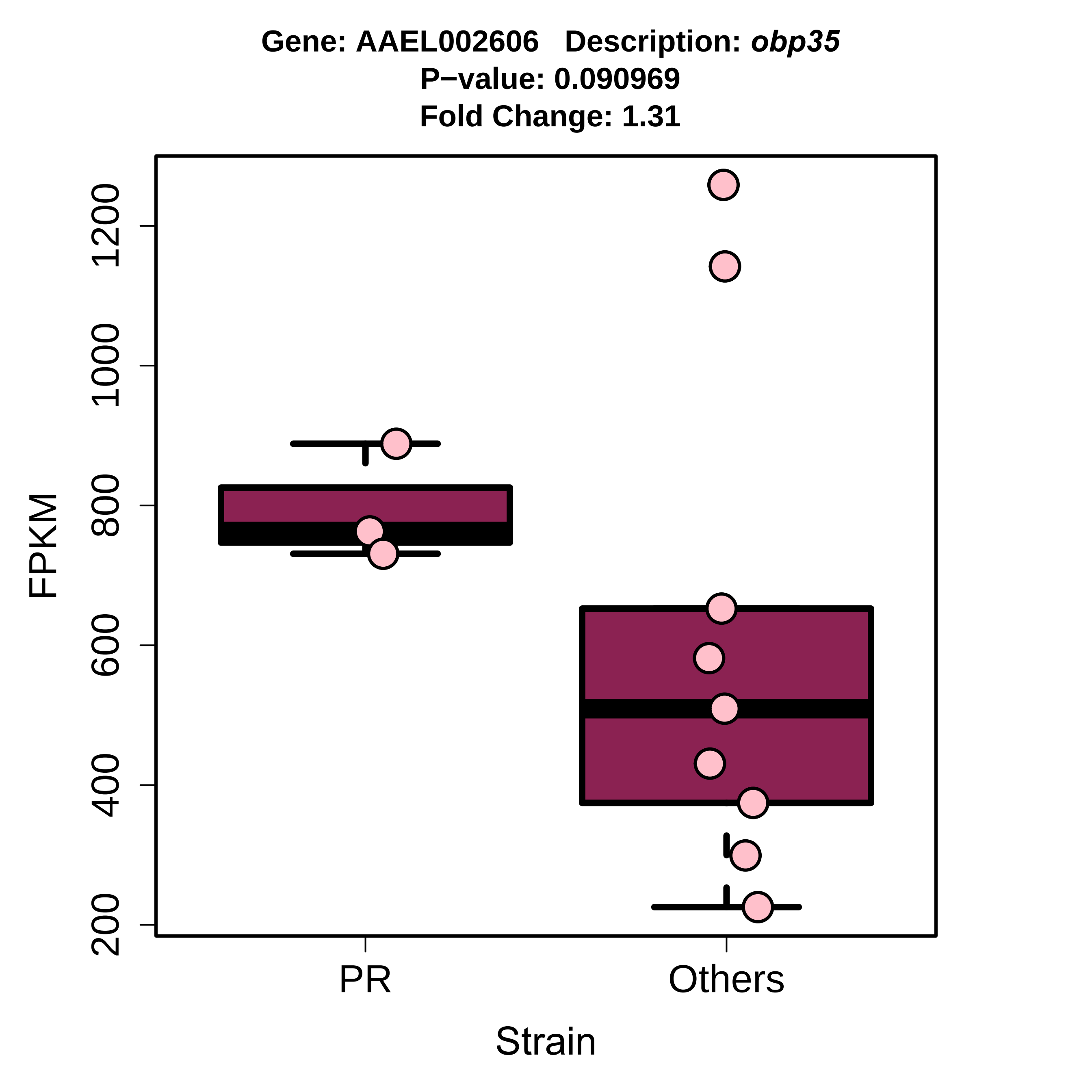
Gene: AAEL002606   Description: *obp35*
P−value: 0.090969
Fold Change: 1.31
FPKM
200
400
600
800
1000
1200
PR
Others
Strain
