# Supplementary material for: Olfaction-Related Gene Expression in the Antennae of Female Mosquitoes From Common Aedes aegypti Laboratory Strains
Source: Front Physiol. 2021 Aug 23;12:668236. doi: 10.3389/fphys.2021.668236 (PMC8419471; doi:10.3389/fphys.2021.668236)
Supplement: Supplementary file 9 [file Image_1.pdf]

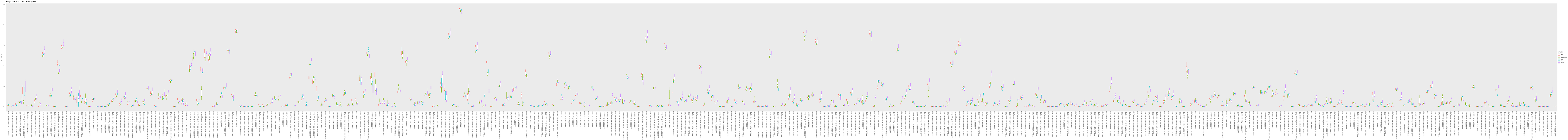

Supplemental File 4. Boxplots of antennal sensory transcriptome of four *Ae. aegypti* strains: Rock, Liverpool, CR, PR. Boxplots represent interquartile range of expression from each strain (n=3), whiskers represent first and fourth quartiles. Solid lines in boxes represent median expression values.
