# Supplementary material for: Olfaction-Related Gene Expression in the Antennae of Female Mosquitoes From Common Aedes aegypti Laboratory Strains
Source: Front Physiol. 2021 Aug 23;12:668236. doi: 10.3389/fphys.2021.668236 (PMC8419471; doi:10.3389/fphys.2021.668236)
Supplement: Supplementary file 8 [file Data_Sheet_5.pdf]

Supplemental File 9. Quantitative RT-PCR analysis of three olfactory related genes confirms RNA-seq expression profile. (A) Reference gene selection using RefFinder (Xie et al. 2012). Cq values of three odor sensing genes (*or26*, *or97*, and *obp56a*) and two candidate reference genes (*β-actin* and *rps7*) from the qRT-PCR runs were averaged across technical replicates, and then entered. This graph represents the consensus output of these four algorithms (displayed as geometric means of the outputs of the algorithms) showing *rps7* as most suitable reference gene. (B) qRT-PCR data for three odor sensing genes normalized to rps7 expression levels. Raw fluorescence data was imported into the Real-Time PCR Miner tool (Zhao and Fernald 2005) and analyzed to determine primer amplification efficiencies. These efficiencies were used along with Cq values from the BioRad CFX96 platform and normalized to *rps7* data to calculate relative mRNA level. Each white bar represents the average relative mRNA level from each strain (n=3) ± standard error. Letters in bars represent statistical significance groups based on one-way ANOVA analysis with Tukey's multiple comparisons post-hoc tests to determine significant differences ($p<0.05$) in relative mRNA levels between strains. Each gray bar represents the corresponding FPKM value from the RNA-seq dataset (n=3) ± standard error. Note: qRT-PCR data are graphed on the primary y-axis, and corresponding RNA-seq FPKM values are graphed on the secondary y-axis. Axis values differ between graphs.

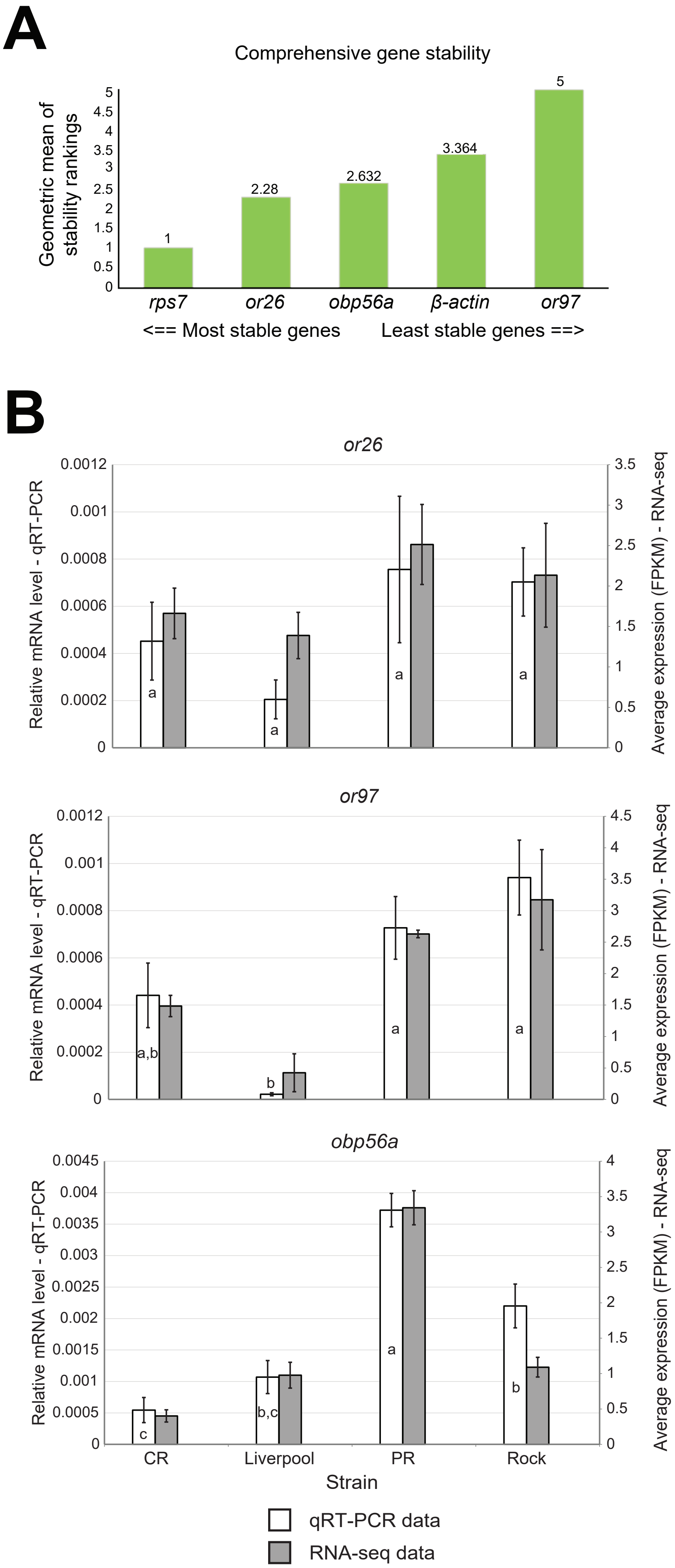
A
Comprehensive gene stability
Geometric mean of stability rankings
1
2.28
2.632
3.364
5
rps7
or26
obp56a
β-actin
or97
<== Most stable genes
Least stable genes ==>
B
or26
or97
obp56a
Relative mRNA level - qRT-PCR
Average expression (FPKM) - RNA-seq
a
a,b
b
b,c
c
CR
Liverpool
PR
Rock
Strain
qRT-PCR data
RNA-seq data
